# Isoquercitrin Esters with Mono- or Dicarboxylic Acids: Enzymatic Preparation and Properties

**DOI:** 10.3390/ijms17060899

**Published:** 2016-06-07

**Authors:** Eva Vavříková, Fanny Langschwager, Lubica Jezova-Kalachova, Alena Křenková, Barbora Mikulová, Marek Kuzma, Vladimír Křen, Kateřina Valentová

**Affiliations:** 1 Laboratory of Biotransformation, Institute of Microbiology, Czech Academy of Sciences, Vídeňská 1083, CZ-142 20 Prague, Czech Republic; fanny.langschwager@uni-rostock.de (F.L.); kalachova@gmail.com (L.J.-K.); alenka.petrickova@gmail.com (A.K.); baramiki@seznam.cz (B.M.); kuzma@biomed.cas.cz (M.K.); kren@biomed.cas.cz (V.K.); kata.valentova@email.cz (K.V.); 2 Institute of Chemistry, University of Rostock, Albert-Einstein-Str. 3a, GE-18059 Rostock, Germany

**Keywords:** isoquercitrin, quercetin, fatty acid, antioxidant activity, log *P*, Novozym 435, lipase, DPPH, lipoperoxidation

## Abstract

A series of isoquercitrin (quercetin-3-*O*-β-d-glucopyranoside) esters with mono- or dicarboxylic acids was designed to modulate hydro- and lipophilicity and biological properties. Esterification of isoquercitrin was accomplished by direct chemoenzymatic reaction using Novozym 435 (lipase from *Candida antarctica*), which accepted C_5_- to C_12_-dicarboxylic acids; the shorter ones, such as oxalic (C_2_), malonic (C_3_), succinic (C_4_) and maleic (C_4_) acids were not substrates of the lipase. Lipophilicity of monocarboxylic acid derivatives, measured as log *P*, increased with the chain length. Esters with glutaric and adipic acids exhibited hydrophilicity, and the dodecanedioic acid hemiester was more lipophilic. All derivatives were less able to reduce Folin–Ciocalteau reagent (FCR) and scavenge DPPH (1,1-diphenyl-2-picrylhydrazyl) than isoquercitrin; ABTS (2,2′-azinobis-(3-ethylbenzothiazoline-6-sulfonic acid)) radical-scavenging activity was comparable. Dodecanoate and palmitate were the least active in FCR and ABTS scavenging; dodecanoate and hemiglutarate were the strongest DPPH scavengers. In contrast, most derivatives were much better inhibitors of microsomal lipoperoxidation than isoquercitrin; butyrate and hexanoate were the most efficient. Anti-lipoperoxidant activity of monocarboxylic derivatives, except acetates, decreased with increasing aliphatic chain. The opposite trend was noted for dicarboxylic acid hemiesters, isoquercitrin hemidodecanedioate being the most active. Overall, IQ butyrate, hexanoate and hemidodecanedioate are the most promising candidates for further studies.

## 1. Introduction

**Figure 1:**
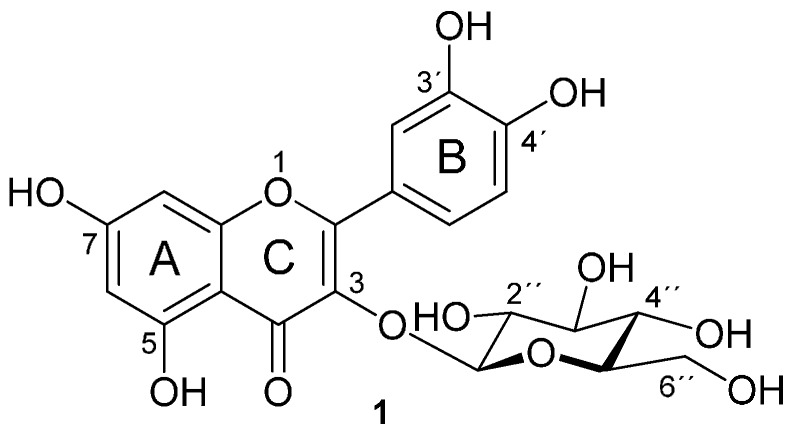
Isoquercitrin (**1**, quercetin-3-*O*-β-d-glucopyranoside).

Polyphenolic compounds are efficient antioxidants due to their ability to scavenge free radicals or chelate transition metals, which might initiate free radical formation [1,2]. Most polyphenols also modulate signaling pathways involved in, e.g., inflammation, carcinogenesis or cardiovascular disorders, and thus play an important role in the prevention of related chronic diseases [3]. Flavonoids that typically occur in fruits and vegetables are mostly derivatives of 1,4-benzopyrone. Among them, quercetin is one of the most abundant and important in terms of biological activity; it naturally occurs in various glycosylated forms in apples, onions, tea and red wine [4,5]. The most prominent quercetin glycosides are isoquercitrin (**1**, IQ, quercetin-3-*O*-β-d-glucopyranoside, Figure 1) and rutin (quercetin-3-*O*-[α-l-rhamnopyranosyl-(1→6)-β-d-glucopyranose]). While the biological activity of quercetin [6,7,8,9] and rutin [10] has been extensively studied, biological activities of isoquercitrin were evaluated to a much lesser extent [11].

The expanding array of biological activities reported as well as recently developed robust methods of biocatalytic production of pure isoquercitrin [12] attract the interest for isoquercitrin. Its health benefits seem to be very attractive for food, cosmetic and pharmaceutical industries [11]. However, such applications might be limited by its low solubility in organic (1.8, 30 and 14 g/L at 50 °C in acetonitrile, *tert*-amyl alcohol and acetone, respectively [13,14]) or aqueous (95 mg/L in water [15]) solvents. To overcome this problem, isoquercitrin modified by additional glucose units (enzymatically modified isoquercitrin, α-glucosyl isoquercitrin, enzymatically decomposed rutin) is being extensively studied [11,15]. Isoquercitrin derivatization by acylation with carboxylic acids seems to be another good option.

Conventional chemical acylations of flavonoids generally yield mixtures of products with various degree of esterification [16]. In contrast, chemo-enzymatic [17] and enzymatic syntheses [18,19] are very useful for acylation of these polyphenols. Enzymatic acylation of glycosylated flavonoids, catalyzed by *Candida antarctica* lipase B (CAL-B), is highly regioselective thanks to the nature of sugar moiety; it proceeds exclusively on its primary alcoholic group, and mild conditions of this reaction do not interfere with the structures of flavonoids [18].

Here, we present enzymatic synthesis of a series of isoquercitrin esters with mono- and dicarboxylic acids. Although isoquercitrin acetates [19,20], butyrate, hexanoate, octanoate, dodecanoate and palmitate [21] were prepared previously by slightly different techniques, they were not fully structurally characterized (missing nuclear magnetic resonance (NMR) data). Full spectroscopic characterization (crucial for unequivocal determination of the site of esterification), and, namely, enzymatic synthesis of the hemiesters are described here for the first time. To evaluate the pharmacological potential of these novel derivatives, their lipophilicity (log *P*), and their antiradical and anti-lipoperoxidant activities were determined.

## 2. Results and Discussion

### 2.1. The Preparation of Isoquercitrin Esters

**Scheme 1:**
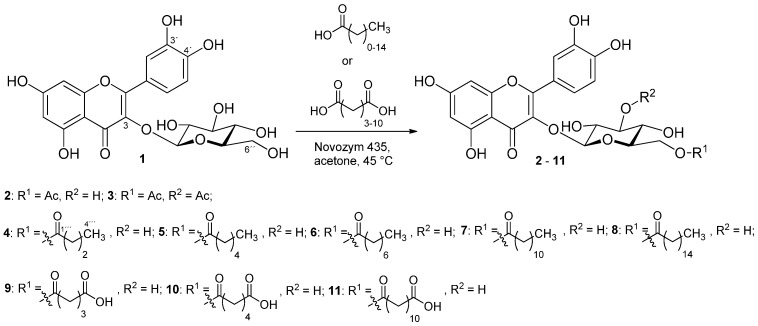
Preparation of isoquercitrin esters **2**–**11**.

Regioselectivity of the substitution of flavonoids is essential for successful preparation of their derivatives. An enzymatic approach overcomes protecting and deprotecting strategies, which are inevitable during chemical synthesis. Here, selective acylation of primary hydroxyl groups was accomplished by Novozym 435^®^—(CAL-B immobilized on an acrylic resin; (Novo-Nordisk, Copenhagen, Denmark)), taking advantage of its straightforward handling. Enzymatic reactions were performed with IQ and mono- or dicarboxylic aliphatic acids in ratios 1:10 or 1:20 in dry acetone (Scheme 1). Molecular sieves were instrumental for scavenging water generated during esterification reaction.

Structures of prepared derivatives were confirmed by NMR (Tables S1–S5 in Supplementary Materials). The ^1^H NMR signals were assigned by gCOSY (correlation spectroscopy) experiment, which allowed identification of particular spin systems. The compounds under study contain several spin systems, namely an ABC spin system of 1’,3’,4’-trisubstituted benzene ring, an AB spin system of 1,2,3,5-tetrasubstituted benzene ring, a spin system of –(CH)_5_–CH_2_– type belonging to a glucoside moiety, and an aliphatic spin system of –(CH_2_)*n*– linker (*n* = 3, 4, or 10). The assignment of protons was transferred to carbons by ^1^H-^13^C gHSQC (heteronuclear single-quantum correlation spectroscopy). The ^1^H-^13^C gHMBC (heteronuclear multiple-bond correlation spectroscopy) spectrum was used to assign the quaternary carbons and to put together the above-mentioned spin systems. The chemical shifts and HMBC couplings are consistent with the isoquercitrin moiety substituted at C-6″. The bond between the isoquercitrin moiety and the –(CH_2_)*n*– chain of dicarboxylic acid was approved by the HMBC correlation between C-1″′ and H-6″.

#### 2.1.1. Synthesis of Esters of Isoquercitrin and Monocarboxylic Aliphatic Acids (**2**–**8**)

Isoquercitrin acetylation was previously accomplished with two lipases; however, the reaction lacked regioselectivity [19]. CAL-B gave as the final product isoquercitrin 2”,3”,6”-triacetate while isoquercitrin 4’,6”-diacetate was formed in the reaction with *Pseudomonas cepacia* lipase [20]. We have isolated IQ 6”-acetate (**2**) and IQ 3”,6”-diacetate (**3**) in the yields 37% and 38%, respectively. Monoacetate **2** was formed as the first product after 2 h, and diacetate **3** was isolated after 24 h.

We have also prepared a panel of IQ derivatives substituted at C-6” OH (butyrate (**4**), hexanoate (**5**), octanoate (**6**), dodecanoate (**7**) and palmitate (**8**)) by direct lipase-mediated esterification from respective carboxylic acids in acetone with the yields 10%–33%. These compounds were previously prepared by Novozym 435^®^ catalyzed transesterification from respective ethyl esters in 2-methyl-2-butanol at 65 °C for 72 h [21]. Unfortunately, those products were characterized only by HPLC and LC-MS; NMR data were, however, provided only for IQ 6”-butyrate [21]. Due to the polyolic nature of the acceptor, mass spectrometry (MS) data are absolutely not sufficient for the structure determination. In contrast, our procedure is shorter (24 h), under milder conditions (45 °C), and we provide here complete structural characterization of the products including ESI-MS, ^1^H (600.23 MHz) and ^13^C (150.93 MHz) NMR (see the Experimental part and Supplementary Materials).

#### 2.1.2. Synthesis of Esters of Isoquercitrin with Aliphatic Dicarboxylic Acids (**9**–**11**)

The conversion of dicarboxylic acids was limited and strictly dependent on the chain length of the respective acid. Shorter dicarboxylic acids such as oxalic (C_2_), malonic (C_3_), succinic (C_4_) and maleic (C_4_) were not accepted by the lipase, while the enzyme has accepted C_5_- to C_12_-dicarboxylic acids yielding IQ hemiglutarate (C_5_, **9**), IQ hemiadipate (C_6_, **10**) and IQ hemidodecandioate (**11**, Scheme 1). This is in accordance with a previous report on PPL (porcine pancreatic lipases) catalyzed esterification of butyl α-d-glucopyranoside by succinic, adipic (C_6_) and hexadecanedioic acid, which yielded only 6”-*O*-hexadecanedioyl derivative [22]. In addition, CAL-B was previously shown to display considerably higher affinity for glutaric (C_5_) and adipic than for succinic and pimelic (C_7_) acids [23]. We can thus conclude that short-chain dicarboxylic acids (C_2_–C_4_) are not accepted by the lipase(s), probably due to their rather high polarity (two carboxyl groups *vs*. very short aliphatic chains). With the growing length of the dicarboxylic acid ≥C_5_, the lipophilicity of the acid becomes sufficient to be recognized by the lipase as the substrate.

To obtain the desired hemiesters (IQ hemioxalate, hemimalonate, hemisuccinate and hemimaleinate), several other possible chemoenzymatic preparation methods were tested. Previous studies [22,24] used as substrates for similar reactions (esterification of butyl α-d-glucopyranoside or polyhydroxylated steroids) succinic acid anhydride. Therefore, we have tested succinic anhydride as an acylating agent to prepare IQ hemisuccinate. Three products were identified as isoquercitrin mono-, di- and tri-succinylated derivatives by HPLC/MS (Figures S88–S92 in Supplementary Materials) in the presence of Novozym 435^®^, but also in its absence. This anhydride thus reacts spontaneously, the (enzymatic) product formation in the previous [22,24] studies being probably artifacts. We have determined that tentative structures of the products (LC-MS, Figures S90–S92 in Supplementary Materials) are a series of IQ succinic hemiesters, but the (regio)selectivity of this non enzymatic reaction is poor and thus not worth being studied further.

We have also tested the possible use of activated esters of dicarboxylic acids for lipase-mediated esterification, which can be used as acyl donors for the production of bifunctionalized compounds [25]. We have prepared divinyl esters of dicarboxylic acids (glutaric, adipic, dodecandioic) [26], which produced IQ mixed diesters vinylated at the distal end of the aliphatic chain. However, removal of the vinyl ester in acidic or basic conditions was not successful, and isoquercitrin esters were hydrolyzed. In contrast to our expectations and previous observation with Novozym 435^®^ catalyzed esterification of silybin with dicarboxylic acids [27], no dimeric byproducts were observed during the esterification of isoquercitrin reported here.

### 2.2. Partition Coefficient

The hydrophilicity/lipophilicity of a compound is often crucial for assessing its bioavailability. Flavonoids are generally poorly soluble in water; aqueous solubility increases at alkaline pH as phenols are weak acids. Using the high-purity isoquercitrin (96.5%) obtained by our enzymatic procedure [12], we have determined its solubility in water (pH = 6.5) to be 360 mg/L (compared to 95 mg/L, pH not given, ref. [15]).

Partition coefficient in octan-1-ol/phosphate buffer (pH 7.4) was measured for all prepared compounds as a basic empirical determination of hydro- or lipophilicity and expressed as log *P*. For isoquercitrin, quercetin and rutin, the values found (0.18, 1.94 and −0.41, respectively) were in good agreement with previously published data [28]. As expected, compounds **2**–**8** were found to be more lipophilic than isoquercitrin, and their lipophilicity increased with increasing carbon chain length (Table 1). For compounds **6**, **7** and **8**, log *P* could not be calculated due to unmeasurable content of the solute in the aqueous phase. Introduction of a second acetyl group into the molecule of IQ acetate effectively increased the lipophilicity of compound **3** in comparison with **2**. In contrast, hemiesters of isoquercitrin with glutaric (**9**) or adipic (**10**) acids exhibited high hydrophilicity, and their log *P* values were lower compared with isoquercitrin and rutin. Hydrophilic properties were thus efficiently improved by free carboxyl moiety introduced into the molecules. In the case of IQ hemidodecanedioate, the longer aliphatic chain (C_12_) led to more lipophilic character of the compound **11** despite the free carboxyl in the molecule.

### 2.3. Antioxidant Activity of Isoquercitrin Derivatives

To evaluate how isoquercitrin esterification affects its antioxidant activity, several experimental models, which differ in their mechanisms, solvents and pH [29] were used, namely measuring Folin–Ciocalteu reagent (FCR) reduction; 2,2-diphenyl-1-picrylhydrazyl (DPPH) radical scavenging; ABTS (2,2′-azinobis-(3-ethylbenzothiazoline-6-sulfonic acid)) cation radical scavenging; and the inhibition of lipoperoxidation (Lpx) in rat liver microsomal membranes oxidatively damaged by *tert*-butyl hydroperoxide (tBH, Table 1). Folin–Ciocalteu assay, commonly known as the total phenols assay, determines a sample reducing capacity. FCR contains heteropolyphosphotunstate−molybdates, which are reduced in aqueous milieu at pH ≈ 10 to blue species, presumably (PMoW_11_O_40_)^4−^ [29]. In FCR assay, the most active compound was quercetin (2.80 gallic acid equivalents, GAE) followed by rutin > IQ (**1**, 1.84 GAE) ≈ IQ butyrate (**4**) ≈ IQ hemiglutarate (**9**) > IQ acetate (**2**) ≈ IQ diacetate (**3**) ≈ IQ hexanoate (**5**) ≈ IQ octanoate (**6**) ≈ IQ hemiadipate (**10**) ≈ IQ hemidodecanedioate (**11**, 1.58 GAE) > IQ palmitate (**8**, 0.76 GAE) ≈ IQ dodecanoate (**7**, 0.63 GAE, Table 1). For most of the esters, the activity was comparable with or only slightly inferior to that of IQ. The most important loss of activity was observed in the case of IQ dodecanoate (**7**) and IQ palmitate (**8**). This is probably related to high hydrophobicity of these derivatives.

The 2,2-Diphenyl-1-picrylhydrazyl (DPPH) radical is a stable organic nitrogen radical with an absorption maximum at 515 nm in methanol. When reduced, the solution decolorizes in proportion to the strength and concentration of the antiradical compound tested [29]. The results of this test are usually expressed as the concentration of the compound that reduces 50% of the radical (IC_50_, Table 1). Compared to IQ (**1**), IQ dodecanoate (**7**) and IQ hemiglutarate (**9**) had comparable activity in this test, and all other derivatives were less active (higher IC_50_): IQ (**1**, 1.40 µM) ≈ IQ dodecanoate (**7**) ≈ IQ hemiglutarate (**9**) > IQ diacetate (**3**) ≈ IQ octanoate (**6**) ≈ IQ palmitate (**8**) ≈ IQ hemiadipate (**10**) > IQ acetate (**2**) ≈ IQ butyrate (**4**) > IQ hexanoate (**5**) ≈ IQ hemidodecanedioate (**11**) > quercetin > rutin (3.66 µM). The results for monocarboxylic acid esters are consistent with those reported previously [21,30]. However, no clear relationship between the carboxylic chain length and DPPH radical scavenging activity could be established.

Determination of ABTS scavenging activity involves an electron-transfer reaction [29]. In this assay, performed in an aqueous buffer at pH 5, none of the derivatives were superior to isoquercitrin, and their activity decreased in the order: IQ (**1**, 1.97 TE) ≈ IQ acetate (**2**) ≈ IQ butyrate (**4**) ≈ IQ hexanoate (**5**) ≈ IQ hemiglutarate (**9**) ≈ IQ hemiadipate (**10**) ≈ IQ hemidodecanedioate (**11**) ≈ quercetin > rutin > IQ diacetate (**3**) > IQ octanoate (**6**) > IQ dodecanoate (**7**) ≈ IQ palmitate (**8**, 1.0 TE). IQ esters of monocarboxylic acids thus followed the same trend as in the previous study [21], longer chain derivatives being less active. In contrast, more hydrophilic hemiesters had comparable activity to the parent compound IQ.

Lipid peroxidation is a type of oxidative degradation of lipids caused by free radicals in cell membranes, resulting in cell damage. The most notable initiators of this chain reaction in living cells are reactive oxygen species (ROS), such as OH**·** and HO_2_**·**, which combine with a hydrogen atom to make water and a fatty acid radical. We used *tert*-butyl hydroperoxide, a relatively stable organic peroxide, as initiator of peroxidation of rat liver microsomal membranes. The inhibition of this reaction depends not only on the free radical scavenging capacity, but also on the capacity of these compounds to interact with and penetrate lipid bilayers [31]. Isoquercitrin derivatives differed considerably in their capacity to inhibit the peroxidation of microsomal membranes. In the series of monocarboxylic acid derivatives, IQ acetate (**2**, C_2_, IC_50_ = 42 µM) was 23× more active than IQ (**1**, 972 µM) and, with the exception of IQ diacetate, the lipid peroxidation inhibition capacity slightly increased with increasing carboxylic chain length up to IQ hexanoate (**5**, C_6_, IC_50_ = 20 µM). In the longer chain derivatives, the anti-lipoperoxidant activity decreased gradually with increasing chain length, and IQ dodecanoate (**7**, C_12_, IC_50_ = 432 µM) was only twice as active as IQ. Accordingly, dodecanoyl esters of chrysoeriol glucopyranosides were previously shown to possess increased capacity to inhibit low density lipoprotein (LDL) oxidation induced by Cu^2+^ [32]. IQ palmitate (**8**, C_16_) had even slightly lower activity than IQ with IC_50_ = 1091 µM. A similar trend, e.g., improving the activity in medium chain derivatives with subsequent drop for longer chain esters, was reported for anti-proliferative activity of IQ monocarboxylic acid esters on the growth of tumoral Caco2 cells [21], and also for the inhibition of tyrosinase activity [30]. The chain length of the IQ hemiesters had also influenced the anti-lipoperoxidant activity, but here, with similar chain lengths, the trend was the opposite, with IQ hemiglutarate (**9**, 1341 µM) being less active than IQ. Anti-lipoperoxidant activity then increased considerably for IQ hemiadipate (**10**, 250 µM), and IQ hemidodecanedioate (**11**, 33 µM) had capacity comparable with that of quercetin (30 µM, Table 1). These results are most probably influenced by the spatial arrangement of the more or less long aliphatic chain in the presence of the water-lipid interphase, especially its behavior in the lipid bilayer of the membrane.

Considering all assays together, we have successfully synthesized several isoquercitrin derivatives with increased lipophilicity and conserved or even improved antioxidant properties. The ability of the compounds **2**, **3**, **4**, **5** and **11** to pass through a lipophilic membrane increases significantly relative to IQ. Their ability to scavenge DPPH or ABTS radicals is not substantially altered and the ability to inhibit lipid peroxidation is even significantly improved.

## 3. Materials and Methods

### 3.1. Chemicals and Reagents

Isoquercitrin (purity 96%) was prepared by enzymatic deglycosylation of rutin using our procedure [12,33]. Lipase B from *Candida antarctica* immobilized on acrylic resin (Novozym 435) was purchased from Novo-Nordisk (Copenhagen, Denmark). Folin–Ciocalteau reagent was purchased from Merck (Prague, Czech Republic). In addition, DPPH radical, antioxidant assay kit (CS0790); pooled microsomes from male rat liver (M9066); Trolox and other chemicals were obtained from Sigma–Aldrich (Prague, Czech Republic).

### 3.2. Nuclear Magnetic Resonance (NMR) and Mass Spectrometry (MS) Methods

NMR spectra were recorded on a Bruker Avance III 700 MHz spectrometer (700.13 MHz for ^1^H, 176.05 MHz for ^13^C at 30 °C) and a Bruker Avance III 600 MHz spectrometer (600.23 MHz for ^1^H, 150.93 MHz for ^13^C at 30 °C, both from Bruker Daltonik, Bremen, Germany)) in DMSO-*d*_6_ (99.8% atom D, ARMAR Chemicals, Döttingen, Switzerland). The residual signal of the solvent was used as an internal standard (δ_H_ 2.500 ppm, δ_C_ 39.60 ppm). NMR experiments—^1^H NMR, ^13^C NMR, COSY, HSQC, HMBC and 1D TOCSY—were performed using the manufacturer’s software. ^1^H NMR and ^13^C NMR spectra were zero filled to four-fold data points and multiplied by a window function before Fourier transformation.

The two-parameter double-exponential Lorentz–Gauss function was applied for ^1^H to improve resolution, and line broadening (1 Hz) was applied to get a better ^13^C signal-to-noise ratio. Chemical shifts are given in δ-scale with digital resolution justifying the reported values to three (δ_H_) or two (δ_C_) decimal places.

Mass spectra were acquired using Shimadzu Prominence system (Shimadzu, Kyoto, Japan) equipped with an electrospray ion source. The samples were dissolved in acetonitrile and introduced into the mobile phase flow (acetonitrile, 0.4 mL/min).

### 3.3. HPLC Analysis

All analytical HPLC analyses were performed with the Shimadzu Prominence System (Shimadzu, Kyoto, Japan) consisting of a DGU-20A mobile phase degasser, two LC-20AD solvent delivery units, a SIL-20AC cooling auto sampler, a CTO-10AS column oven and SPD-M20A diode array detector. Chromatographic data were collected and processed using Shimadzu Solution software at a rate of 40 Hz and detector time constant of 0.025 s. The Chromolith Performance RP-18e monolithic column (100 × 3 mm i.d., Merck, Darmstadt, Germany) coupled with a guard column (5 × 4.6 mm; Merck, Darmstadt, Germany) was used. Mobile phase acetonitrile/water/formic acid (80/20/0.1, *v/v/v*, phase A) and acetonitrile/water/formic acid (5/95/0.1, *v*/*v*/*v*, phase B) were employed in the analyses; gradient: 0–10 min 7%–80% A; 10–12 min 80% A, 12–14 min 80%–7% A. The flow rate was 1.2 mL/min at 25 °C. The photodiode array (PDA) data were acquired in the 200–450 nm range, and 360 nm signals were extracted.

### 3.4. Measurement of Log P

The hydrophobicity of the prepared compounds was determined by the measurement of partition coefficient *P* in a mixture of two immiscible phases—octan-1-ol and 6.6 mM phosphate buffer pH 7.4 to simulate physiological conditions. Before the use, octan-1-ol was stirred with the buffer for 16 h at 25 °C to achieve saturation of both phases, which were then separated.

Stock solutions (0.2–0.5 mM) of tested compounds were prepared in octan-1-ol in the case of compounds **1**–**8** and quercetin and in the buffer for compounds **9**–**11** and rutin. Then, 150 µL of the stock solutions were mixed with 150 µL of the respective immiscible phase in microcentrifuge tubes (1.5 mL) and stirred (750 rpm) for 2 h at 25 °C in triplicates. Phases were separated and the solute concentration in each phase was determined in 96-well microtitration plates using Sunrise™ spectrophotometer (Schoeller Instruments, Prague, Czech Republic) at 400 nm. Log *P* was calculated as follows: log *P*_oct/buffer_ = log([A]_octan-1-ol_/[A]_buffer_).

### 3.5. Chemistry

#### 3.5.1. Synthesis of Isoquercitrin Esters **2**–**8**

Isoquercitrin (**1**, 300 mg, 0.65 mmol, 1 eq.) and respective aliphatic acid (6.46 mmol, 10 eq.) were dissolved in anhydrous acetone (15 mL). 400 mg of Novozym 435^®^ and 300 mg of molecular sieves Å4 were added to the solution. The reaction mixture was incubated at 45 °C, 220 rpm. Part of IQ remained undissolved, and it dissolved gradually in the course of the reaction. After 2 h (compound **2**) or 24 h (compounds **3**–**8**), the reaction was terminated by filtering off the enzyme and the solvent was evaporated under reduced pressure. The crude product was purified by silica gel flash chromatography (chloroform/methanol 95:5). 

((2*R*,3*S*,4*S*,5*R*,6*S*)-6-((2-(3,4-Dihydroxyphenyl)-5,7-dihydroxy-4-oxo-4*H*-chromen-3-yl)oxy)-3,4,5-trihydroxytetrahydro-2*H*-pyran-2-yl)methyl acetate (**2**, IQ acetate): Yellow solid (yield 37%, 123 mg, 0.024 mmol). For ^1^H and ^13^C NMR data, see Tables S1, S2 and Figures S2–S4 in the Supplementary Materials. MS-ESI *m*/*z*: [M − H]^−^ calcd. for C_23_H_22_O_13_ 505.1; found: 505.1.

((2*R*,3*S*,4*R*,5*R*,6*S*)-3-Acetoxy-6-((2-(3,4-dihydroxyphenyl)-5,7-dihydroxy-4-oxo-4*H*-chromen-3-yl)oxy)-4,5-dihydroxytetrahydro-2*H*-pyran-2-yl)methyl acetate (**3**, IQ diacetate): Yellow solid (yield 38%, 136 mg, 0.025 mmol). For ^1^H and ^13^C NMR data, see Tables S1, S2 and Figures S5–S7 in the Supplementary Materials. MS-ESI *m*/*z*: [M + H]^+^ calcd. for C_25_H_25_O_14_ 549.1; found: 549.2.

((2*R*,3*S*,4*S*,5*R*,6*S*)-6-((2-(3,4-Dihydroxyphenyl)-5,7-dihydroxy-4-oxo-4*H*-chromen-3-yl)oxy)-3,4,5-trihydroxytetrahydro-2*H*-pyran-2-yl)methyl butyrate (**4**, IQ butyrate): Yellow solid (yield 13%, 45 mg, 0.008 mmol). For ^1^H and ^13^C NMR data, see Tables S1, S3 and Figures S8–S10 in the Supplementary Materials. MS-ESI *m*/*z*: [M + H]^+^ calcd. for C_25_H_27_O_13_ 535.1; found: 535.2.

((2*R*,3*S*,4*S*,5*R*,6*S*)-6-((2-(3,4-Dihydroxyphenyl)-5,7-dihydroxy-4-oxo-4*H*-chromen-3-yl)oxy)-3,4,5-trihydroxytetrahydro-2*H*-pyran-2-yl)methyl hexanoate (**5**, IQ hexanoate): Yellow solid (yield 33%, 120 mg, 0.021 mmol). For ^1^H and ^13^C NMR data, see Tables S1, S3 and Figures S11–S13 in the Supplementary Materials. MS-ESI *m*/*z*: [M + H]^+^ calcd. for C_27_H_31_O_13_ 563.2; found: 563.2.

((2*R*,3*S*,4*S*,5*R*,6*S*)-6-((2-(3,4-Dihydroxyphenyl)-5,7-dihydroxy-4-oxo-4*H*-chromen-3-yl)oxy)-3,4,5-trihydroxytetrahydro-2*H*-pyran-2-yl)methyl octanoate (**6**, IQ octanoate): Yellow solid (yield 27%, 103 mg, 0.017 mmol). For ^1^H and ^13^C NMR data, see Tables S1, S3 and Figures S14–S16 in the Supplementary Materials. MS-ESI *m*/*z*: [M + H]^+^ calcd. for C_29_H_35_O_13_ 591.2; found: 591.3.

((2*R*,3*S*,4*S*,5*R*,6*S*)-6-((2-(3,4-Dihydroxyphenyl)-5,7-dihydroxy-4-oxo-4*H*-chromen-3-yl)oxy)-3,4,5-trihydroxytetrahydro-2*H*-pyran-2-yl)methyl dodecanoate (**7**, IQ dodecanoate): Yellow solid (yield 22%, 91 mg, 0.014 mmol). For ^1^H and ^13^C NMR data, see Tables S1, S3 and Figures S17–S19 in the Supplementary Materials. MS-ESI *m*/*z*: [M − H]^−^ calcd. for C_33_H_40_O_13_ 645.3; found: 645.0.

((2*R*,3*S*,4*S*,5*R*,6*S*)-6-((2-(3,4-Dihydroxyphenyl)-5,7-dihydroxy-4-oxo-4*H*-chromen-3-yl)oxy)-3,4,5-trihydroxytetrahydro-2*H*-pyran-2-yl)methyl palmitate (**8**, IQ palmitate): Yellow solid (yield 10%, 44 mg, 0.006 mmol). For ^1^H and ^13^C NMR data, see Tables S1, S3 and Figures S20–S22 in the Supplementary Materials. MS-ESI *m*/*z*: [M − H]^−^ calcd. for C_37_H_49_O_13_ 701.3; found: 701.2.

#### 3.5.2. General Procedure–Synthesis of Isoquercitrin Esters **9**–**11**

Isoquercitrin (**1**, 300 mg, 0.65 mmol, 1 eq.) and respective aliphatic dicarboxylic acids (12.92 mmol, 20 eq.) were dissolved in anhydrous acetone (20 mL). 400 mg of Novozym 435^®^ and 300 mg of molecular sieves Å4 were added to the solution. The reaction mixture was incubated at 45 °C, 220 rpm. After 72 h, the reaction was terminated by filtering off the enzyme and the solvent was evaporated under reduced pressure. The crude product was purified by silica gel flash chromatography (chloroform/methanol 95:5), then followed by gel filtration using LH-20 in methanol.

6-(((2*R*,3*S*,4*S*,5*R*,6*S*)-6-((2-(3,4-Dihydroxyphenyl)-5,7-dihydroxy-4-oxo-4*H*-chromen-3-yl)oxy)-3,4,5-trihydroxytetrahydro-2*H*-pyran-2-yl)methoxy)-6-oxopentanoic acid (**9**, IQ hemiglutarate): Yellow solid (yield 36%, 135 mg, 0.023 mmol). For ^1^H and ^13^C NMR data, see Tables S4, S5 and Figures S23–S41 in the Supplementary Materials. MS-ESI *m*/*z*: [M − H]^−^ calcd. for C_26_H_25_O_15_ 577.1; found: 577.1.

6-(((2*R*,3*S*,4*S*,5*R*,6*S*)-6-((2-(3,4-Dihydroxyphenyl)-5,7-dihydroxy-4-oxo-4*H*-chromen-3-yl)oxy)-3,4,5-trihydroxytetrahydro-2*H*-pyran-2-yl)methoxy)-6-oxohexanoic acid (**10**, IQ hemiadipate): Yellow solid (yield 38%, 146 mg, 0.025 mmol). For ^1^H and ^13^C NMR data, see Tables S4, S5 and Figures S42–S67 in the Supplementary Materials. MS-ESI *m*/*z*: [M − H]^−^ calcd. for C_27_H_28_O_15_ 591.1; found: 591.1.

12-(((2*R*,3*S*,4*S*,5*R*,6*S*)-6-((2-(3,4-Dihydroxyphenyl)-5,7-dihydroxy-4-oxo-4*H*-chromen-3-yl)oxy)-3,4,5-trihydroxytetrahydro-2*H*-pyran-2-yl)methoxy)-12-oxododecanoic acid (**11**, IQ hemidodecanedioate): Yellow solid (yield 23%, 102 mg, 0.015 mmol). For ^1^H and ^13^C NMR data, see Tables S4, S5 and Figures S68–S87 in the Supplementary Materials. MS-ESI *m*/*z*: [M − H]^−^ calcd. for C_33_H_40_O_15_ 675.2; found: 675.2.

### 3.6. Antioxidant Activity Measurement

#### 3.6.1. Folin–Ciocalteau Reduction (FCR) Assay

Reducing capacity was evaluated using Folin–Ciocalteau reagent [34] with minor modifications as described previously [35].

#### 3.6.2. DPPH Assay

Antiradical activity was evaluated spectrophotometrically as the ability of the substances to reduce the DPPH radical as described previously [36,37] with minor modifications [35]. Briefly, 20 µL of the tested substance in methanol (final concentration 0–25 µM) were mixed with 280 µL of a freshly prepared methanolic DPPH solution (final concentration 20 µM). After 30 min, the absorbance at 517 nm was read, the extent of inhibition was calculated and the IC_50_ values were determined from the inhibition curves.

#### 3.6.3. ABTS^·^ Scavenging

The capacity to scavenge the radical cation (ABTS^·^) was evaluated using the Antioxidant Assay Kit (CS0790, Sigma-Aldrich, Prague, Czech Republic) and expressed as Trolox equivalents (TE) from the Trolox calibration curve.

#### 3.6.4. Inhibition of Microsomal Lipid Peroxidation

Pooled microsomes from male rat livers (M9066, Sigma-Aldrich, Prague, Czech Republic) were washed five times using centrifugation and phosphate-buffered saline (PBS, 13,500 rpm, 5 min, 4 °C) to remove sucrose. In addition, 400 µL of the diluted microsomal suspension (0.625 mg protein/mL in PBS), the compounds tested in 50 µL of dimethyl sulfoxide (DMSO, final concentration 5–2000 µM) and 50 µL of *tert*-butylhydroperoxide in PBS (final concentration 1 mM) were mixed and incubated at 37 °C for 30 min. Determination of lipid peroxidation products as thiobarbituric acid reactive substances (TBARS) and calculation of the IC_50_ values were performed as described previously [35].

#### 3.6.5. Statistical Analysis

All data were analyzed with one-way ANOVA, Scheffé and Least Square Difference tests for *post hoc* comparisons among pairs of means using the statistical package Statext ver. 2.1 (Wayne, NJ, USA). Differences were considered statistically significant when *p* < 0.05.

## 4. Conclusions

Isoquercitrin derivatives of mono- or dicarboxylic acids were prepared by lipase-mediated one-step synthesis. IQ was substituted at 6”-OH by acetate or by C_4_- to C_16_-aliphatic acids. The conversion rates with dicarboxylic acids were limited and depended on the chain length. Shorter dicarboxylic acids (C_2_- to C_4_) were not substrates for the lipase and did not react at all, while the enzyme has accepted C_5_- to C_12_-dicarboxylic acid. Hydrophilic or lipophilic character of all prepared compounds was determined by log *P*. Hydrophilic properties were enhanced by the introduction of free carboxylic group to the molecules, which was evident with hemiglutaric and hemiadipic IQ esters. On the other hand, the IQ esters with longer aliphatic chain exhibited more lipophilic properties. DPPH scavenging, Folin–Ciocalteau reduction assay, ABTS scavenging and lipoperoxidation were measured for all prepared compounds. In DPPH assay and Folin–Ciocalteau reduction assay, none of the derivatives were superior to isoquercitrin or rutin. Esters of monocarboxylic acids IQ butyrate and IQ hexanoate were the most powerful inhibitors of lipid peroxidation, while weak activity was observed for esters with longer aliphatic chains. In the case of hemiesters, the anti-lipoperoxidant activity was improved for IQ hemidodecanedioate; this means that the length of aliphatic chain was determined to be a crucial factor for the interaction with lipid bilayers.

## Figures and Tables

**Table 1:** Log *P* values, radical scavenging and anti-lipoperoxidant activity of isoquercitrin, compounds **2**–**11** and standards.

Compounds		Partition Coefficient	log *P*	FCR (GAE)	DPPH (IC_50_; µM)	ABTS (TE)	Lpx (IC_50_; µM)
1	Isoquercitrin	1.52 ± 0.01	0.18	1.84 ± 0.04 ^a^	1.40 ± 0.06 ^d^	1.97 ± 0.06 ^i^	972 ± 11
2	IQ acetate	13.76 ± 0.01	1.14	1.63 ± 0.07 ^b^	2.51 ± 0.10 ^e^	2.04 ± 0.07 ^i^	42.0 ± 2.1 ^k^
3	IQ diacetate	38.99± 0.01	1.59	1.67 ± 0.11 ^b^	2.15 ± 0.04 ^f^	1.56 ± 0.04	433 ± 19
4	IQ butyrate	90.64 ± 0.03	1.96	1.72 ± 0.03 ^a^	2.51 ± 0.17 ^e^	2.16 ± 0.08 ^i^	24.1 ± 0.8 ^l^
5	IQ hexanoate	126.43 ± 0.01	2.10	1.48 ± 0.05 ^b^	3.07 ± 0.10 ^g^	2.11 ± 0.09 ^i^	19.7 ± 0.2 ^l^
6	IQ octanoate	n.d.	n.d.	1.47 ± 0.10 ^b^	2.23 ± 0.08 ^e,f^	1.28 ± 0.07	186 ± 3 ^m^
7	IQ dodecanoate	n.d.	n.d.	0.63 ± 0.07 ^c^	1.58 ± 0.05 ^d^	1.06 ± 0.09 ^j^	432 ± 24
8	IQ palmitate	n.d.	n.d.	0.76 ± 0.03 ^c^	2.23 ± 0.03 ^e,f^	1.00 ± 0.06 ^j^	1091 ± 36
9	IQ hemiglutarate	0.07 ± 0.01	−1.15	1.84 ± 0.05 ^a^	1.77 ± 0.06 ^d^	2.13 ± 0.08 ^i^	1341 ± 54
10	IQ hemiadipate	0.10 ± 0.01	−1.00	1.46 ± 0.14 ^b^	2.19 ± 0.12 ^e,f^	2.12 ± 0.10 ^i^	250 ± 19
11	IQ hemidodecanedioate	3.00 ± 0.03	0.48	1.58 ± 0.05 ^b^	2.97 ± 0.22 ^g^	2.16 ± 0.08 ^i^	32.5 ± 1.6 ^k^
	Quercetin	86.27 ± 0.02	1.94	2.80 ± 0.16	3.78 ± 0.15 ^h^	2.14 ± 0.10 ^i^	29.8 ± 0.6 ^k^
	Rutin	0.41 ± 0.01	−0.39	2.19 ± 0.10	3.66 ± 0.39 ^h^	1.79 ± 0.09	202 ± 8 ^m^

n.d.—Not determined, concentration in the buffer was below the detection limit. The results of partition coefficient are presented as mean ± SD performed in triplicate. Data of FCR, DPPH, ABTS and Lpx are presented as means ± SE from at least three independent experiments performed in triplicate. The values marked with the same letter are not significantly different. FCR: Folin–Ciocalteau reduction assay; GAE: Gallic acid equivalents; DPPH: 1,1-Diphenyl-2-picrylhydrazyl scavenging; ABTS: 2,2′-Azinobis-(3-ethylbenzothiazoline-6-sulfonic acid) radical cation scavenging; TE: Trolox-equivalents; Lpx: Lipid peroxidation; IC_50_: The concentration of the tested compound that inhibited the reaction by 50%.

## Supplementary Materials

Supplementary materials can be found at http://www.mdpi.com/1422-0067/17/6/899/s1.

## Abbreviations

ABTS	2,2′-azinobis-(3-ethylbenzothiazoline-6-sulfonic acid)
CAL-B	Lipase B from *Candida antarctica*
COSY	Correlation spectroscopy
DMSO	Dimethyl sulfoxide
DPPH	1,1-Diphenyl-2-picrylhydrazyl radical
FCR	Folin–Ciocalteau reduction assay
GAE	Gallic acid equivalents
HMBC	Heteronuclear multiple-bond correlation spectroscopy
HSQC	Heteronuclear single-quantum correlation spectroscopy
IC_50_	The concentration of the tested compound that inhibited the reaction by 50%
IQ	Isoquercitrin
LDL	Low-density lipoprotein
Lpx	Lipid peroxidation
PBS	Phosphate buffer saline
PDA	Photodiode array
TBARS	Thiobarbituric acid reactive substances
TE	Trolox-equivalent
