# Supplementary material for: Isoquercitrin Esters with Mono- or Dicarboxylic Acids: Enzymatic Preparation and Properties
Source: Int J Mol Sci. 2016 Jun 7;17(6):899. doi: 10.3390/ijms17060899 (PMC4926433; doi:10.3390/ijms17060899)
Supplement: Supplementary file 1 [file ijms-17-00899-s001.pdf]

Supplementary Materials: Isoquercitrin Esters with Mono- or Dicarboxylic Acids: Enzymatic Preparation and Properties

Eva Vavříková, Fanny Langschwager, Lubica Jezova-Kalachova, Alena Křenková, Barbora Mikulová, Marek Kuzma, Vladimír Křen and Kateřina Valentová

Content **Page**

Figure S1 Structures of compounds **2–11** S3
Table S1 ^{13}C NMR data of prepared compounds **2–8** S4
Table S2 ^{1}H NMR data of prepared compounds **2** and **3** S6
Table S3 ^{1}H NMR data of prepared compounds **4–8** S7
Table S4 ^{13}C NMR data of prepared compounds **9–11** S9
Table S5 ^{1}H NMR data of prepared compounds **9–11** S10
Figure S2 ^{13}C NMR spectrum of compound **2** S11
Figure S3 ^{1}H NMR spectrum of compound **2** S12
Figure S4 HPLC chromatogram of compound **2** S13
Figure S5 ^{13}C NMR spectrum of compound **3** S14
Figure S6 ^{1}H NMR spectrum of compound **3** S15
Figure S7 HPLC chromatogram of compound **3** S16
Figure S8 ^{13}C NMR spectrum of compound **4** S17
Figure S9 ^{1}H NMR spectrum of compound **4** S18
Figure S10 HPLC chromatogram of compound **4** S19
Figure S11 ^{13}C NMR spectrum of compound **5** S20
Figure S12 ^{1}H NMR spectrum of compound **5** S21
Figure S13 HPLC chromatogram of compound **5** S22
Figure S14 ^{13}C NMR spectrum of compound **6** S23
Figure S15 ^{1}H NMR spectrum of compound **6** S24
Figure S16 HPLC chromatogram of compound **6** S25
Figure S17 ^{13}C NMR spectrum of compound **7** S26
Figure S18 ^{1}H NMR spectrum of compound **7** S27
Figure S19 HPLC chromatogram of compound **7** S28
Figure S20 ^{13}C NMR spectrum of compound **8** S29
Figure S21 ^{1}H NMR spectrum of compound **8** S30
Figure S22 HPLC chromatogram of compound **8** S31
Figure S23 ^{13}C NMR spectrum of compound **9** S32
Figure S24 ^{1}H NMR spectrum of compound **9** S33
Figure S25–27 Details of ^{1}H NMR spectrum of compound **9** S34–S36
Figure S28 HSQC NMR spectrum of compound **9** S37
Figure S29–31 Details of HSQC NMR spectrum of compound **9** S38–S40
Figure S32 HMBC NMR spectrum of compound **9** S41
Figure S33–36 Details of HMBC NMR spectrum of compound **9** S42–S45
Figure S37 COSY NMR spectrum of compound **9** S46
Figure S38–40 Details of COSY NMR spectrum of compound **9** S47–S49
Figure S41 HPLC chromatogram of compound **9** S50
Figure S42 ^{13}C NMR spectrum of compound **10** S51
Figure S43–45 Details of ^{13}C NMR spectrum of compound **10** S52–S54
Figure S46 ^{1}H NMR spectrum of compound **10** S55
Figure S47–49 Details of ^{1}H NMR spectrum of compound **10** S56–S58
Figure S50 HSQC NMR spectrum of compound **10** S59
Figure S51–54 Details of HSQC NMR spectrum of compound **10** S60–S63

Figure S55 HMBC NMR spectrum of compound **10** ... S64
Figure S56–62 Details of HMBC NMR spectrum of compound **10** ... S65–S71
Figure S63 COSY NMR spectrum of compound **10** ... S72
Figure S64–66 Details of COSY NMR spectrum of compound **10** ... S73–S75
Figure S67 HPLC chromatogram of compound **10** ... S76
Figure S68 ^{1}H NMR spectrum of compound **11** ... S77
Figure S69 ^{1}H NMR spectrum WET of compound **11** ... S78
Figure S70–72 Details of ^{1}H NMR spectrum WET of compound **11** ... S79–S81
Figure S73 HSQC NMR spectrum of compound **11** ... S82
Figure S74–76 Details of HSQC NMR spectrum of compound **11** ... S83–S85
Figure S77 HMBC NMR spectrum of compound **11** ... S86
Figure S78–82 Details of HMBC NMR spectrum of compound **11** ... S87–S91
Figure S83 COSY NMR spectrum of compound **11** ... S92
Figure S84–86 Details of COSY NMR spectrum of compound **11** ... S93–S95
Figure S87 HPLC chromatogram of compound **11** ... S96
Figure S88 HPLC chromatogram of the reaction mixture of isoquercitrin, succinic anhydride in the presence of Novozym 435® ... S97
Figure S89 HPLC chromatogram of the reaction mixture of isoquercitrin, succinic anhydride in the absence of Novozym 435® ... S98
Figure S90 HPLC-MS (−) chromatogram of the monosuccinate of isoquercitrin ... S99
Figure S91 HPLC-MS (−) chromatogram of the disuccinate of isoquercitrin ... S100
Figure S92 HPLC-MS (−) chromatogram of the trisuccinate of isoquercitrin ... S101

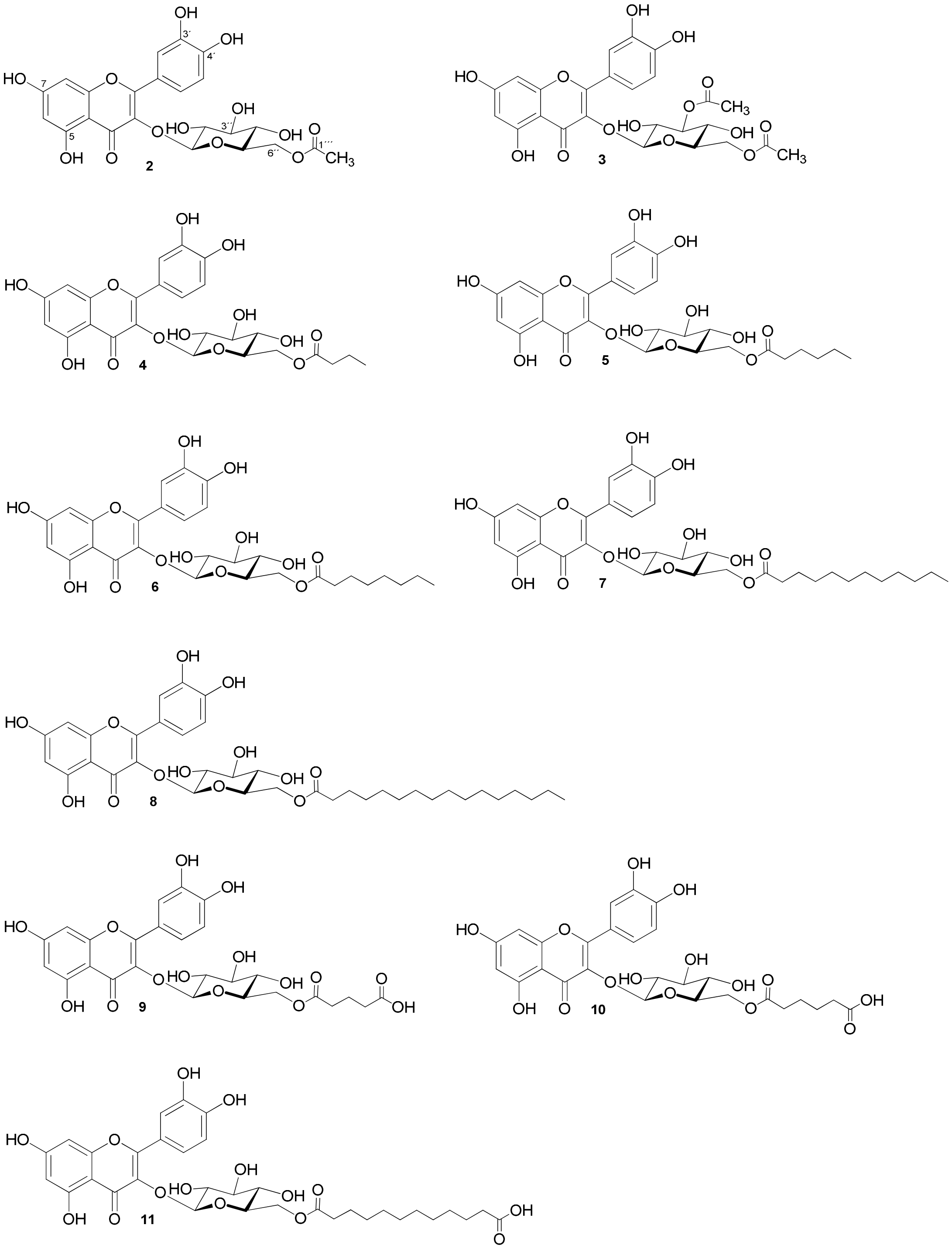

Figure S1. Structures of compounds **2–11**.

Table S1. ^{13}C NMR data of prepared compounds **2–8** (DMSO-d_6, 30 °C).

**Comp.**	**2**		**3**		**4**		**5**		**6**		**7**		**8**	
**Atom Number**	**δ C**	**m C**	**δ C**	**m C**	**δ C**	**m C**	**δ C**	**m C**	**δ C**	**m C**	**δ C**	**m C**	**δ C**	**m C**
**2**	156.52	s	156.68	s	156.38	s	156.40	s	156.41	s	156.41	s	156.40	s
**3**	133.18	s	132.99	s	132.99	s	133.03	s	133.03	s	133.05	s	133.05	s
**4**	177.38	s	177.27	s	177.39	s	177.39	s	177.39	s	177.39	s	177.39	s
**4a**	103.86	s	103.90	s	103.85	s	103.86	s	103.86	s	103.87	s	103.85	s
**5**	161.27	s	161.27	s	161.25	s	161.30	s	161.30	s	161.30	s	161.30	s
**6**	98.73	d	98.76	d	98.72	d	98.68	d	98.68	d	98.68	d	98.69	d
**7**	164.33	s	164.27	s	164.31	s	164.25	s	164.21	s	164.42	s	164.28	s
**8**	93.57	d	93.61	d	93.53	d	93.48	d	93.46	d	93.46	d	93.47	d
**8a**	156.38	s	156.38	s	156.32	s	156.35	s	156.34	s	156.34	s	156.34	s
**1′**	148.56	s	148.64	s	148.55	s	148.52	s	148.51	s	148.52	s	148.53	s
**2′**	144.88	s	144.85	s	144.90	s	144.87	s	144.86	s	144.86	s	144.87	s
**3′**	116.18	d	116.25	d	116.16	d	116.12	d	116.13	d	116.15	d	116.15	d
**4′**	121.08	s	120.96	s	121.07	s	121.10	s	121.09	s	121.09	s	121.08	s
**5′**	121.55	d	121.49	d	121.46	d	121.50	d	121.49	d	121.47	d	121.46	d
**6′**	115.16	d	115.22	d	115.25	d	115.11	d	115.13	d	115.13	d	115.14	d
**1″**	101.03	d	100.73	d	100.60	d	100.66	d	100.68	d	100.72	d	100.72	d
**2″**	74.01	d	71.82	d	73.95	d	73.96	d	73.96	d	73.96	d	73.96	d
**3″**	76.30	d	77.19	d	76.36	d	76.35	d	76.35	d	76.36	d	76.36	d
**4″**	69.83	d	67.65	d	70.15	d	70.13	d	70.14	d	70.14	d	70.14	d
**5″**	74.01	d	73.66	d	74.17	d	74.18	d	74.17	d	74.19	d	74.19	d
**6″**	62.81	t	62.34	t	62.94	t	62.92	t	62.93	t	62.95	t	62.95	t
**5-OH**	-	-	-	-	-	-	-	-	-	-	-	-	-	-
**7-OH**	-	-	-	-	-	-	-	-	-	-	-	-	-	-
**1′-OH**	-	-	-	-	-	-	-	-	-	-	-	-	-	-
**2′-OH**	-	-	-	-	-	-	-	-	-	-	-	-	-	-
**2″-OH**	-	-	-	-	-	-	-	-	-	-	-	-	-	-
**3″-OH**	-	-	-	-	-	-	-	-	-	-	-	-	-	-
**4″-OH**	-	-	-	-	-	-	-	-	-	-	-	-	-	-
**3″-CO**	-	-	169.70	s	-	-	-	-	-	-	-	-	-	-
**3″-Ac**	-	-	21.11	q	-	-	-	-	-	-	-	-	-	-
**6″-CO**	-	-	169.88	s	-	-	-	-	-	-	-	-	-	-
**6″-Ac**	-	-	20.13	q	-	-	-	-	-	-	-	-	-	-

Table S1. *Cont.*

**Comp.**	**2**		**3**		**4**		**5**		**6**		**7**		**8**	
**Atom Number**	**δ C**	**m C**	**δ C**	**m C**	**δ C**	**m C**	**δ C**	**m C**	**δ C**	**m C**	**δ C**	**m C**	**δ C**	**m C**
**1′′′**	169.88	s	-	-	172.29	s	172.42	s	172.43	s	172.44	s	172.45	s
**2′′′**	20.12	q	-	-	35.25	t	33.19	t	33.24	t	33.24	t	33.24	t
**3′′′**	-	-	-	-	17.81	t	23.92	t	24.24	t	24.23	t	24.24	t
**4′′′**	-	-	-	-	13.19	q	30.51	t	28.27	t	28.31	t	28.32	t
**5′′′**	-	-	-	-	-	-	21.61	t	28.18	t	28.52	t	28.53	t
**6′′′**	-	-	-	-	-	-	13.75	d	31.09	t	28.73	t	28.74	t
**7′′′**	-	-	-	-	-	-	-	-	22.03	t	28.86	t	28.87	t
**8′′′**	-	-	-	-	-	-	-	-	13.95	q	29.00	t	29.00	t
**9′′′**	-	-	-	-	-	-	-	-	-	-	29.02	t	29.05	t
**10′′′**	-	-	-	-	-	-	-	-	-	-	31.33	t	29.06	t
**11′′′**	-	-	-	-	-	-	-	-	-	-	22.12	t		
**12′′′**	-	-	-	-	-	-	-	-	-	-	13.98	q	29.09 [3C]	t
**13′′′**	-	-	-	-	-	-	-	-	-	-	-	-		
**14′′′**	-	-	-	-	-	-	-	-	-	-	-	-	31.33	t
**15′′′**	-	-	-	-	-	-	-	-	-	-	-	-	22.12	t
**16′′′**	-	-	-	-	-	-	-	-	-	-	-	-	13.97	q

[3C] … three overlapped carbon signals.

Table S2. ^{1}H NMR data of prepared compounds **2** and **3** (DMSO-d_6, 30 °C).

Comp.	2				3			
**Atom Number**	**δ H**	**n H**	**m**	**J [Hz]**	**δ H**	**n H**	**m**	**J [Hz]**
**2**	-	-	-	-	-	-	-	-
**3**	-	-	-	-	-	-	-	-
**4**	-	-	-	-	-	-	-	-
**4a**	-	-	-	-	-	-	-	-
**5**	-	-	-	-	-	-	-	-
**6**	6.201	1	d	2.1	6.210	1	d	2.0
**7**	-	-	-	-	-	-	-	-
**8**	6.409	1	d	2.1	6.414	1	d	2.0
**8a**	-	-	-	-	-	-	-	-
**1′**	-	-	-	-	-	-	-	-
**2′**	-	-	-	-	-	-	-	-
**3′**	7.528	1	d	2.2	7.543	1	d	2.2
**4′**	-	-	-	-	-	-	-	-
**5′**	7.531	1	dd	9.0, 2.2	7.509	1	dd	2.2, 8.4
**6′**	6.832	1	d	9.0	6.841	1	d	8.4
**1″**	5.372	1	d	7.5	5.482	1	d	7.7
**2″**	3.27 H	1	m	-	3.47 *	1	ddd	7.7, 4.9, 9.3
**3″**	3.25 H	1	m	-	4.849	1	dd	9.3 , 9.3
**4″**	3.147	1	t	9.0	3.389	1	ddd	6.2, 9.3, 9.5
**5″**	3.30 H	1	m	-	3.46 *	1	ddd	2.2, 5.6, 9.5
**6″**	4.125	1	dd	11.8, 2.2	4.115	1	dd	2.2, 11.9
	3.941	1	dd	11.8, 6.1	3.983	1	dd	5.6, 11.9
**5-OH**	12.595	1	s	-	12.566	1	s	-
**7-OH**	n.a.	-	-	-	n.a.	-	-	-
**1′-OH**	n.a.	-	-	-	n.a.	-	-	-
**2′-OH**	n.a.	-	-	-	n.a.	-	-	-
**2″-OH**	n.a.	-	-	-	5.649	1	d	4.9
**4″-OH**	n.a.	-	-	-	5.400	1	d	6.2
**3″-CO**	-	-	-	-	-	-	-	-
**3″-Ac**	-	-	-	-	2.056	3	s	-
**6″-CO**	-	-	-	-	-	-	-	-
**6″-Ac**	1.730	3	s	-	1.757	3	s	-

n.a.—not assigned due to lack of conclusive correlation from broadened OH signals; *—HSQC readout.

Table S3. ^{1}H NMR data of prepared compounds **4–8** (DMSO-d_6, 30 °C).

Comp.	4				5				6				7				8			
**Atom Number**	**δ H**	**n H**	**m**	**J [Hz]**	**δ H [ppm]**	**n H**	**m**	**J [Hz]**	**δ H**	**n H**	**m**	**J [Hz]**	**δ H**	**n H**	**m**	**J [Hz]**	**δ H**	**n H**	**m**	**J [Hz]**
**2**	-	-	-	-	-	-	-	-	-	-	-	-	-	-	-	-	-	-	-	-
**3**	-	-	-	-	-	-	-	-	-	-	-	-	-	-	-	-	-	-	-	-
**4**	-	-	-	-	-	-	-	-	-	-	-	-	-	-	-	-	-	-	-	-
**4a**	-	-	-	-	-	-	-	-	-	-	-	-	-	-	-	-	-	-	-	-
**5**	-	-	-	-	-	-	-	-	-	-	-	-	-	-	-	-	-	-	-	-
**6**	6.216	1	dd	2.0, 0.2	6.192	1	d	2.0	6.189	1	d	2.0	6.185	1	d	2.0	6.183	1	d	2.1
**7**	-	-	-	-	-	-	-	-	-	-	-	-	-	-	-	-	-	-	-	-
**8**	6.441	1	dd	2.0, 0.9	6.839	1	d	2.0	6.384	1	d	2.0	6.379	1	d	2.0	6.381	1	d	2.1
**8a**	-	-	-	-	-	-	-	-	-	-	-	-	-	-	-	-	-	-	-	-
**1′**	-	-	-	-	-	-	-	-	-	-	-	-	-	-	-	-	-	-	-	-
**2′**	-	-	-	-	-	-	-	-	-	-	-	-	-	-	-	-	-	-	-	-
**3′**	7.542	1	d	2.2	7.516	1	d	2.2	7.515	1	d	2.1	7.516	1	d	2.2	7.520	1	m	-
**4′**	-	-	-	-	-	-	-	-	-	-	-	-	-	-	-	-	-	-	-	-
**5′**	7.521	1	dd	8.3, 2.2	7.521	1	dd	2.2, 8.7	7.518	1	dd	2.1, 9.1	7.514	1	dd	2.2, 9.0	7.512	1	dd	2.2 , 8.2
**6′**	6.845	1	d	8.3	6.825	1	d	8.7	6.824	1	d	9.1	6.821	1	d	9.0	6.822	1	m	-
**1″**	5.436	1	d	7.6	5.436	1	d	7.4	5.434	1	d	7.6	5.429	1	d	7.6	5.428	1	d	7.6
**2″**	3.26 H	1	m	-	3.265	1	m	-	3.27 H	1	m	-	3.26 H	1	m	-	3.256	1	m	-
**3″**	3.26 H	1	m	-	3.256	1	m	-	3.26 H	1	m	-	3.25 H	1	m	-	3.242	1	m	-
**4″**	3.123	1	br.t.	7.5	3.126	1	m	-	3.126	1	m	-	3.12 H	1	m	-	3.125	1	dd	9.7, 8.5
**5″**	3.29 H	1	m	-	3.298	1	m	-	3.30 H	1	m	-	3.300	1	m	-	3.297	1	ddd	9.7, 7.1, 2.2
**6″**	4.157	1	dd	11.8, 2.2	4.155	1	dd	1.8, 11.8	4.155	1	dd	2.2, 11.7	4.152	1	dd	2.4, 11.7	4.149	1	dd	11.7, 2.2
	3.940	1	dd	11.8, 7.0	3.949	1	dd	7.0 , 11.8	3.950	1	dd	7.1, 11.7	3.947	1	dd	7.1, 11.7	3.948	1	dd	11.7, 7.1
**5-OH**	12.612	1	s	-	12.612	1	s	-	12.610	-	-	-	12.608	1	s	-	12.607	1	s	-
**7-OH/ 1′-OH/ 2′-OH**	10.962 *	1	br.s.	-	n.a.	-	-	-	n.a.	-	-	-	n.a	-	-	-	10.819	1	s	-
	9.725 *	1	br.s.	-	n.a.	-	-	-	n.a.	-	-	-	n.a.	-	-	-	9.655	1	s	-
	9.249 *	1	br.s.	-	n.a.	-	-	-	n.a.	-	-	-	n.a.	-	-	-	9.183	1	s	-
**2″-OH**	5.173	1	br.s.	-	n.a.	-	-	-	n.a.	-	-	-	n.a.	-	-	-	5.341	1	s	-
**3″-OH**	5.377	1	br.d	2.6	n.a.	-	-	-	n.a.	-	-	-	n.a.	-	-	-	5.147	1	s	-
**4″-OH**	5.173	1	br.s.	-	n.a.	-	-	-	n.a.	-	-	-	n.a.	-	-	-	5.147	1	s	-

Table S3. *Cont.*

Comp.	4				5				6				7				8			
**Atom Number**	**δ H**	**n H**	**m**	**J [Hz]**	**δ H [ppm]**	**n H**	**m**	**J [Hz]**	**δ H**	**n H**	**m**	**J [Hz]**	**δ H**	**n H**	**m**	**J [Hz]**	**δ H**	**n H**	**m**	**J [Hz]**
**1‴**	-	-	-	-	-	-	-	-	-	-	-	-	-	-	-	-	-	-	-	-
**2‴**	1.961	2	m	-	1.968	2	m	-	1.971	2	m	-	1.970	2	m	-	1.969	2	m	-
**3‴**	1.246	2	m	-	1.238 H	2	m	-	1.24 H	2	m	-	1.23 H	2	m	-	1.233	2	m	-
**4‴**	0.649	3	t	7.4	1.030	2	m	-	1.05 H	2	m	-	1.04 H	2	m	-	1.035	2	m	-
**5‴**	-	-	-	-	1.118	2	m	-	1.09 H	2	m	-					1.078	2	m	-
**6‴**	-	-	-	-	0.778	3	t	7.0	1.12 H	2	m	-					1.236	2	m	-
**7‴**	-	-	-	-	-	-	-	-	1.21 H	2	m	-	1.05–1.22 H	10	m	-	1.142	2	m	-
**8‴**	-	-	-	-	-	-	-	-	0.837	3	t	7.2					1.191	2	m	-
**9‴**	-	-	-	-	-	-	-	-	-	-	-	-					1.210	2	m	-
**10‴**	-	-	-	-	-	-	-	-	-	-	-	-	1.23 H	2	m	-	1.233	2	m	-
**11‴**	-	-	-	-	-	-	-	-	-	-	-	-	1.25 H	2	m	-				
**12‴**	-	-	-	-	-	-	-	-	-	-	-	-	0.857	3	t	7.1	1.233	6	m	-
**13‴**	-	-	-	-	-	-	-	-	-	-	-	-	-	-	-	-				
**14‴**	-	-	-	-	-	-	-	-	-	-	-	-	-	-	-	-	1.233	2	m	-
**15‴**	-	-	-	-	-	-	-	-	-	-	-	-	-	-	-	-	1.250	2	m	-
**16‴**	-	-	-	-	-	-	-	-	-	-	-	-	-	-	-	-	0.849	3	t	7.1

n.a.—not assigned due to lack of conclusive correlation from broadened OH signals; [3C] … three overlapped carbon signals; *—HSQC readout.

Table S4. ^{13}C NMR data of prepared compounds **9–11**.

**Comp.**	**9** [a]		**10** [b]		**11** [c]	
**Atom Number**	**δ C**	**m C**	**δ C**	**m C**	**δ C**	**m C**
**2**	157.33	s	158.47	s	158.1 [M]	s
**3**	133.35	s	135.65	s	135.4 [M]	s
**4**	177.08	s	179.18	s	179.0 [M]	s
**4a**	103.53	s	105.63	s	105.2 [M]	s
**5**	159.53	s	163.23	S	163.1 [M]	s
**6**	99.22	d	100.33	d	100.6 [H]	d
**7**	164.68	s	166.51	s	167.6 [M]	s
**8**	94.62	d	95.09	d	95.2 [H]	d
**8a**	156.11	s	158.12	s	158.2 [M]	s
**9**	147.40	s	151.27	s	151.4 [M]	s
**10**	143.22	s	147.38	s	147.4 [M]	s
**11**	116.42	d	118.13	d	118.0 [H]	d
**12**	121.38	s	122.83	s	122.6 [M]	s
**13**	122.26	d	123.28	d	123.1 [H]	d
**14**	114.99	d	116.62	d	116.6 [H]	d
**1′**	102.07	d	104.77	d	104.5 [H]	d
**2′**	73.56	d	76.46	d	76.3 [H]	d
**3′**	75.34	d	78.91	d	78.9 [H]	d
**4′**	69.24	d	71.65	d	71.8 [H]	d
**5′**	73.18	d	76.29	d	76.2 [H]	d
**6′**	62.66	t	64.69	t	64.7 [H]	t
**5-OH**	-	-	-	-	-	-
**7-OH**	-	-	-	-	-	-
**9-OH**	-	-	-	-	-	-
**10-OH**	-	-	-	-	-	-
**2′-OH**	-	-	-	-	-	-
**3′-OH**	-	-	-	-	-	-
**4′-OH**	-	-	-	-	-	-
**1″**	175.30	s	173.77	s	174.0 [M]	s
**2″**	32.96	t	34.42	t	34.7 [H]	t
**3″**	20.85	t	25.30	t	25.5 [H]	t
**4″**	36.37	t	25.53	t	29.6 [H]	t
**5″**	182.31	s	34.95	t	29.7 [H]	t
**6″**			176.29	s	29.8 [H]	t
**7″**	-	-	-	-	30.0 [H]	t
**8″**	-	-	-	-	29.9 [H]	t
**9″**	-	-	-	-	30.0 [H]	t
**10″**	-	-	-	-	26.7 [H]	t
**11″**	-	-	-	-	37.1 [H]	t
**12″**	-	-	-	-	179.3 [H]	s
**5″-OH**	-	-	-	-	-	-
**6″-OH**	-	-	n.d.	-	-	-
**12″-OH**	-	-	-	-	-	-

[a] D_2O, 293.2 K, 150.94 MHz for ^{13}C; [b] pyridin, 293.2 K, 150.94 MHz for ^{13}C; [c] pyridin, 293.2 K, 150.94 MHz for ^{13}C; H—HSQC readout; M—HMBC readout; n.d.—not detected.

Table S5. ^{1}H NMR data of prepared compounds **9**–**11**.

**Comp.**	**9** [a]				**10** [b]				**11** [c]			
**Atom Number**	**δ H**	**n H**	**m**	**J [Hz]**	**δ H [ppm]**	**n H**	**m**	**J [Hz]**	**δ H**	**n H**	**m**	**J [Hz]**
**2**	-	-	-	-	-	-	-	-	-	-	-	-
**3**	-	-	-	-	-	-	-	-	-	-	-	-
**4**	-	-	-	-	-	-	-	-	-	-	-	-
**4a**	-	-	-	-	-	-	-	-	-	-	-	-
**5**	-	-	-	-	-	-	-	-	-	-	-	-
**6**	5.874	1	br.s.	-	6.731	1	d	2.0	6.738	1	br.s.	-
**7**	-	-	-	-	-	-	-	-	-	-	-	-
**8**	5.852	1	br.s.	-	6.694	1	d	2.0	6.787	1	br.s.	-
**8a**	-	-	-	-	-	-	-	-	-	-	-	-
**9**	-	-	-	-	-	-	-	-	-	-	-	-
**10**	-	-	-	-	-	-	-	-	-	-	-	-
**11**	7.251	1	br.s.	-	8.361	1	d	2.1	8.361	1	br.s.	-
**12**	-	-	-	-	-	-	-	-	-	-	-	-
**13**	7.032	1	br.d	8.4	8.113	1	dd	8.4, 2.1	8.144	1	br.d.	8.1
**14**	6.546	1	d	8.4	7.313	1	d	8.4	7.328	1	br.d.	8.1
**1′**	4.726	1	d	7.8	6.157	1	m	-	6.206	1	br.d.	4.8
**2′**	3.420	1	dd	7.8, 9.2	4.346	1	m	-	4.355	1	m	-
**3′**	3.344	1	dd	9.2, 9.0	4.346	1	m	-	4.355	1	m	-
**4′**	3.283	1	m	-	4.059	1	ddd	9.8, 6.0, 2.8	4.042	1	m	-
**5′**	3.283	1	m	-	4.128	1	ddd	9.8, 6.1, 1.9	4.169	1	m	-
**6′**	3.952	2	m	-	4.841	1	dd	11.6, 1.9	4.886	1	br.d.	11.6
					4.685	1	dd	11.6, 6.1	4.720	1	dd	11.6, 5.8
**5-OH**	n.d.	-	-	-	13.205	1	br.s.	-	n.d.	-	-	-
**7-OH/**	n.d.	-	-	-	-	-	-	-	n.d.	-	-	-
**9-OH/**	n.d.	-	-	-	-	-	-	-	n.d.	-	-	-
**10-OH**	n.d.	-	-	-	-	-	-	-	n.d.	-	-	-
**2′-OH**	n.d.	-	-	-	-	-	-	-	n.d.	-	-	-
**3′-OH**	n.d.	-	-	-	-	-	-	-	n.d.	-	-	-
**4′-OH**	n.d.	-	-	-	-	-	-	-	n.d.	-	-	-
**1″**	-	-	-	-	-	-	-	-	n.d.	-	-	-
**2″**	1.911	2	m	-	2.332	1	m	-	-	-	-	-
					2.269	1	m	-				
**3″**	1.441	2	m	-	1.672	2	m	-	2.269	2	m	-
**4″**	1.898	2	m	-	1.733	2	m	-	1.534	2	m	-
**5″**	-	-	-	-	2.424	2	t	7.5	1.171	2	m	-
**6″**	-	-	-	-	-	-	-	-	1.124	2	m	-
**7″**	-	-	-	-	-	-	-	-	1.138	2	m	-
**8″**	-	-	-	-	-	-	-	-	1.211	2	m	-
**9″**	-	-	-	-	-	-	-	-	1.291	2	m	-
**10″**	-	-	-	-	-	-	-	-	1.473	2	m	-
**11″**	-	-	-	-	-	-	-	-	1.901	2	m	-
**12″**	-	-	-	-	-	-	-	-	2.634	2	m	-
**5″-OH**	n.d.	-	-	-	n.d.	-	-	-	-	-	-	-
**6″-OH**	-	-	-	-	-	-	-	-	-	-	-	-
**12″-OH**	-	-	-	-	-	-	-	-	n.d.	-	-	-

[a] D_2O, 293.2 K, 600.23 MHz for ^{1}H; [b] pyridin, 293.2 K, 600.23 MHz for ^{1}H; [c] pyridin, 293.2 K, 600.23 MHz for ^{1}H; H—HSQC readout; M—HMBC readout; n.d.—not detected.

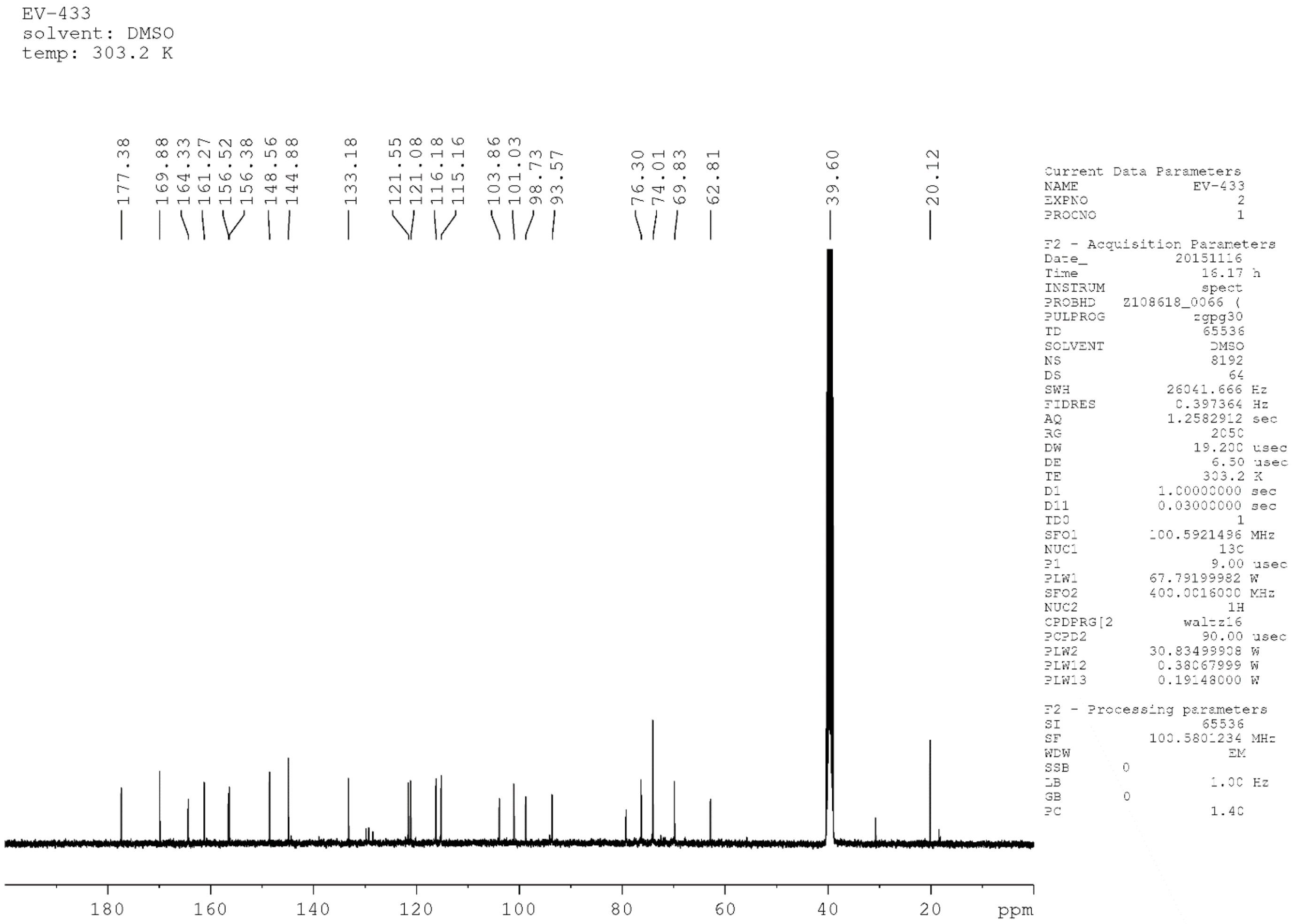

Figure S2. ^{13}C NMR spectrum of compound **2**.

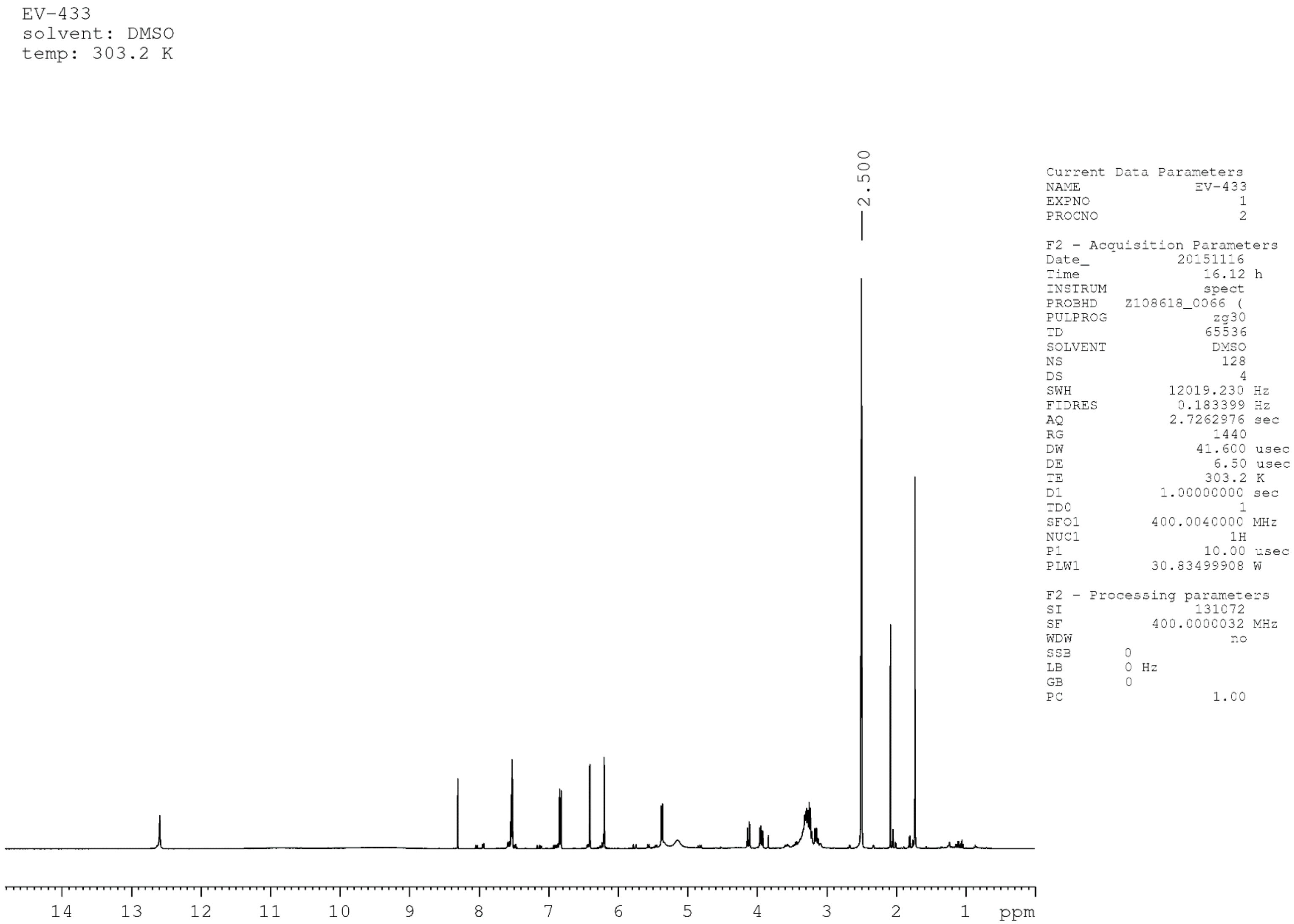

Figure S3. ^{1}H NMR spectrum of compound **2**.

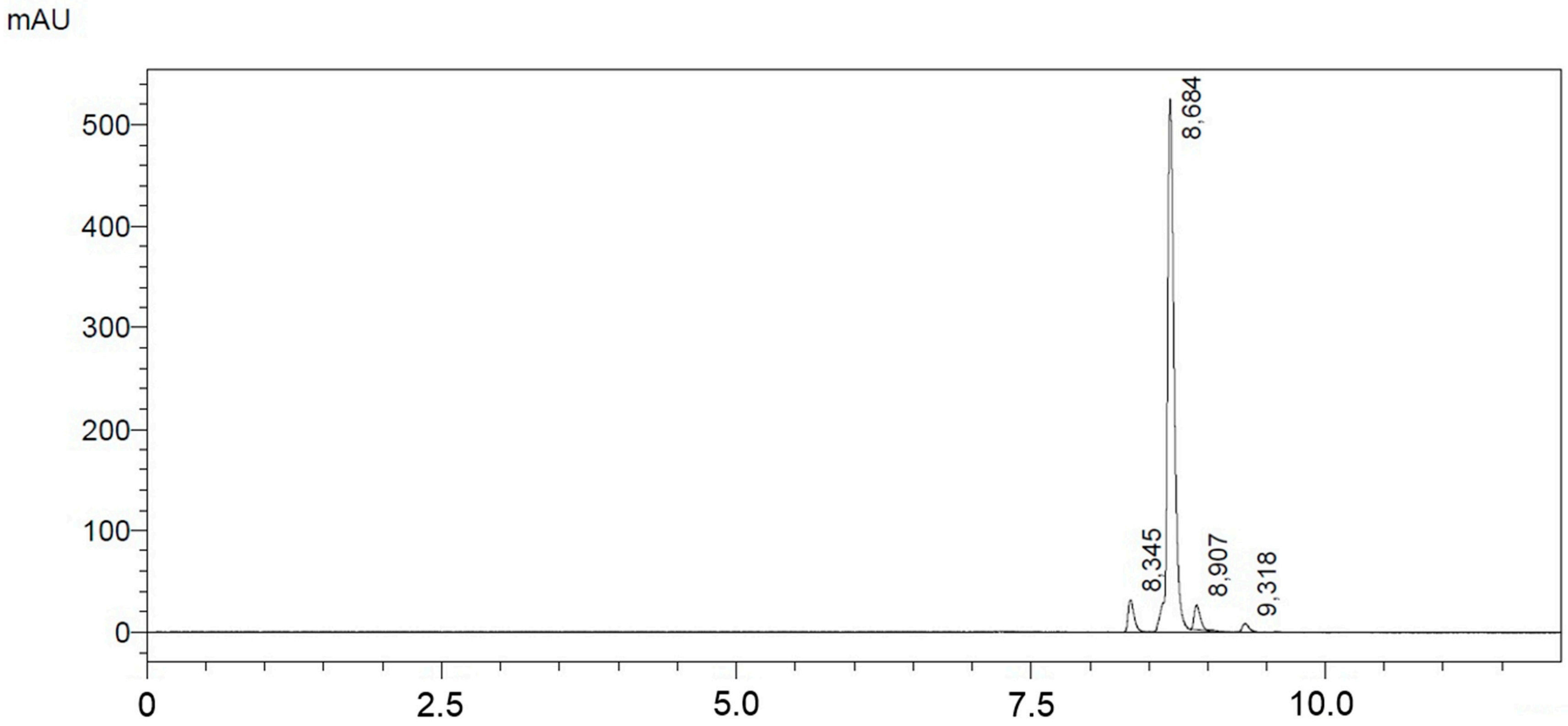

Figure S4. HPLC chromatogram of compound **2**.

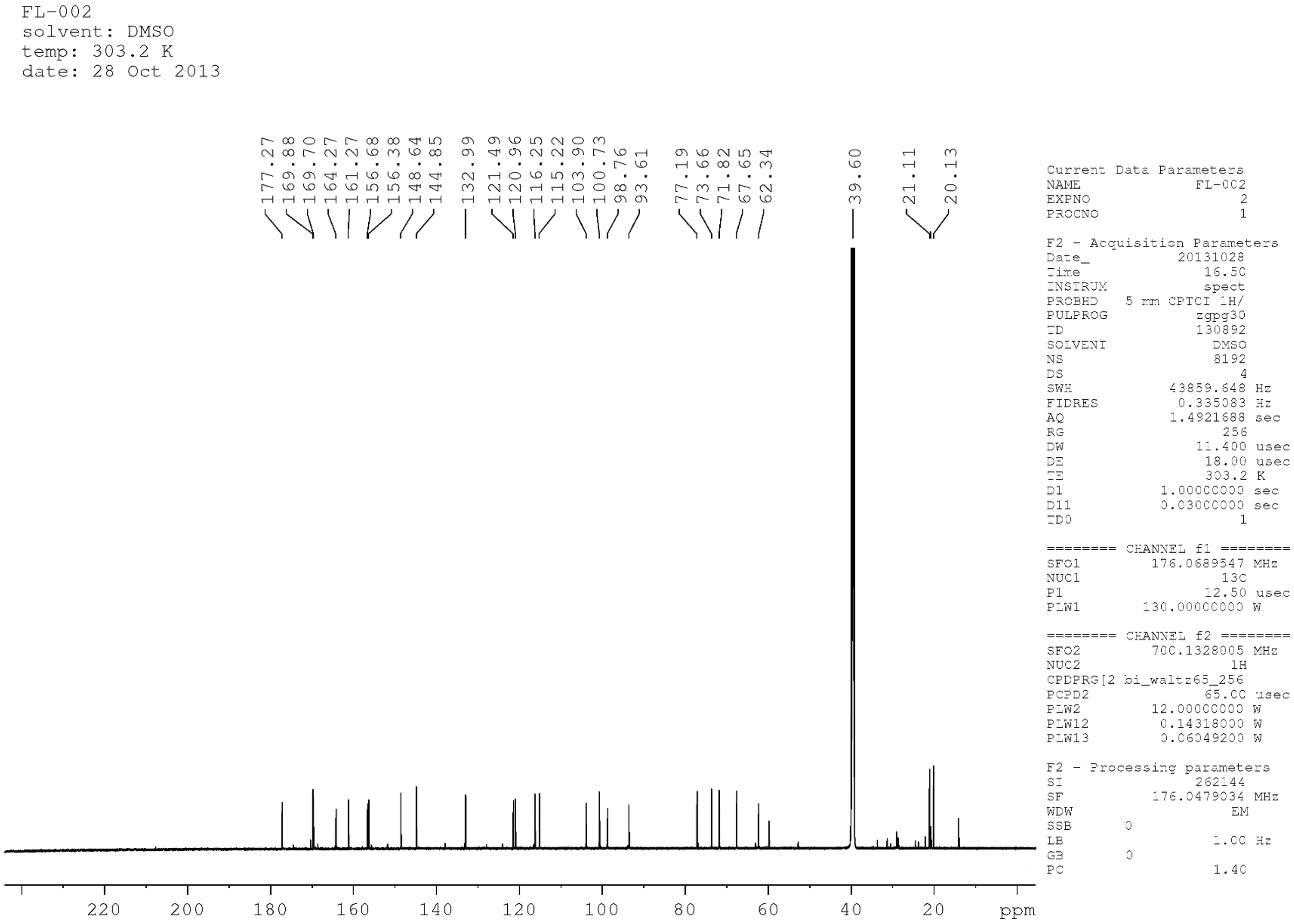

Figure S5. ^{13}C NMR spectrum of compound **3**.

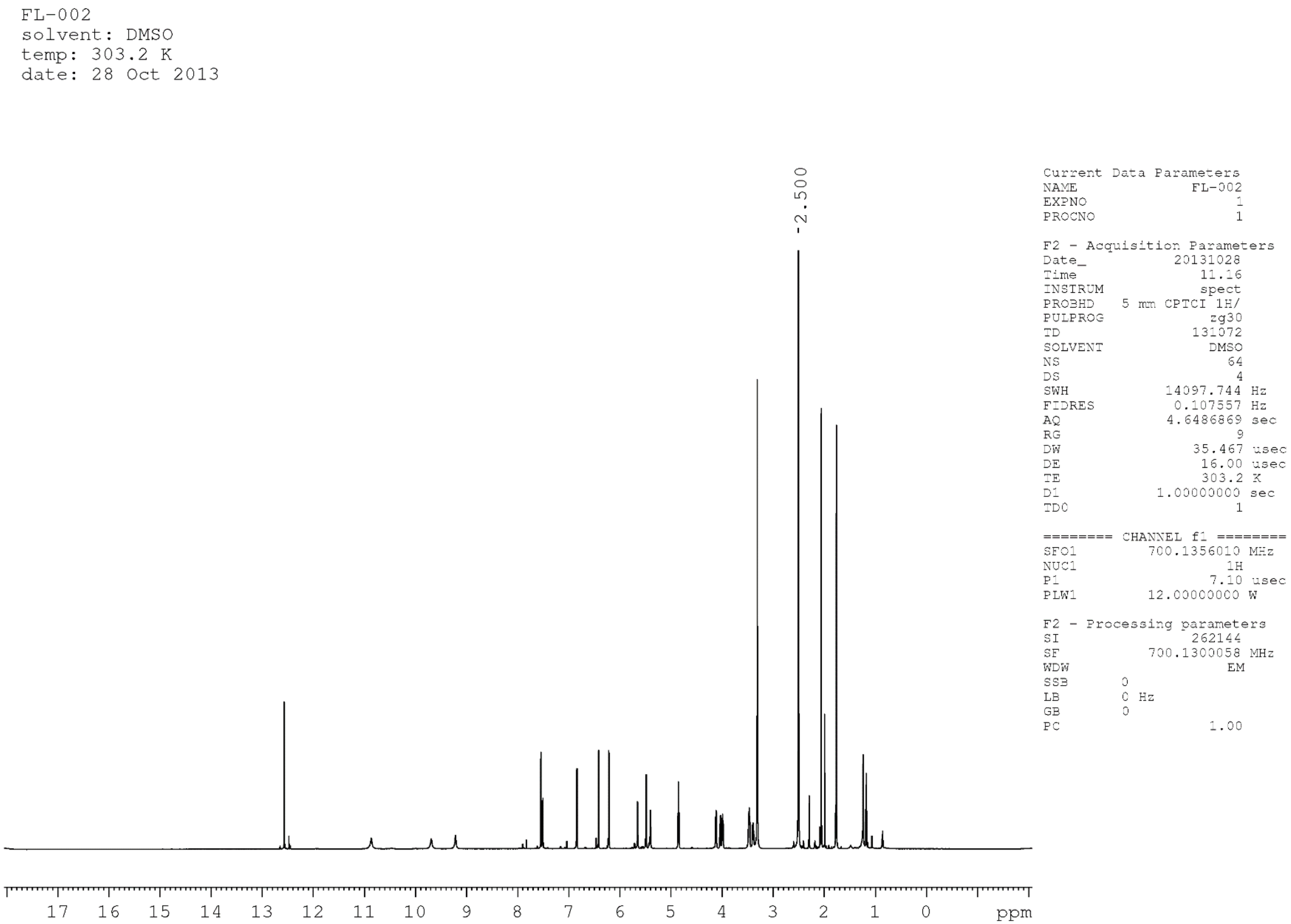

Figure S6. ^{1}H NMR spectrum of compound **3**.

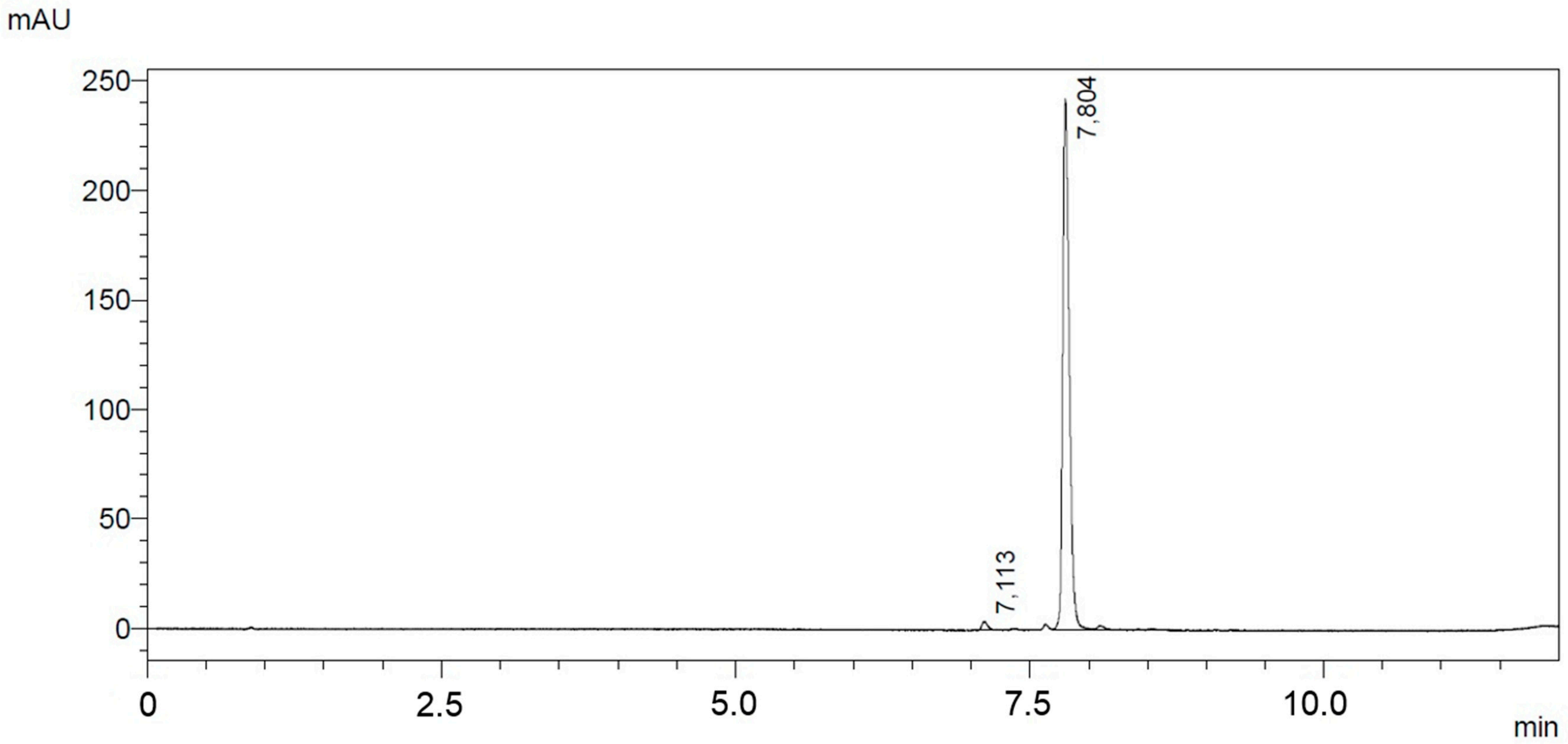

Figure S7. HPLC chromatogram of compound **3**.

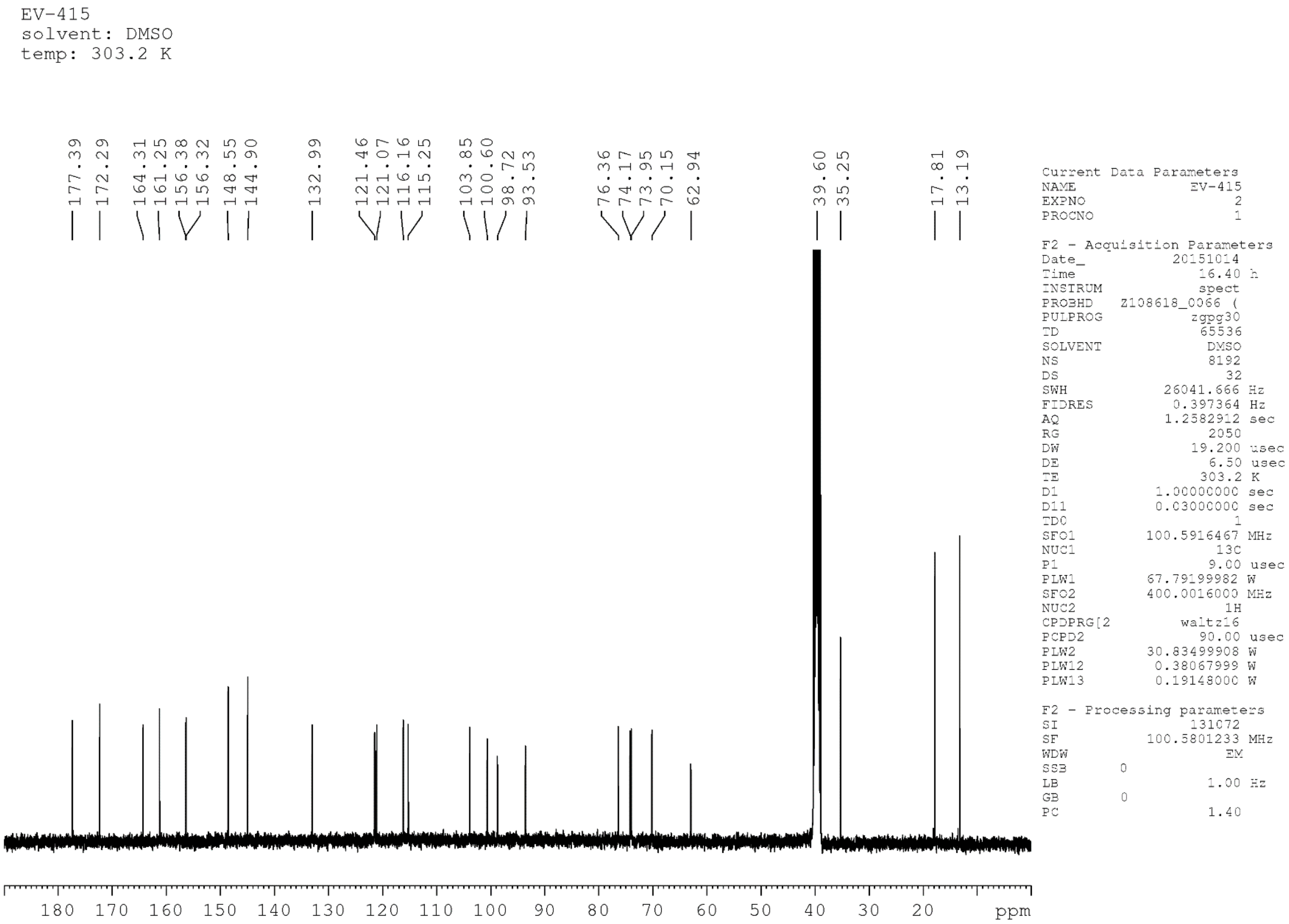

Figure S8. ^{13}C NMR spectrum of compound **4**.

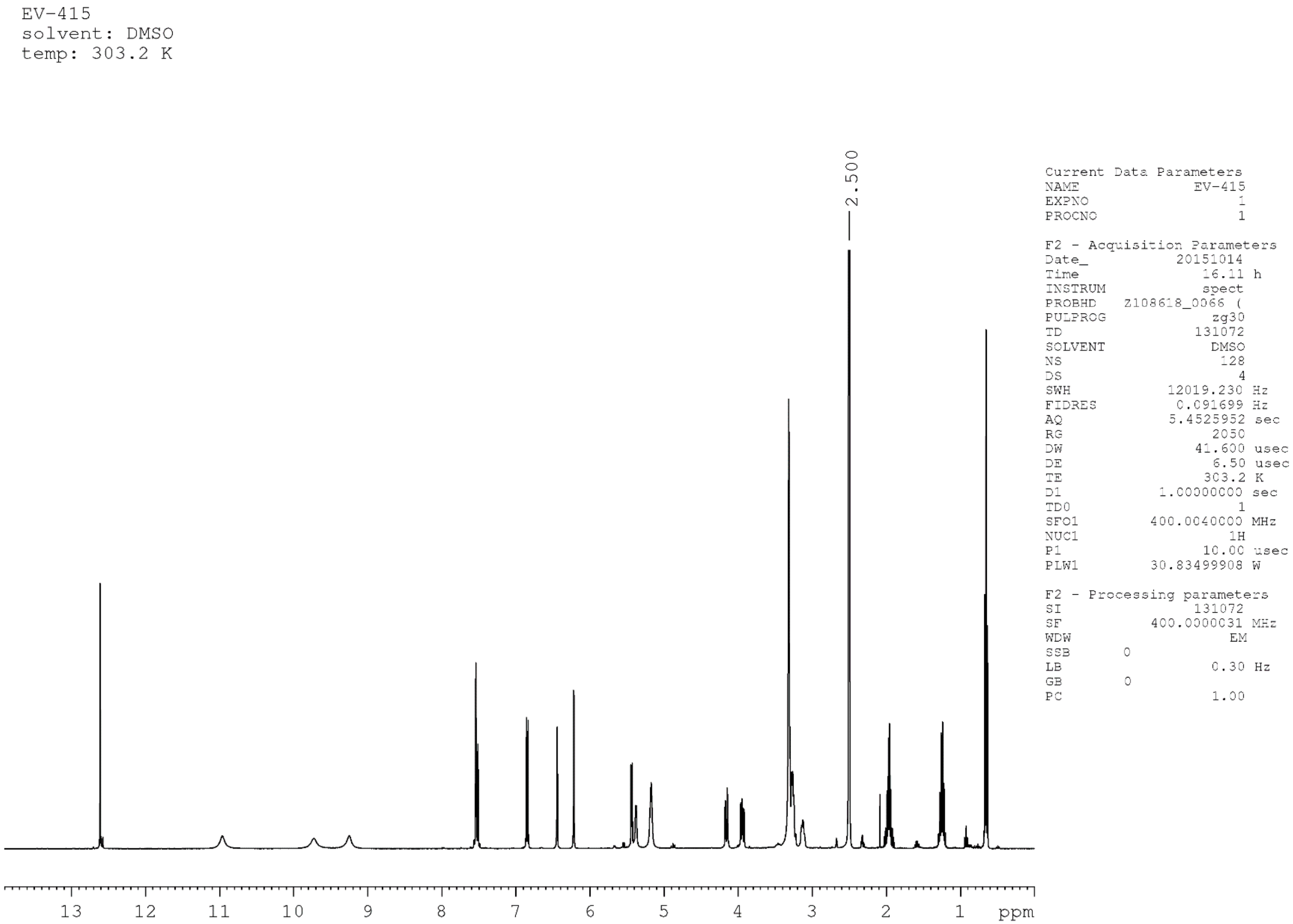

Figure S9. ^{1}H NMR spectrum of compound **4**.

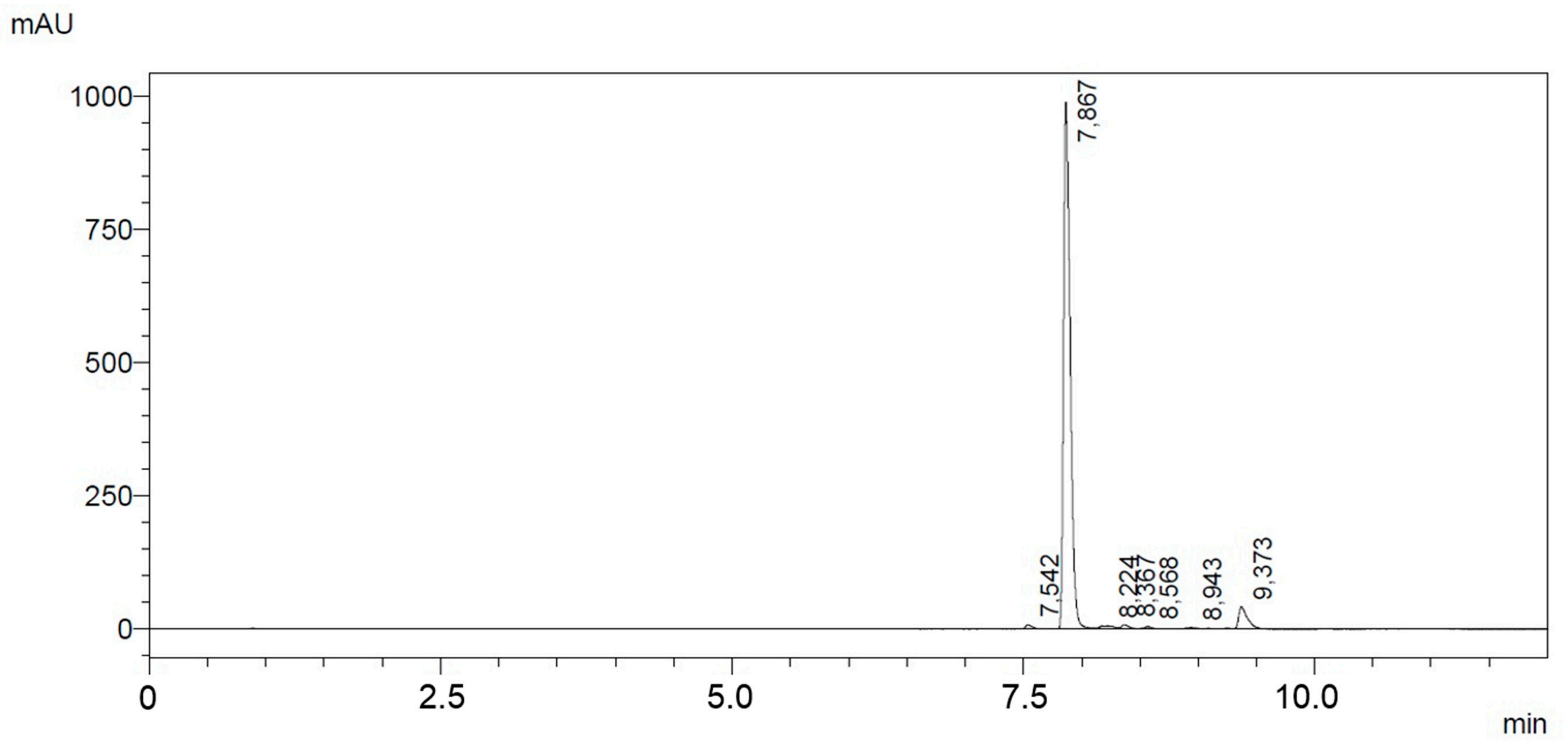

Figure S10. HPLC chromatogram of compound **4**.

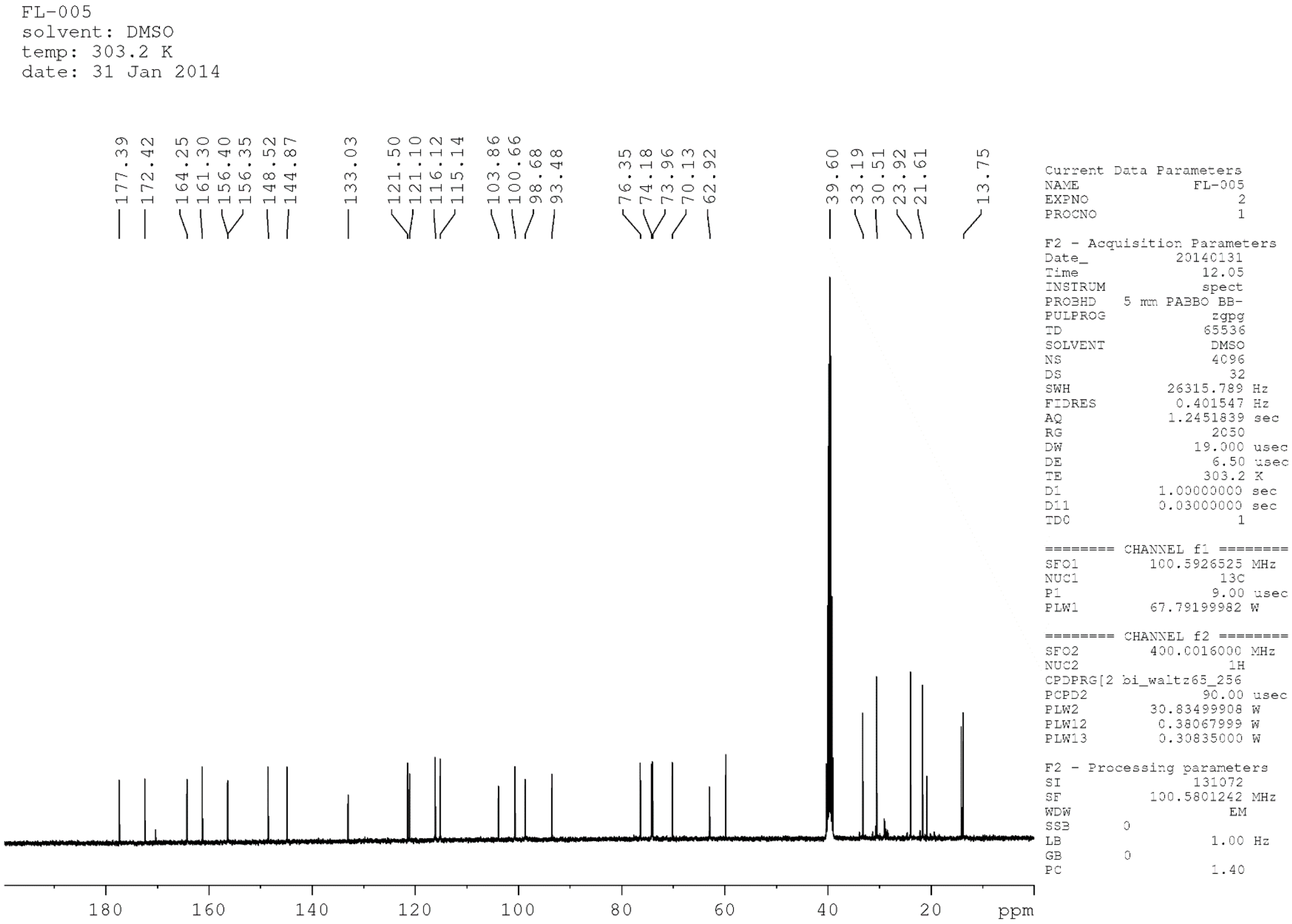

Figure S11. ^{13}C NMR spectrum of compound **5**.

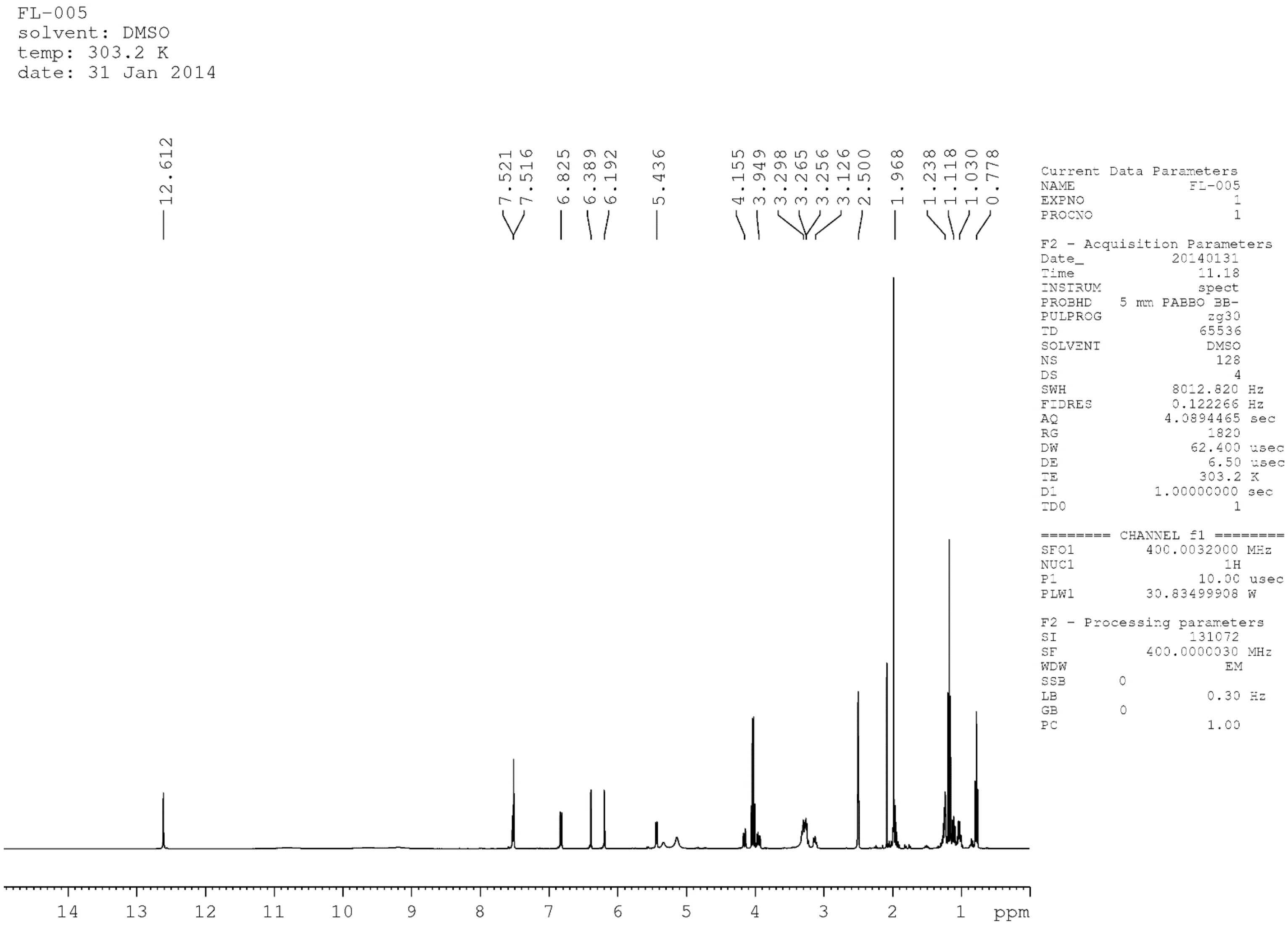

Figure S12. ^{1}H NMR spectrum of compound **5**.

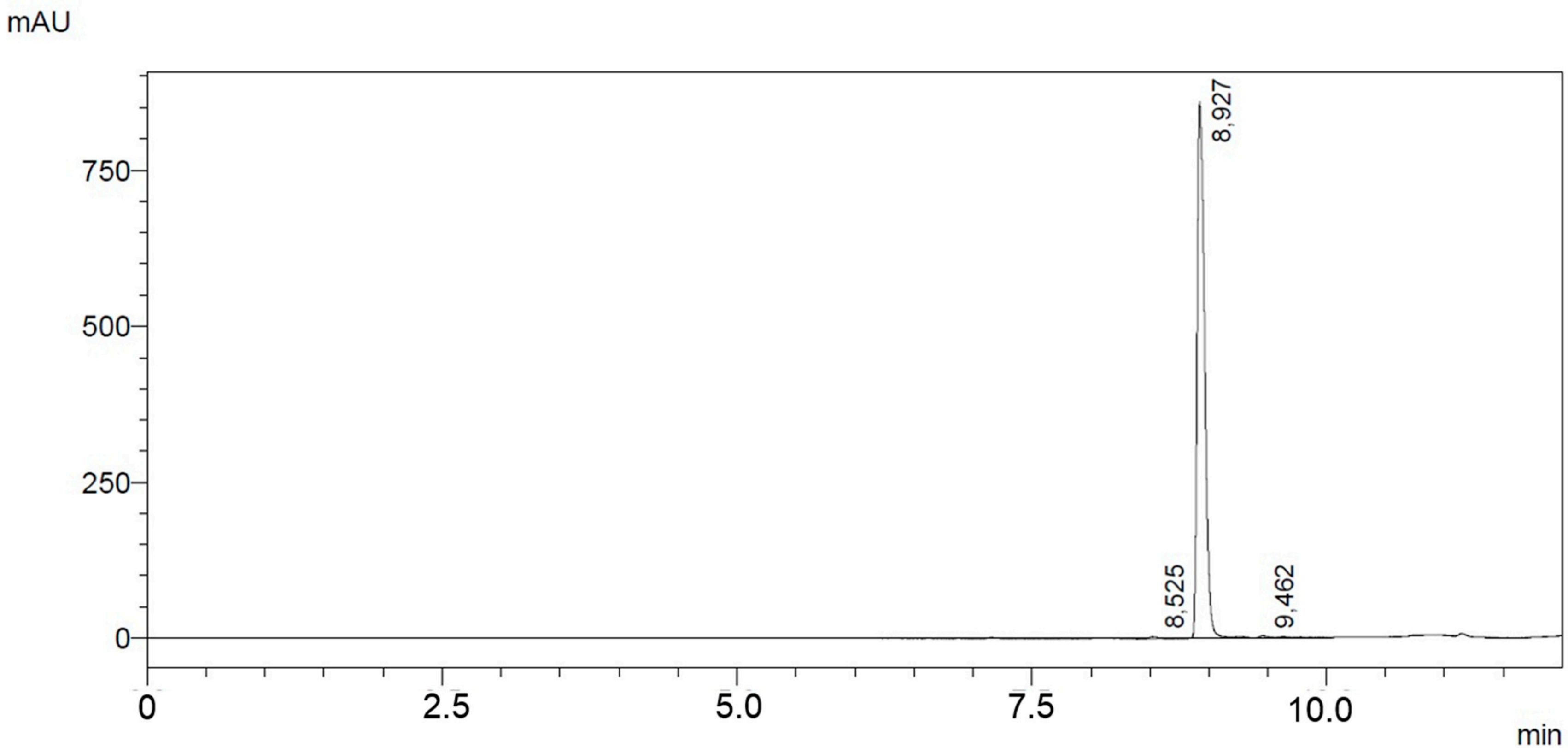

Figure S13. HPLC chromatogram of compound **5**.

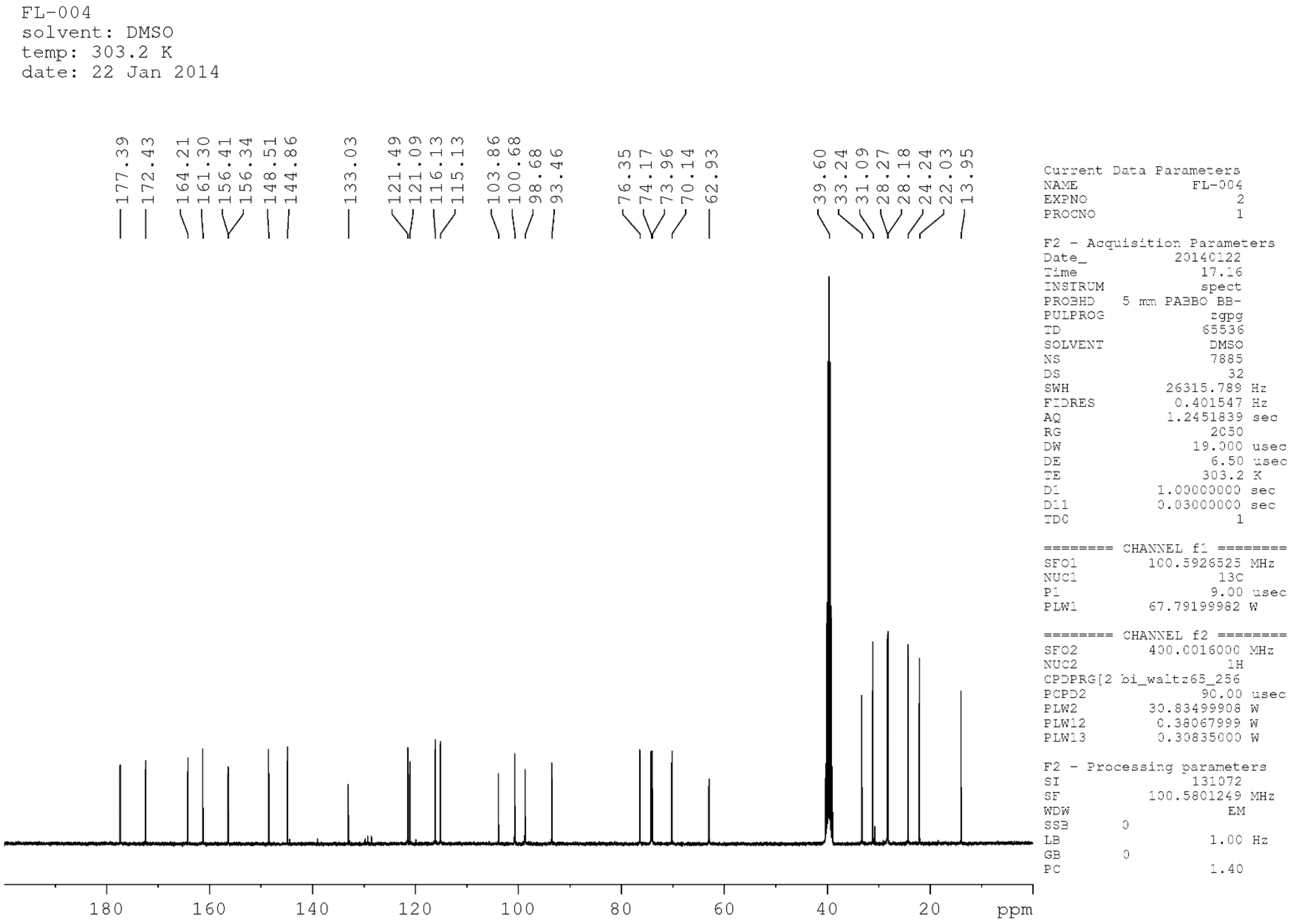

Figure S14. ^{13}C NMR spectrum of compound **6**.

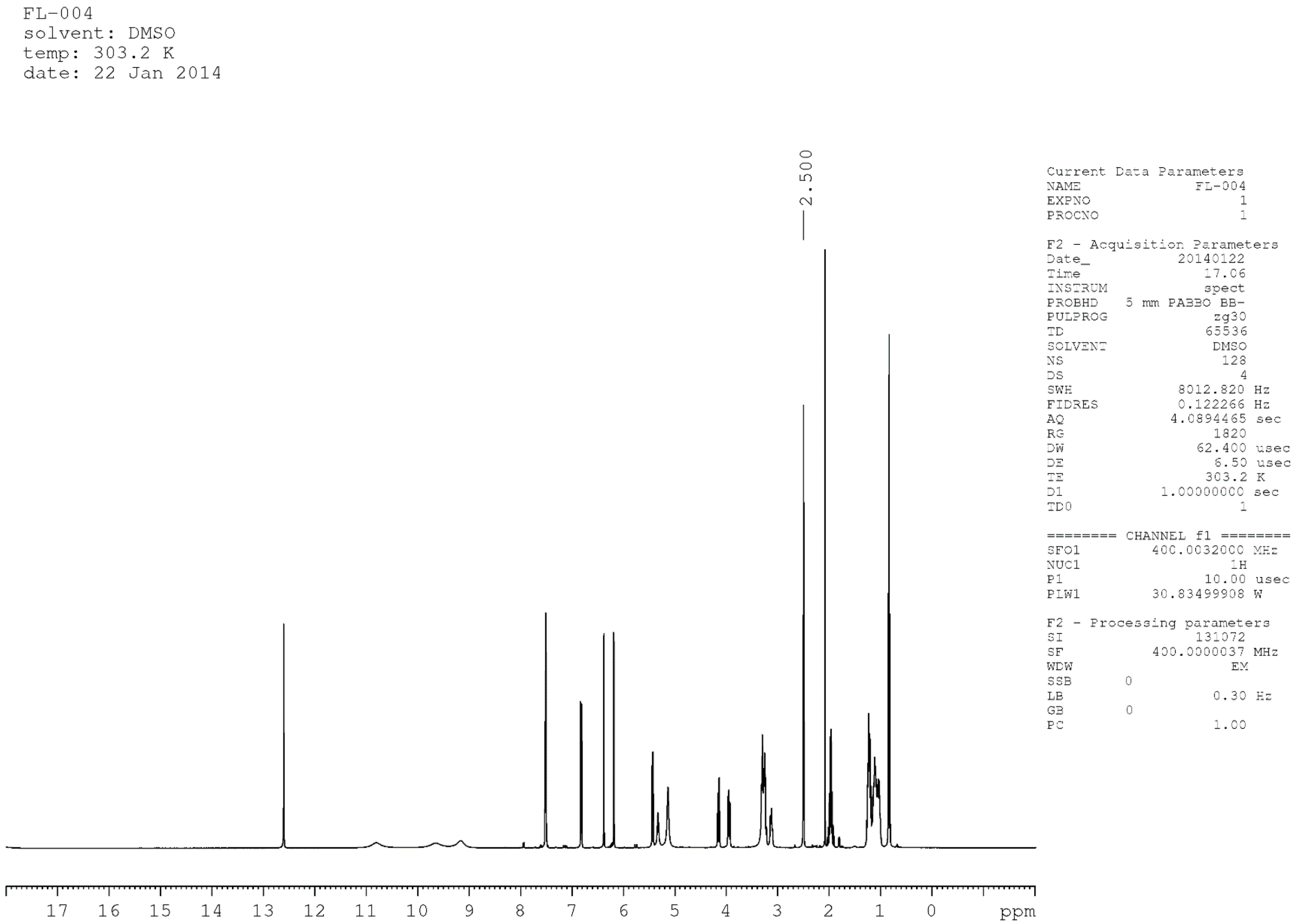

Figure S15. ^{1}H NMR spectrum of compound **6**.

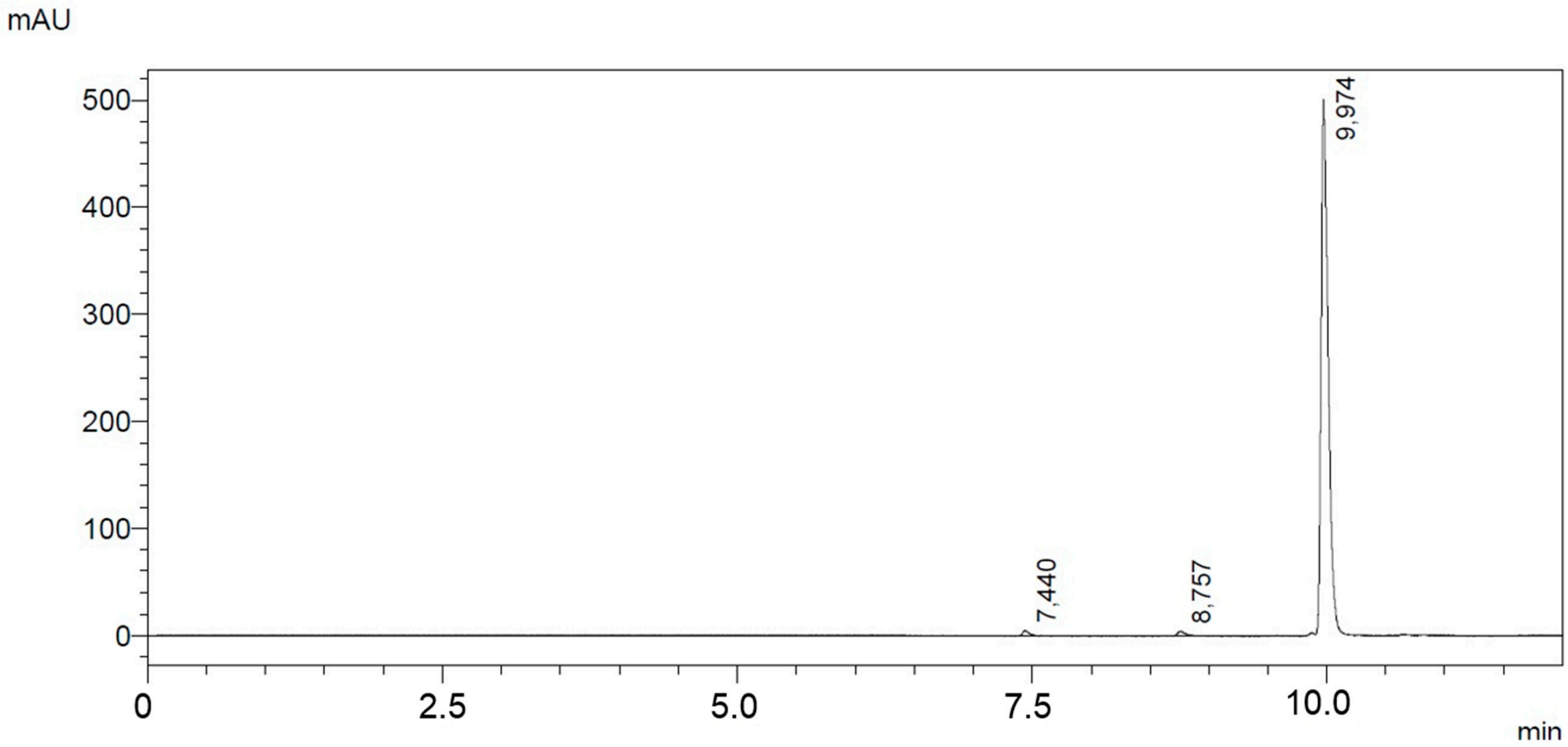

Figure S16. HPLC chromatogram of compound **6**.

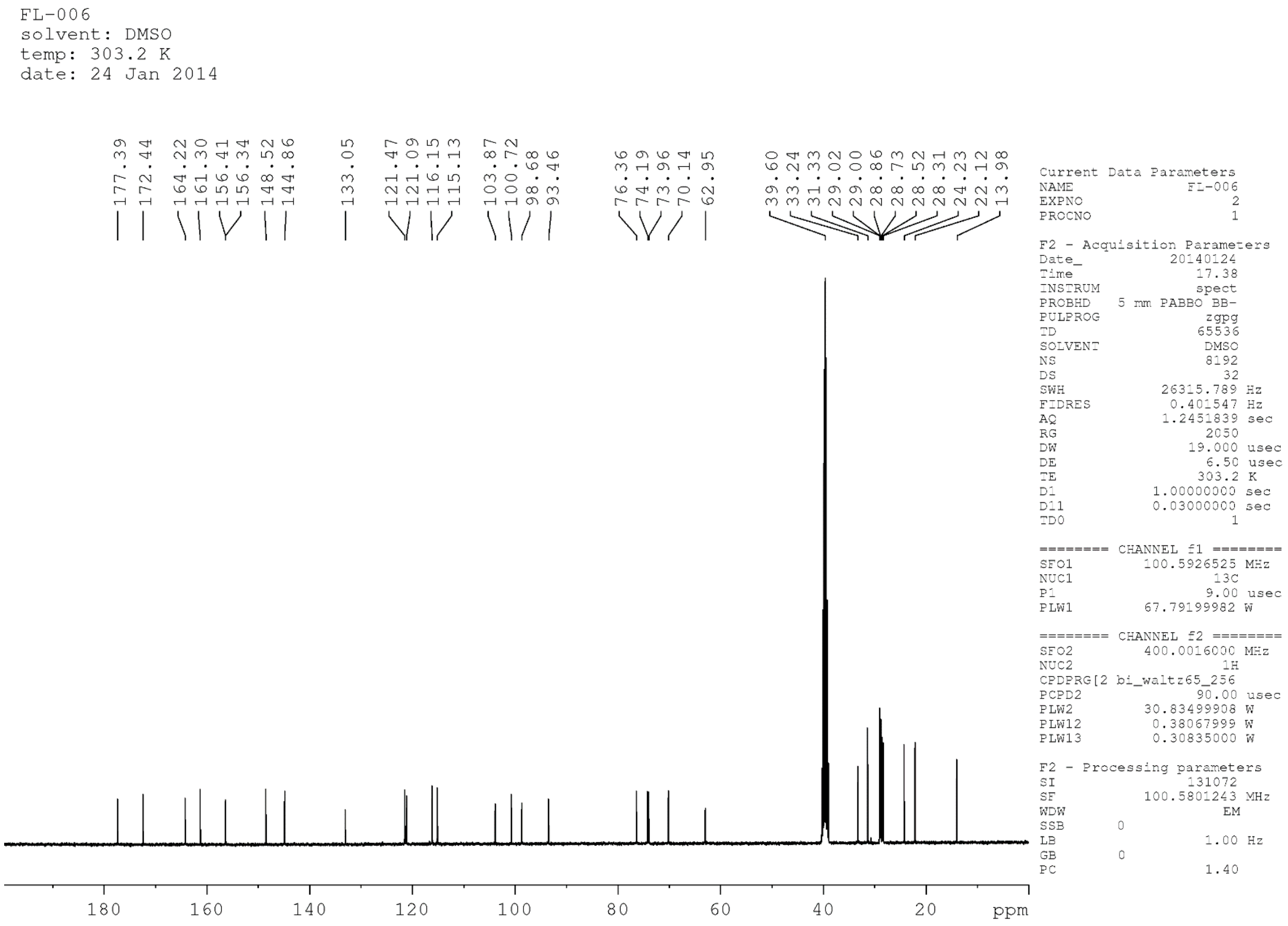

Figure S17. ^{13}C NMR spectrum of compound **7**.

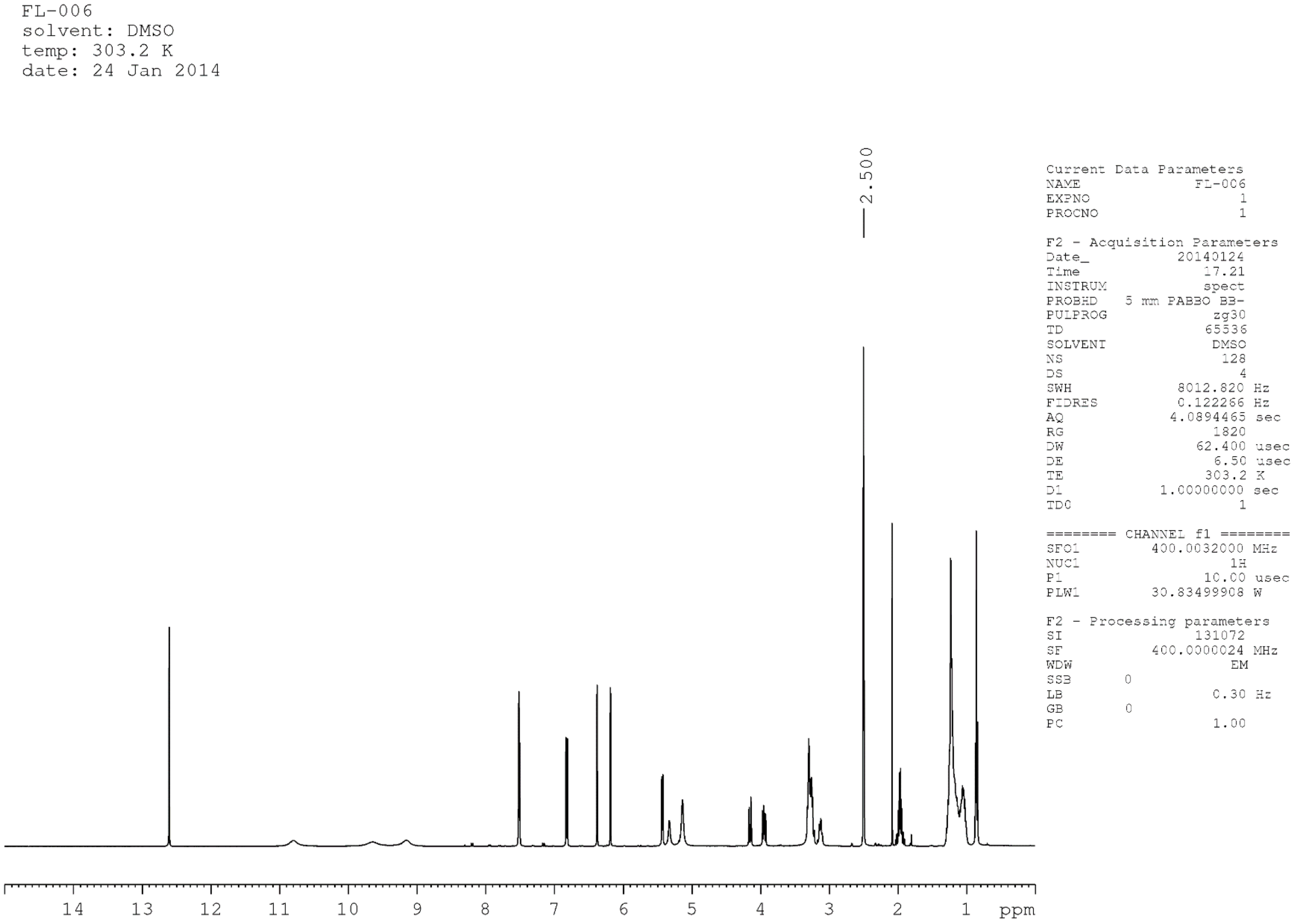

Figure S18. ^{1}H NMR spectrum of compound **7**.

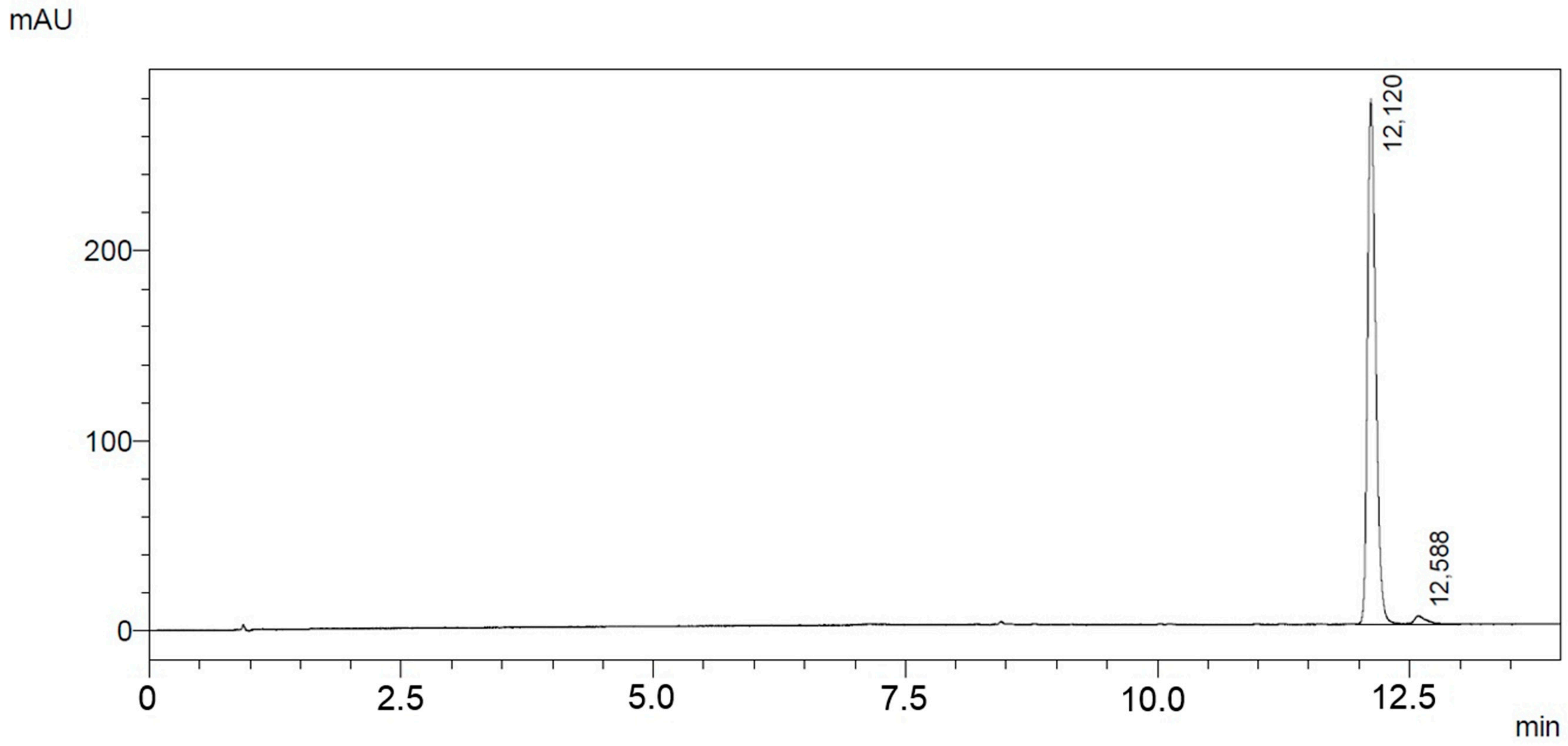

Figure S19. HPLC chromatogram of compound **7**.

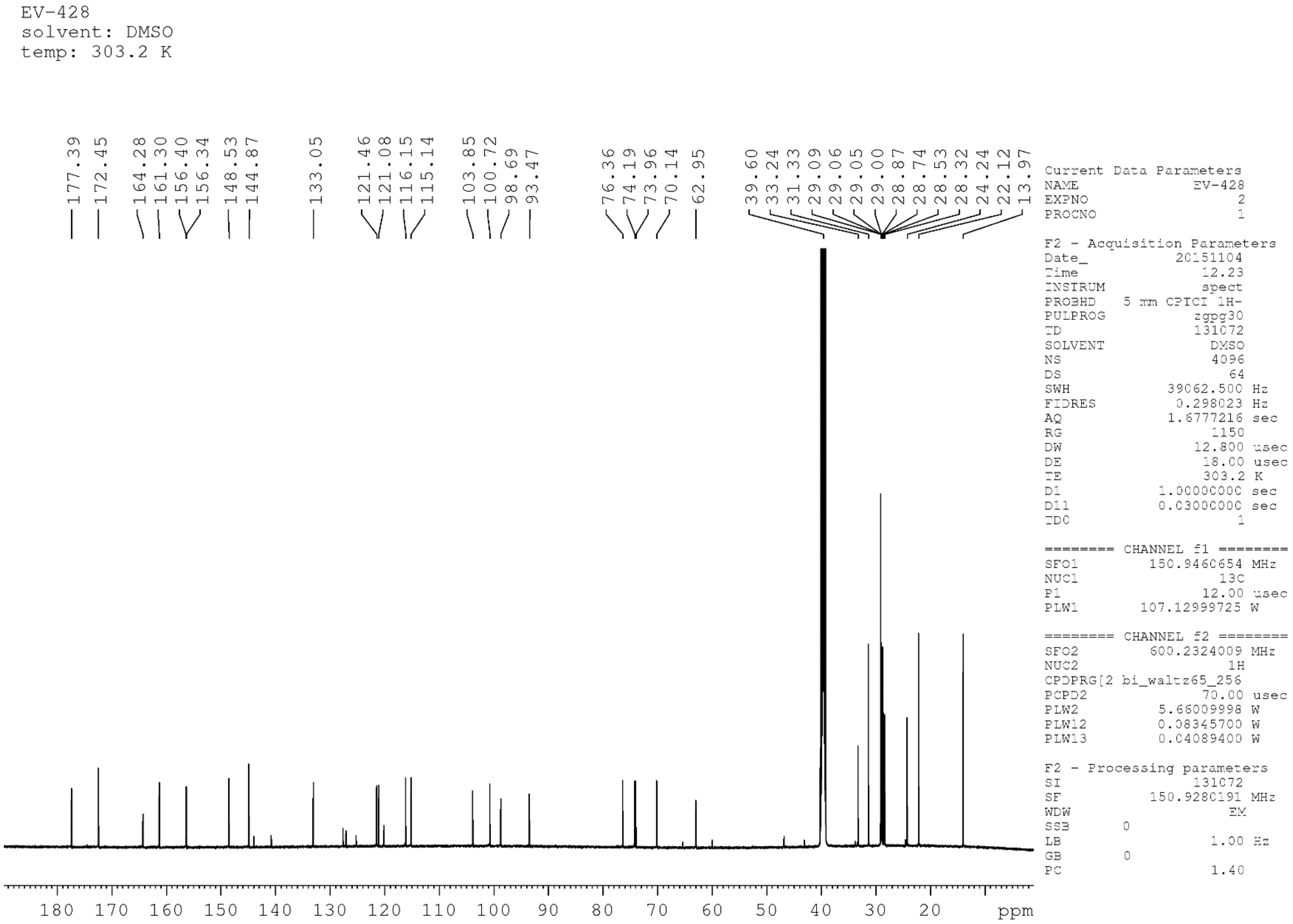

Figure S20. ^{13}C NMR spectrum of compound **8**.

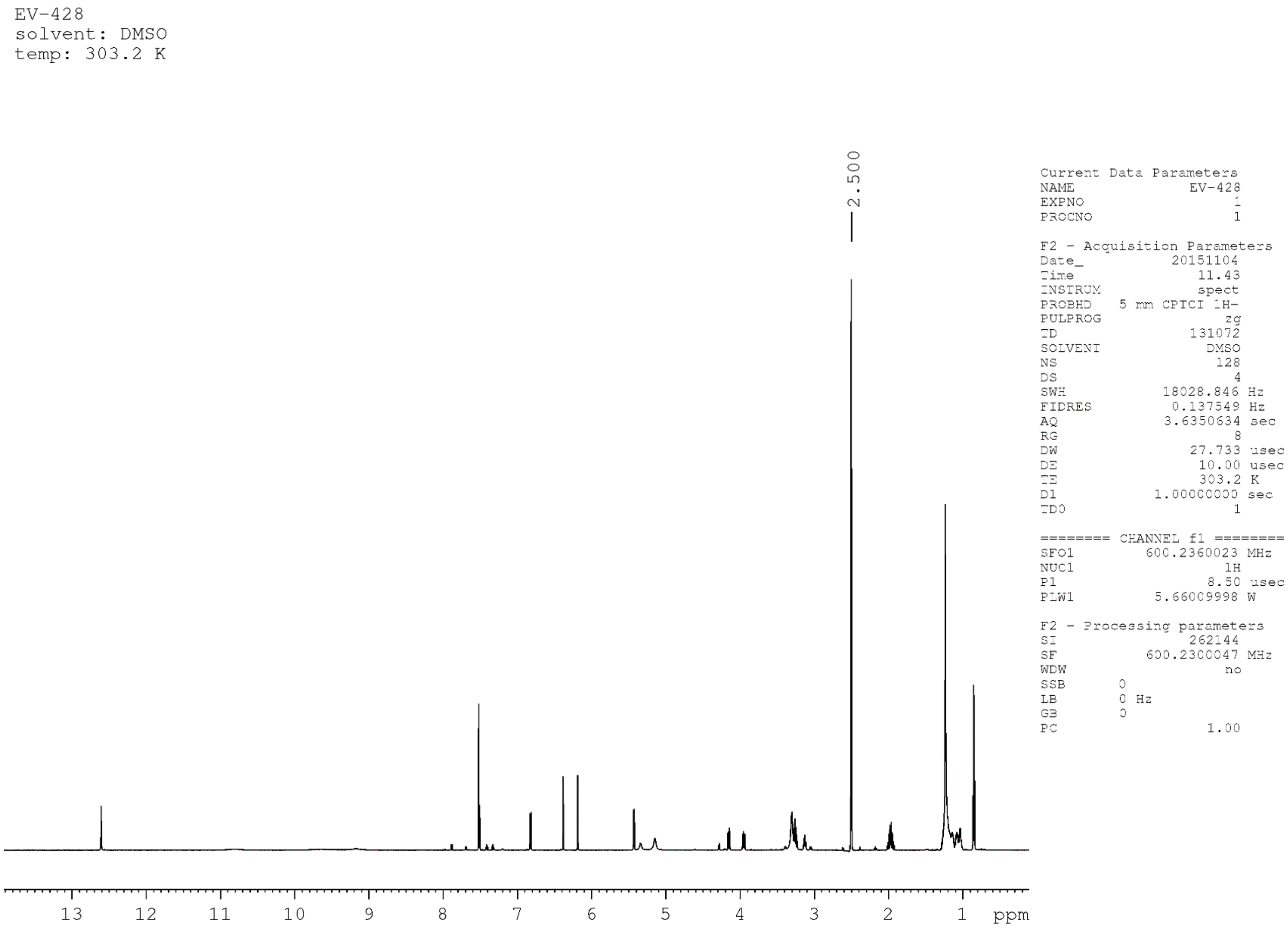

Figure S21. ^{1}H NMR spectrum of compound **8**.

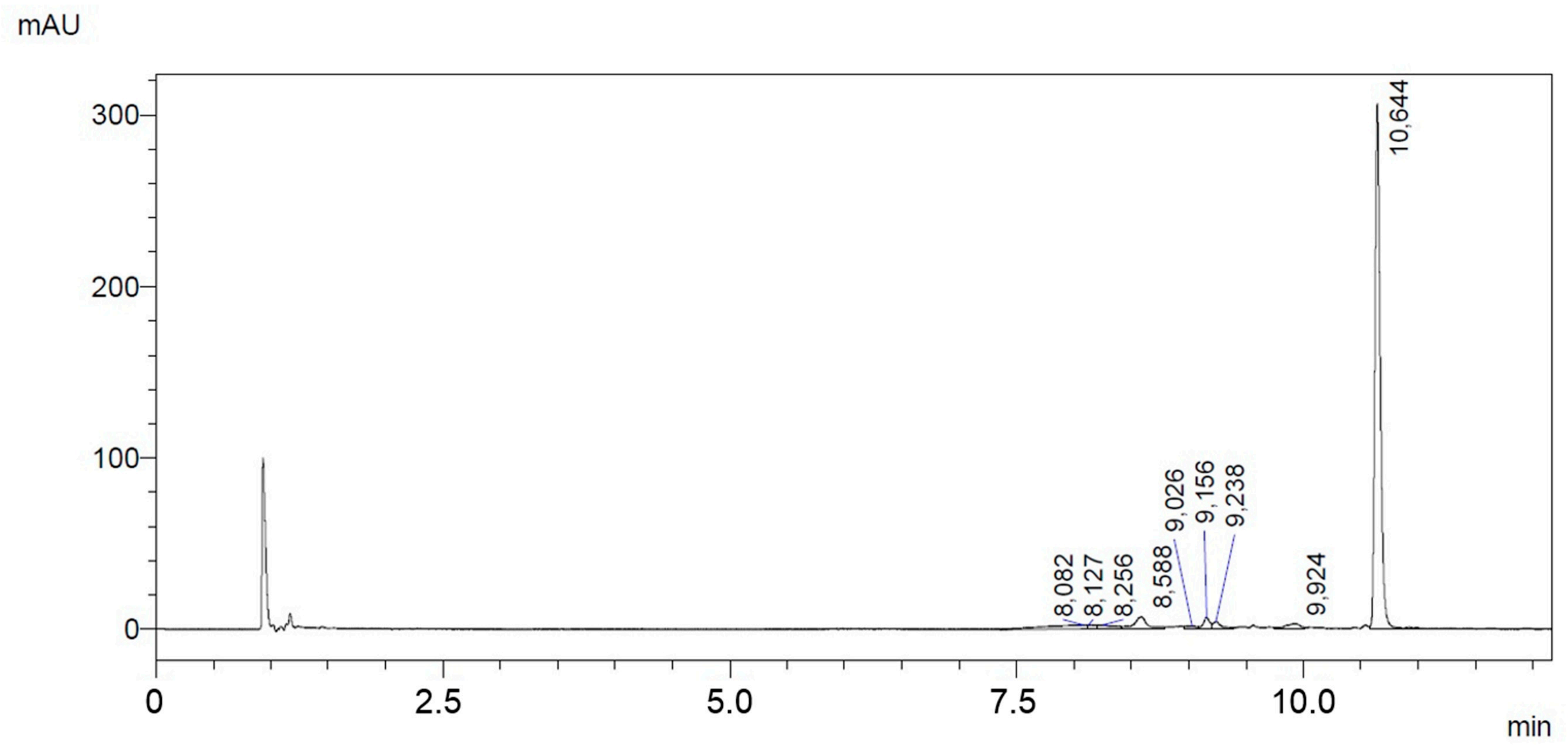

Figure S22. HPLC chromatogram of compound **8**.

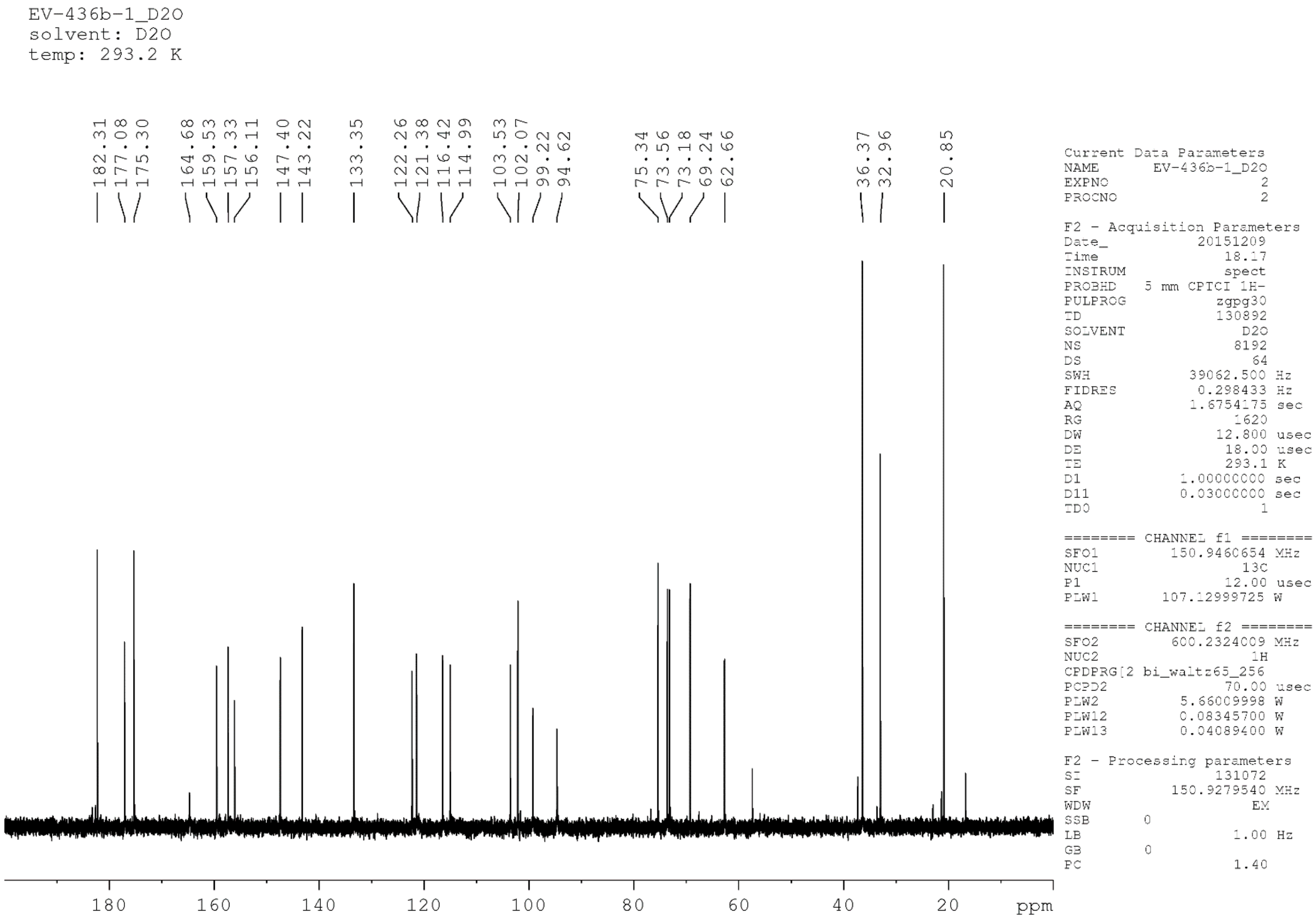

Figure S23. ^{13}C NMR spectrum of compound **9**.

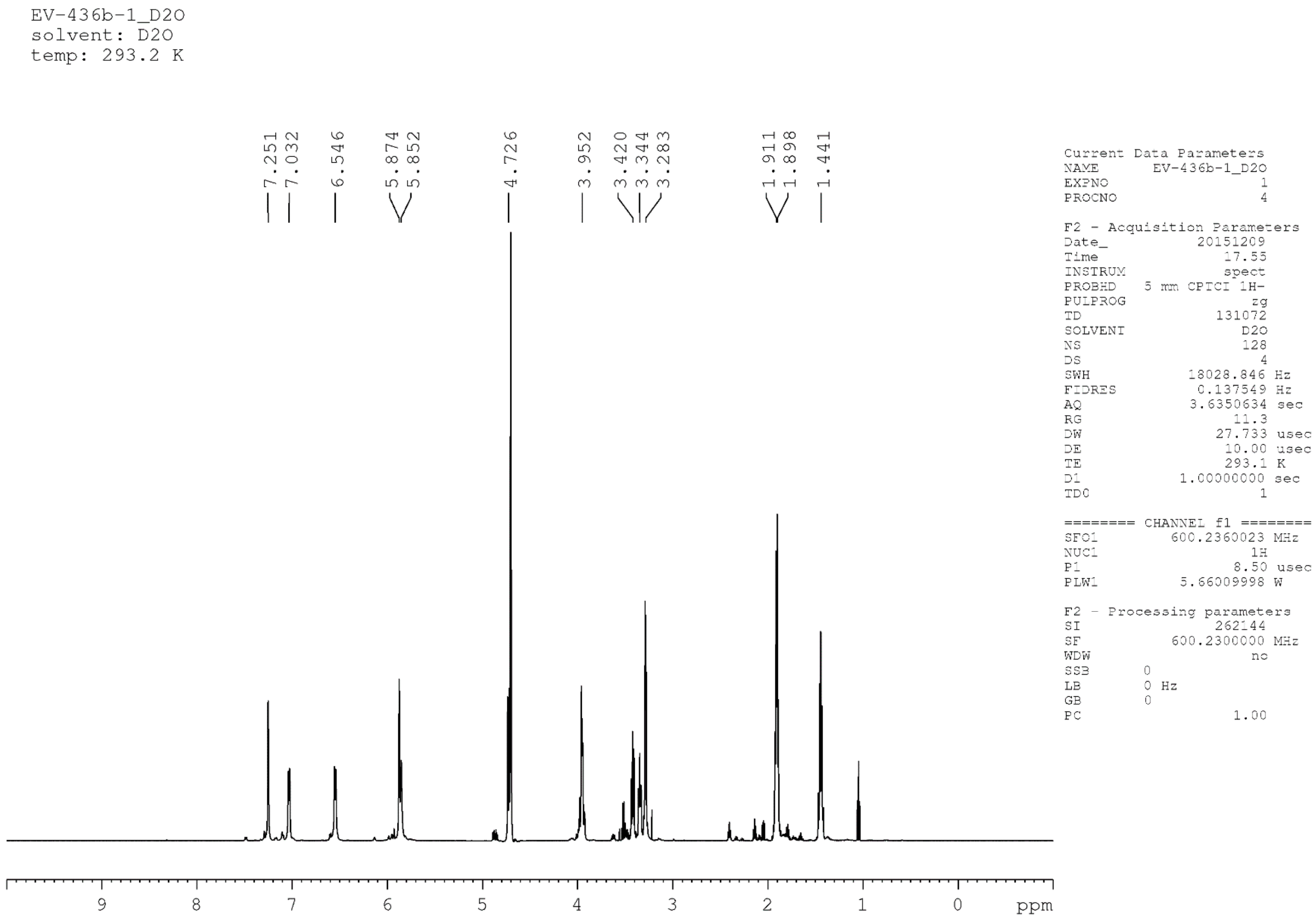

Figure S24. ^{1}H NMR spectrum of compound **9**.

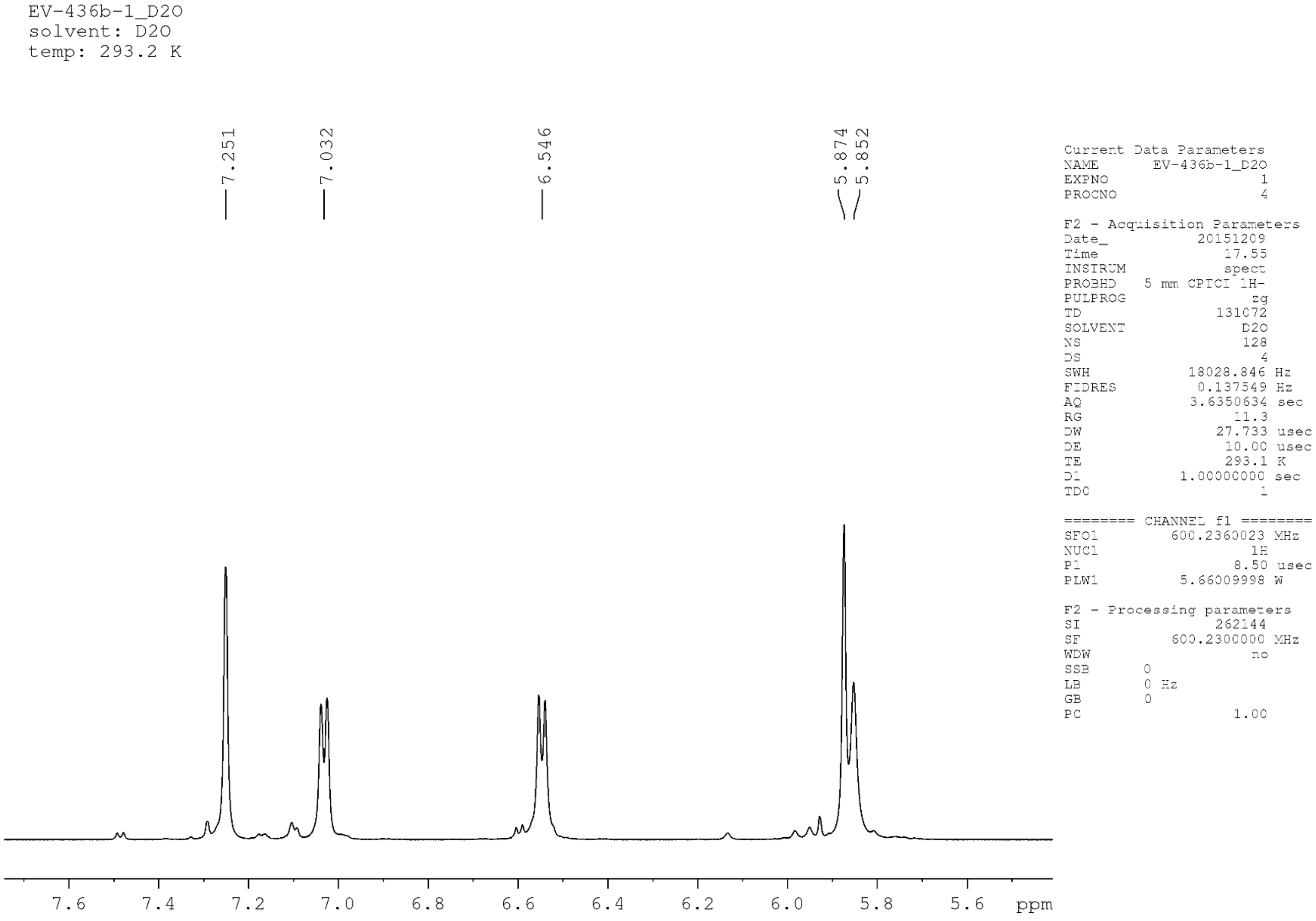

Figure S25. Detail (1/3) of ^{1}H NMR spectrum of compound **9**.

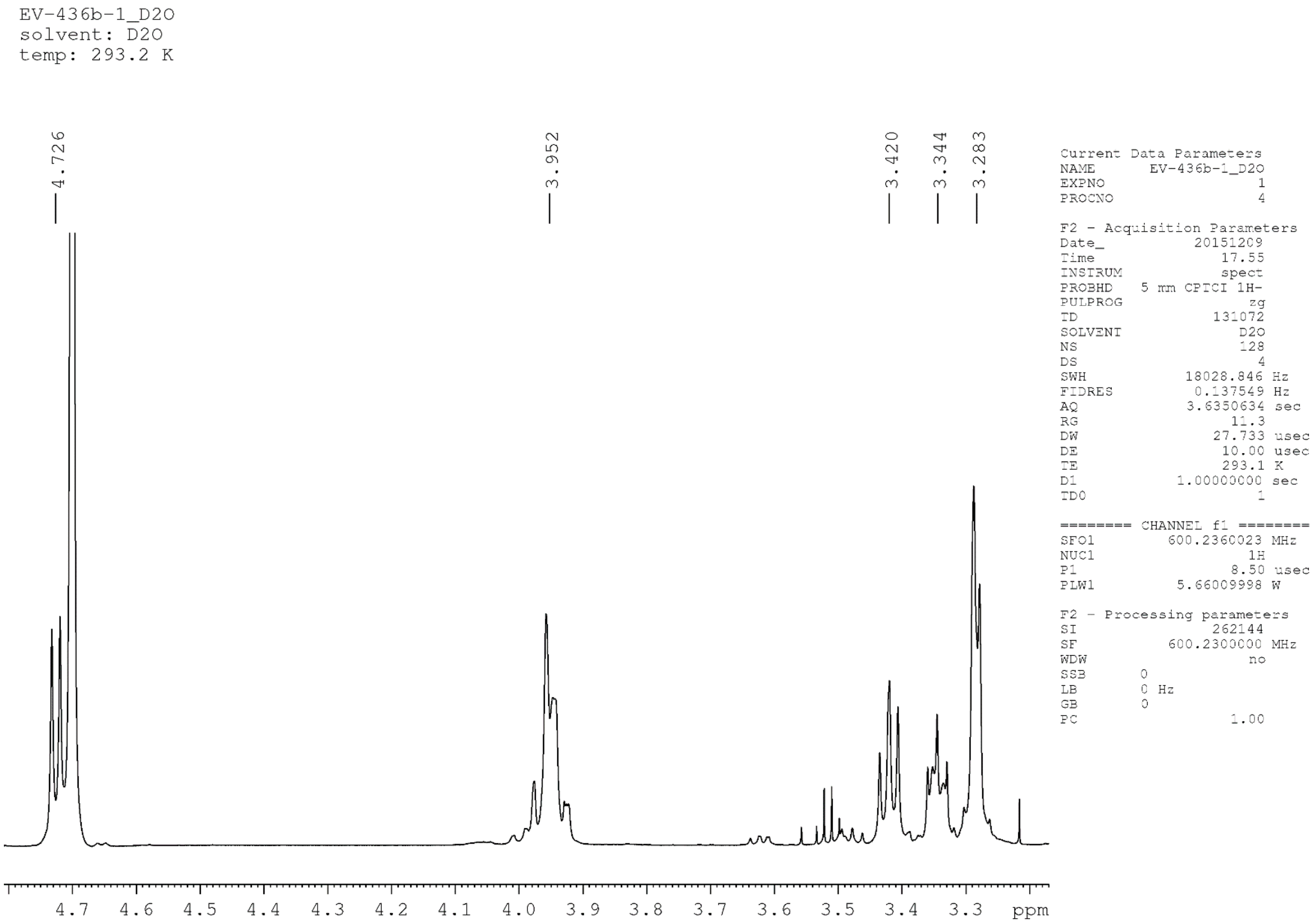

Figure S26. Detail (2/3) of ^{1}H NMR spectrum of compound **9**.

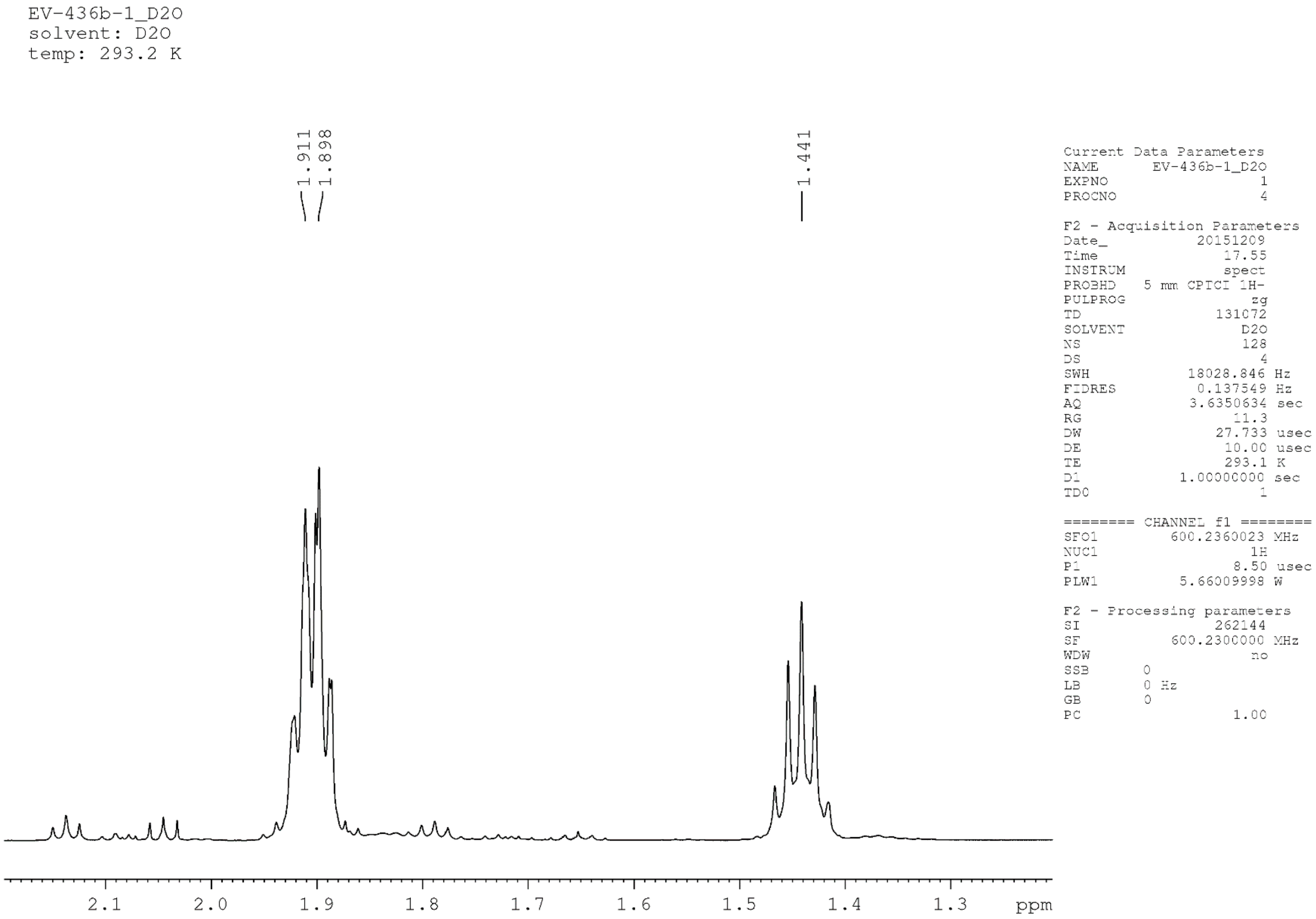

Figure S27. Detail (3/3) of ^{1}H NMR spectrum of compound **9**.

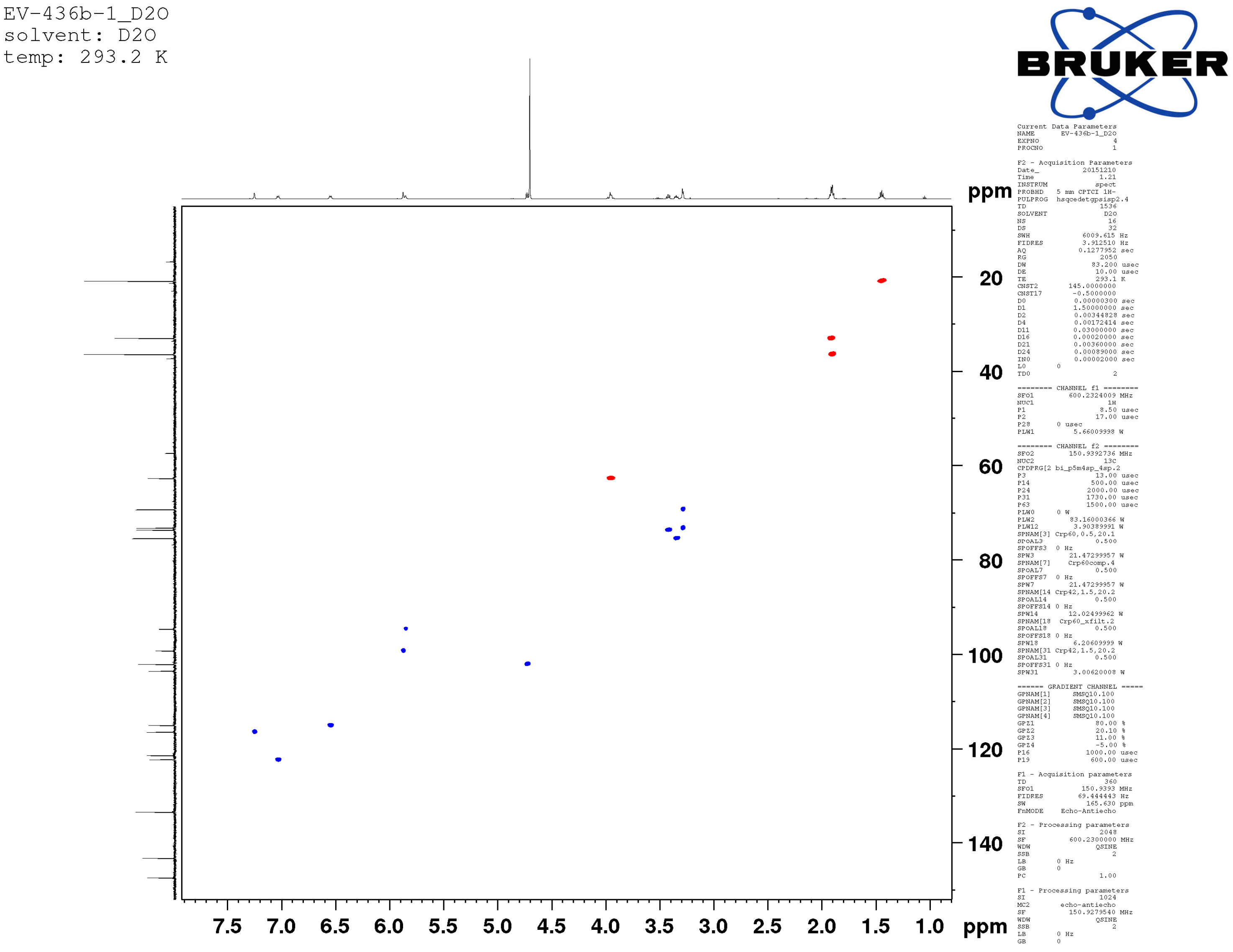

Figure S28. HSQC NMR spectrum of compound **9**.

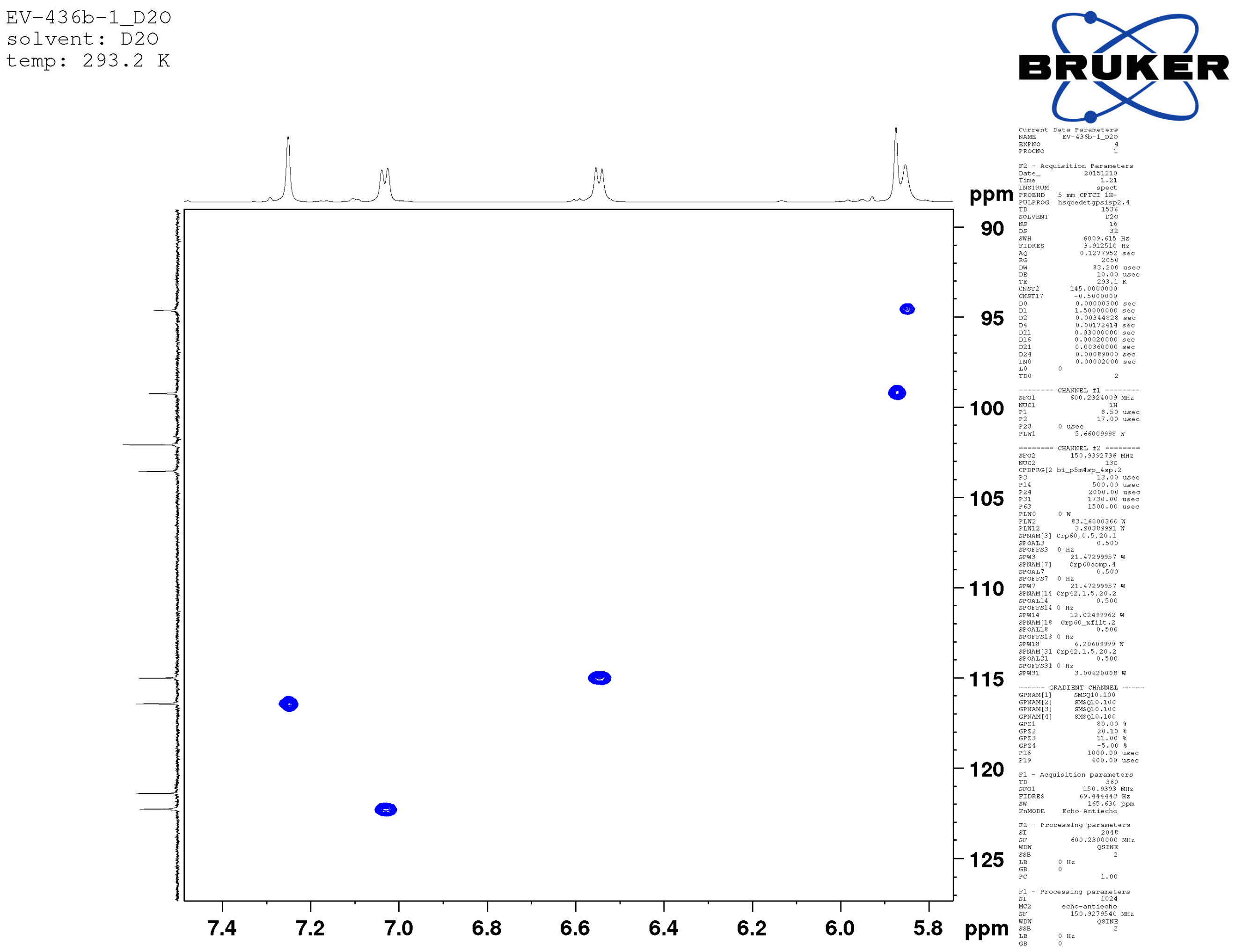

Figure S29. Detail (1/3) of HSQC NMR spectrum of compound **9**.

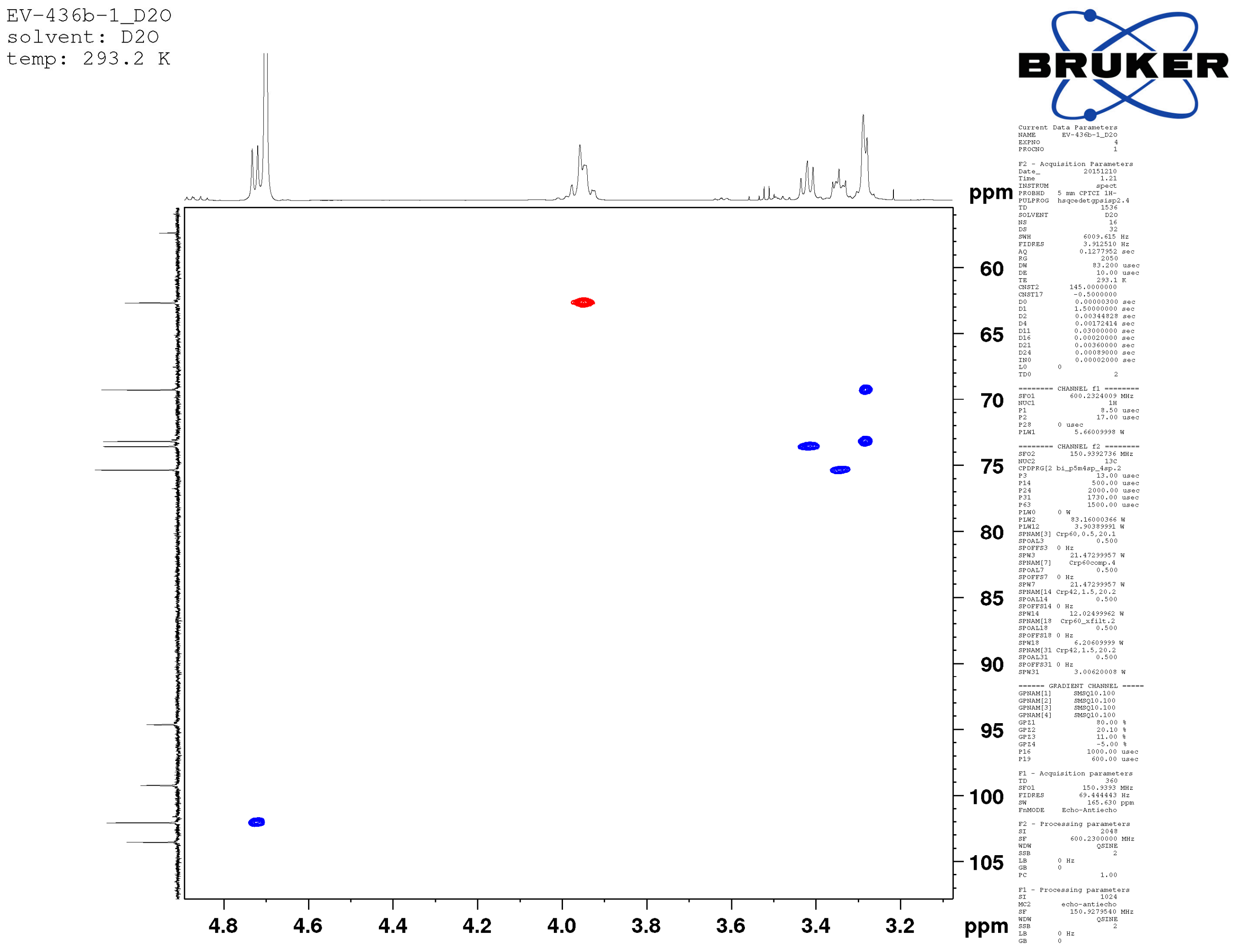

Figure S30. Detail (2/3) of HSQC NMR spectrum of compound **9**.

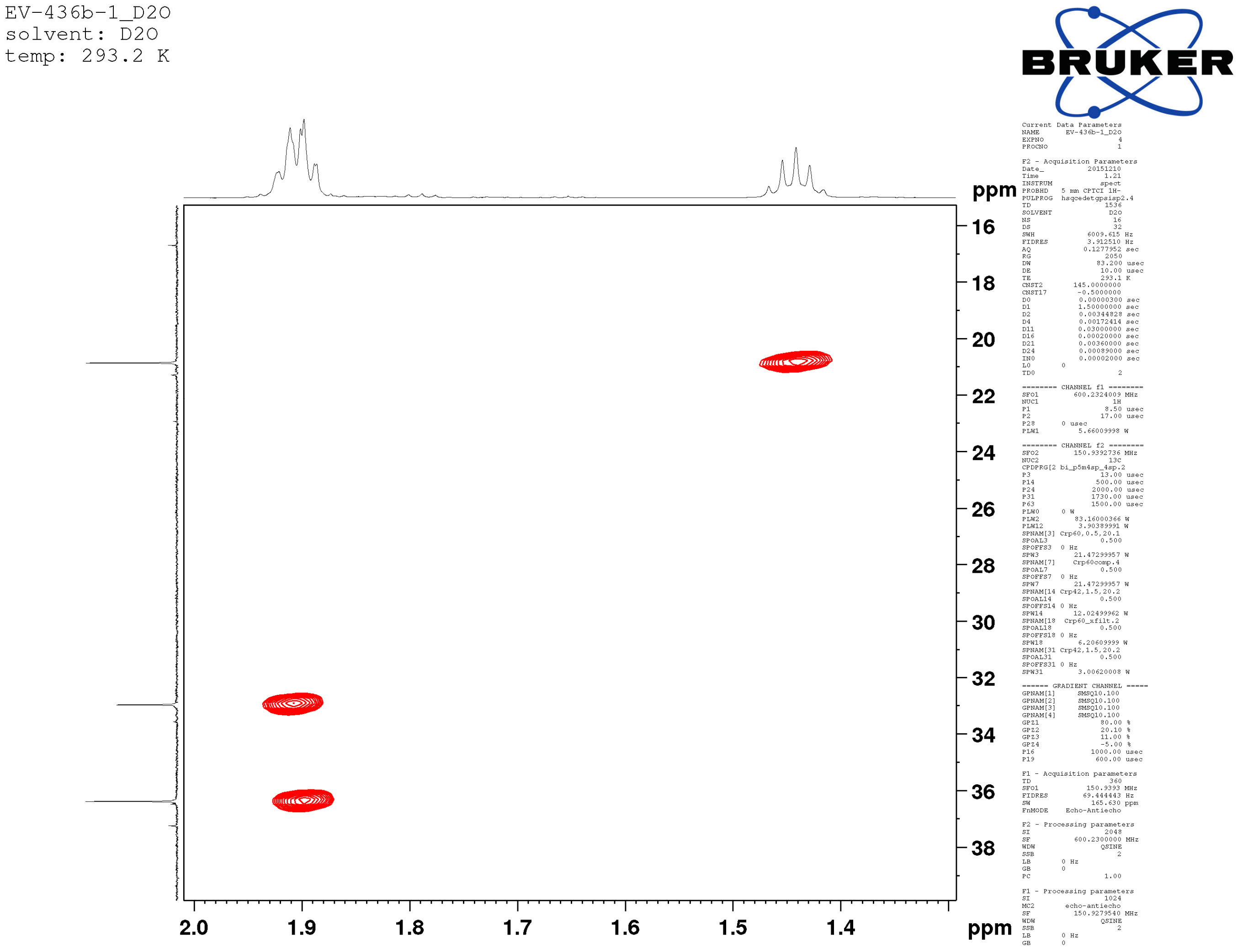

Figure S31. Detail (3/3) of HSQC NMR spectrum of compound **9**.

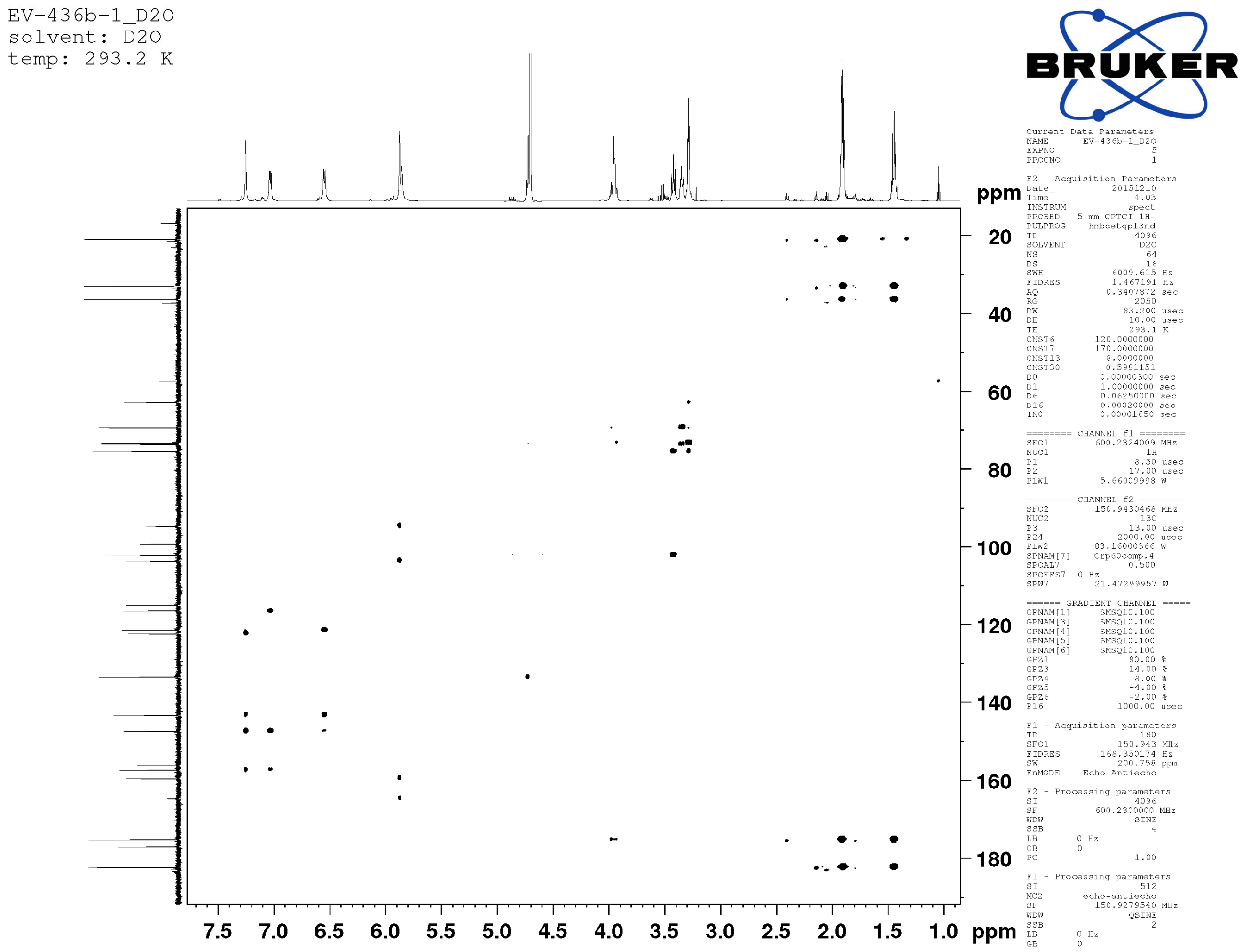

Figure S32. HMBC NMR spectrum of compound **9**.

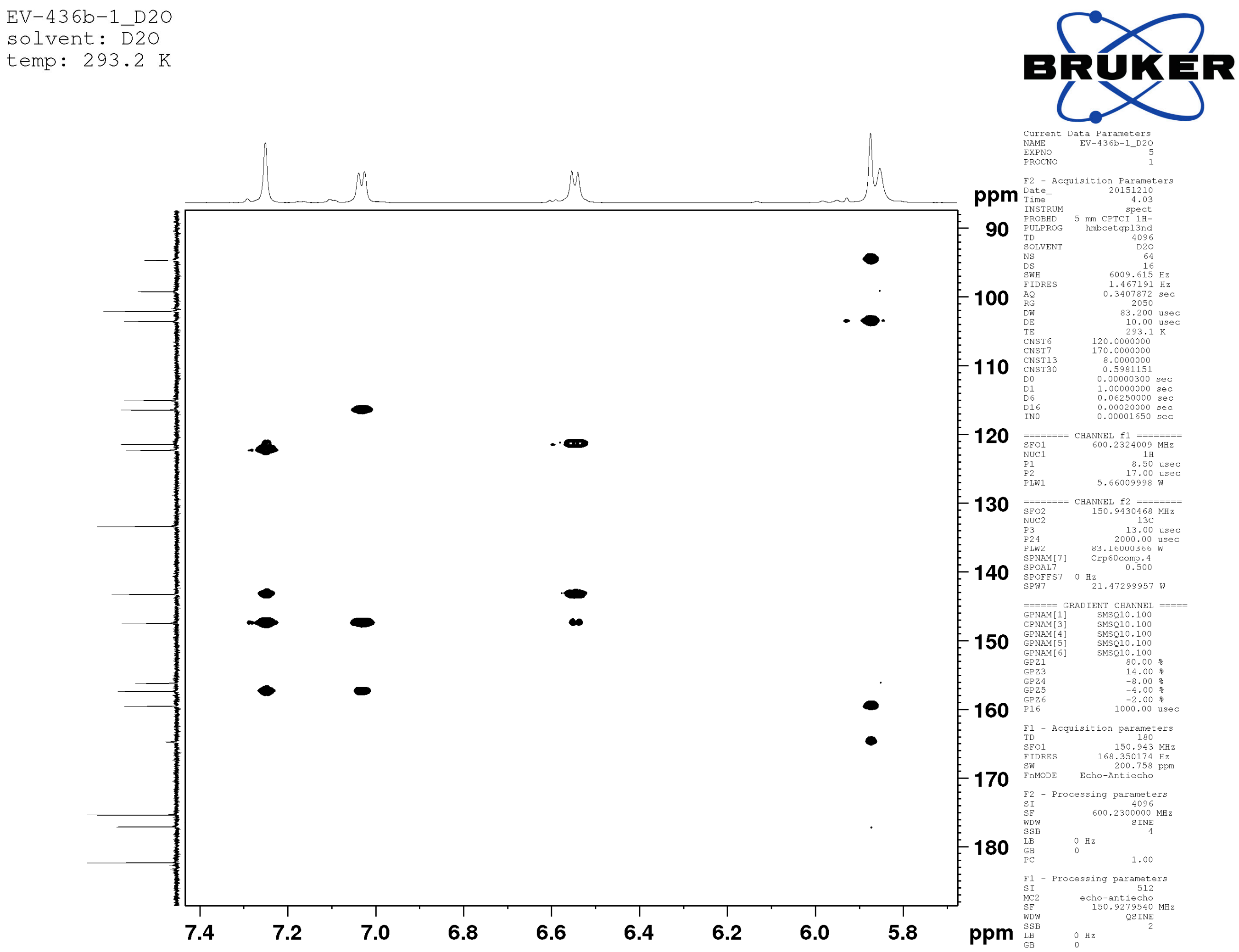

Figure S33. Detail (1/4) of HMBC NMR spectrum of compound **9**.

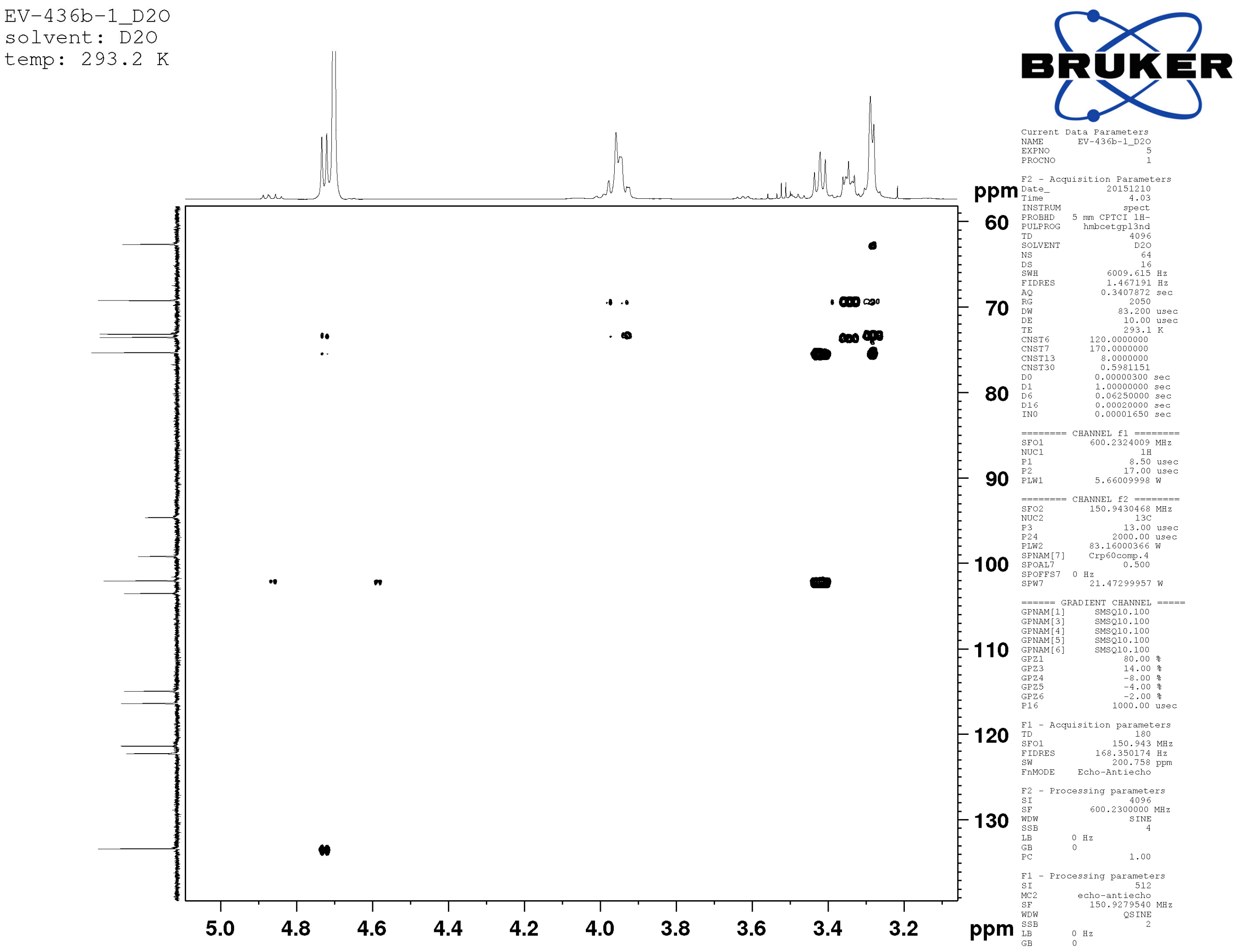

Figure S34. Detail (2/4) of HMBC NMR spectrum of compound **9**.

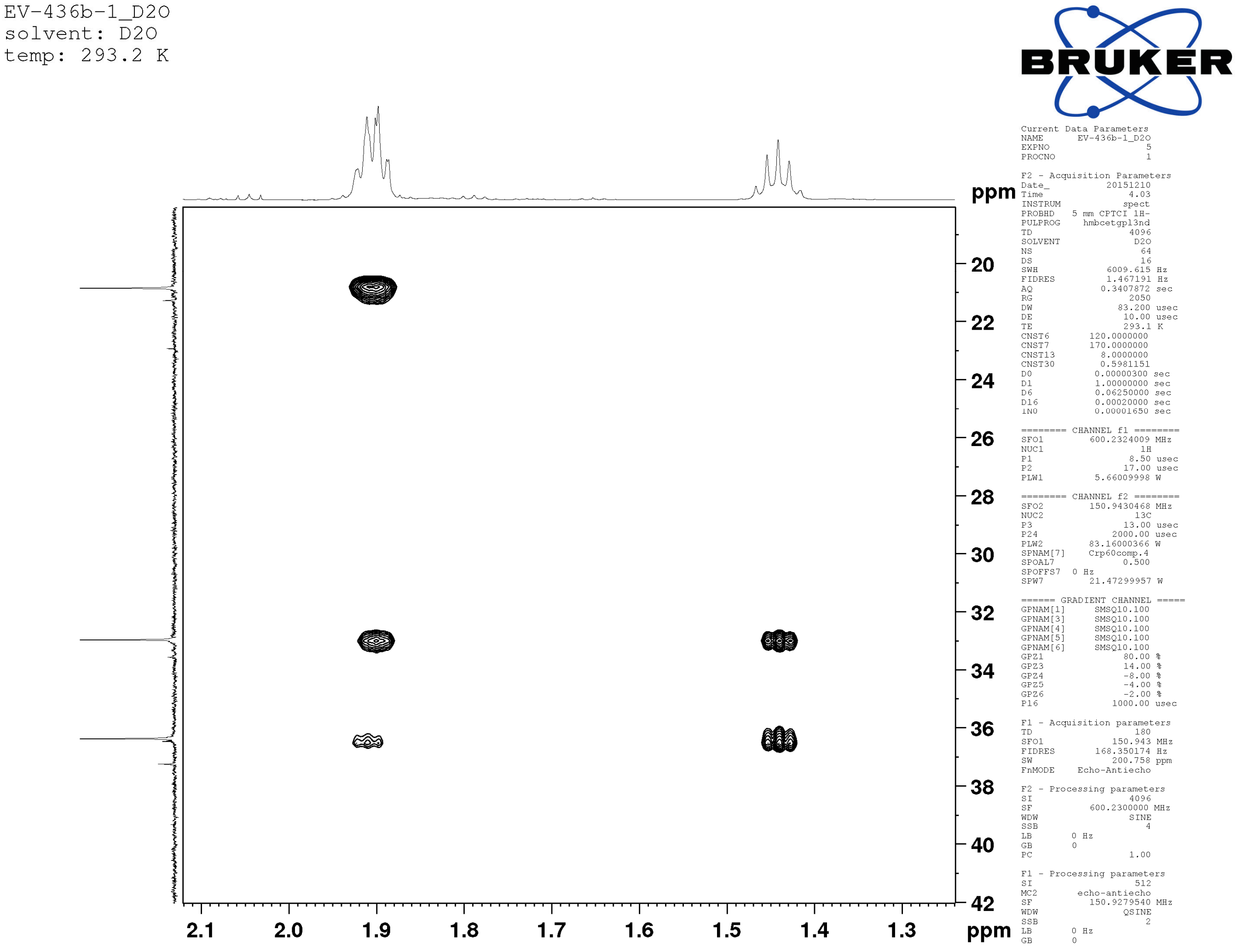

Figure S35. Detail (3/4) of HMBC NMR spectrum of compound **9**.

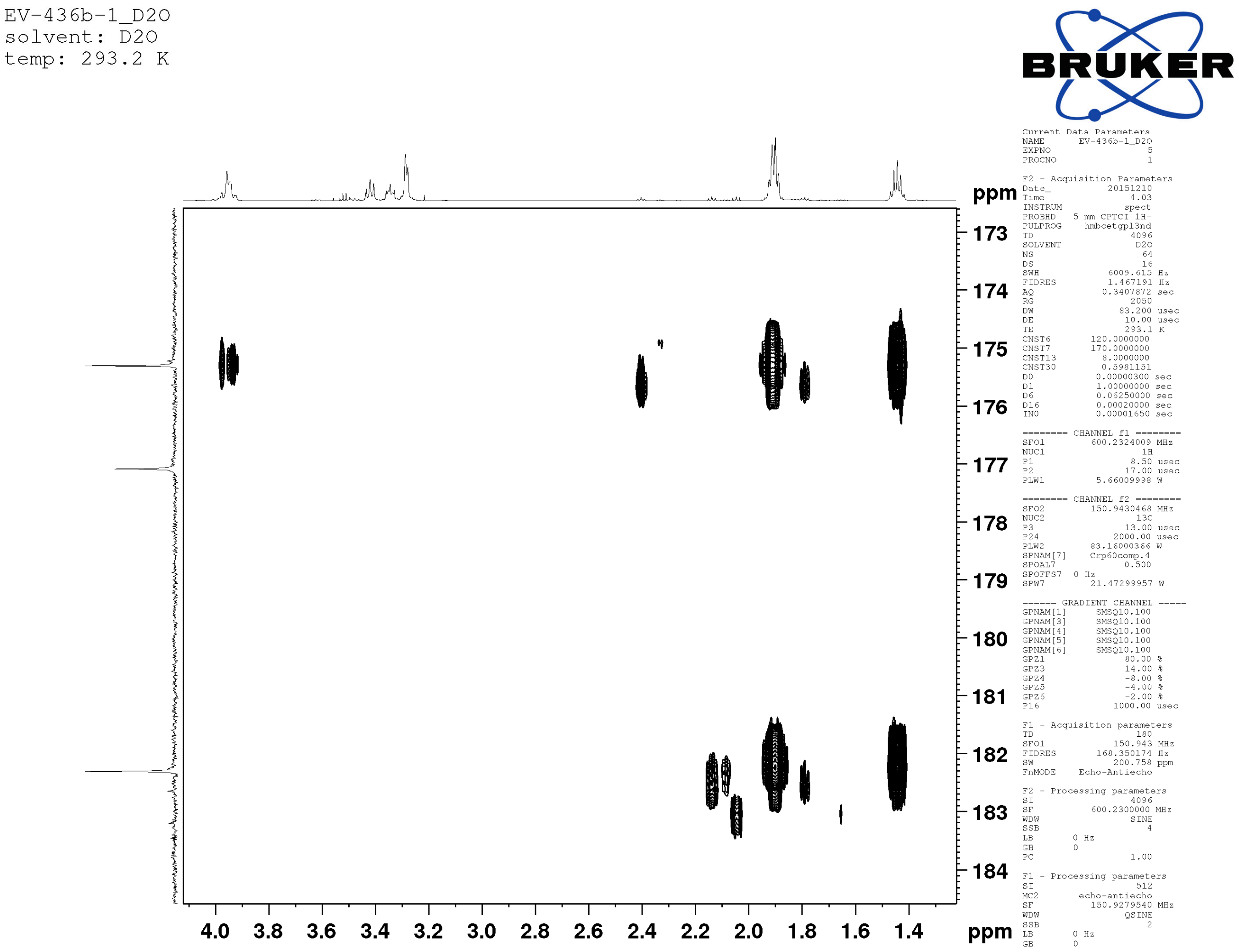

Figure S36. Detail (4/4) of HMBC NMR spectrum of compound **9**.

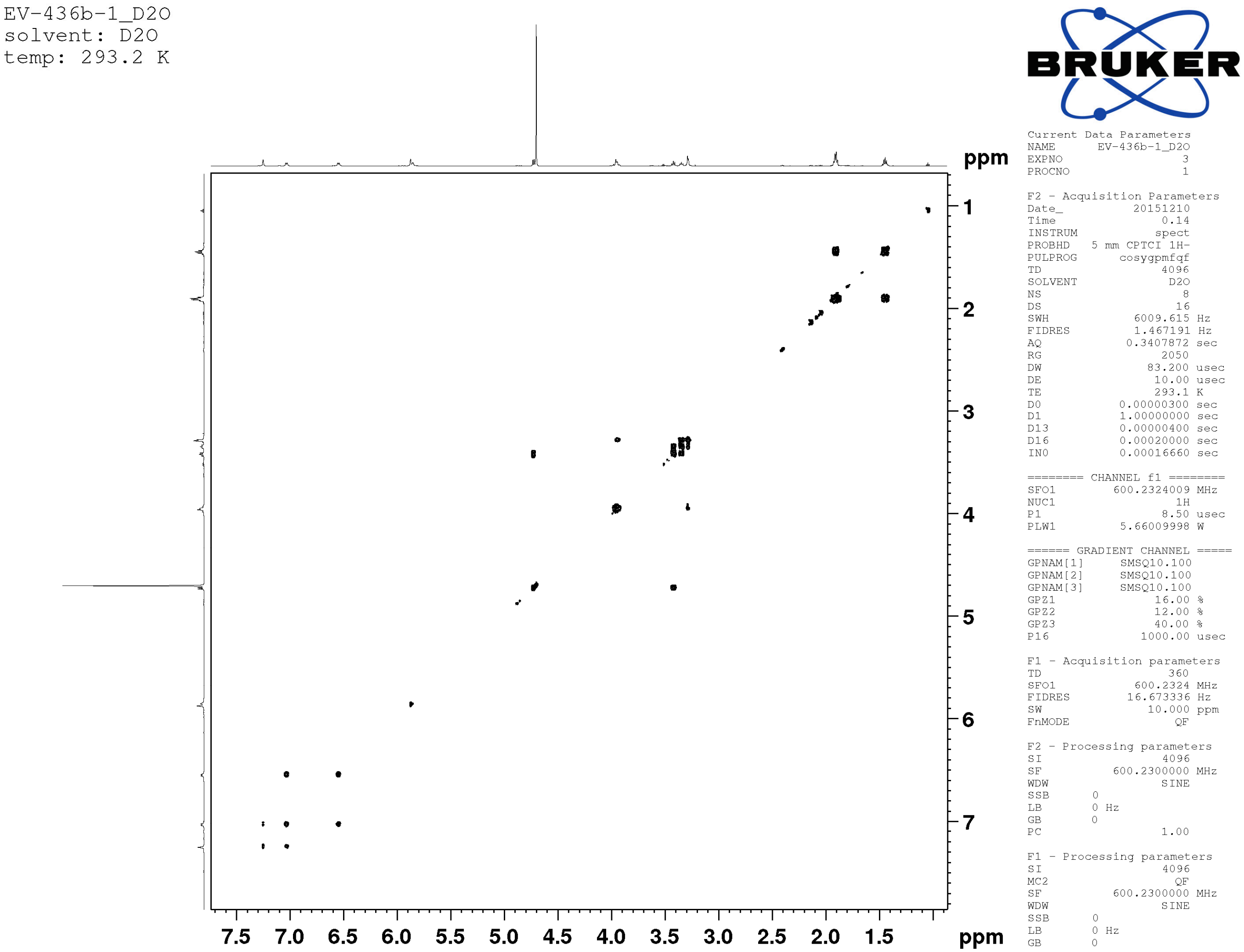

Figure S37. COSY NMR spectrum of compound **9**.

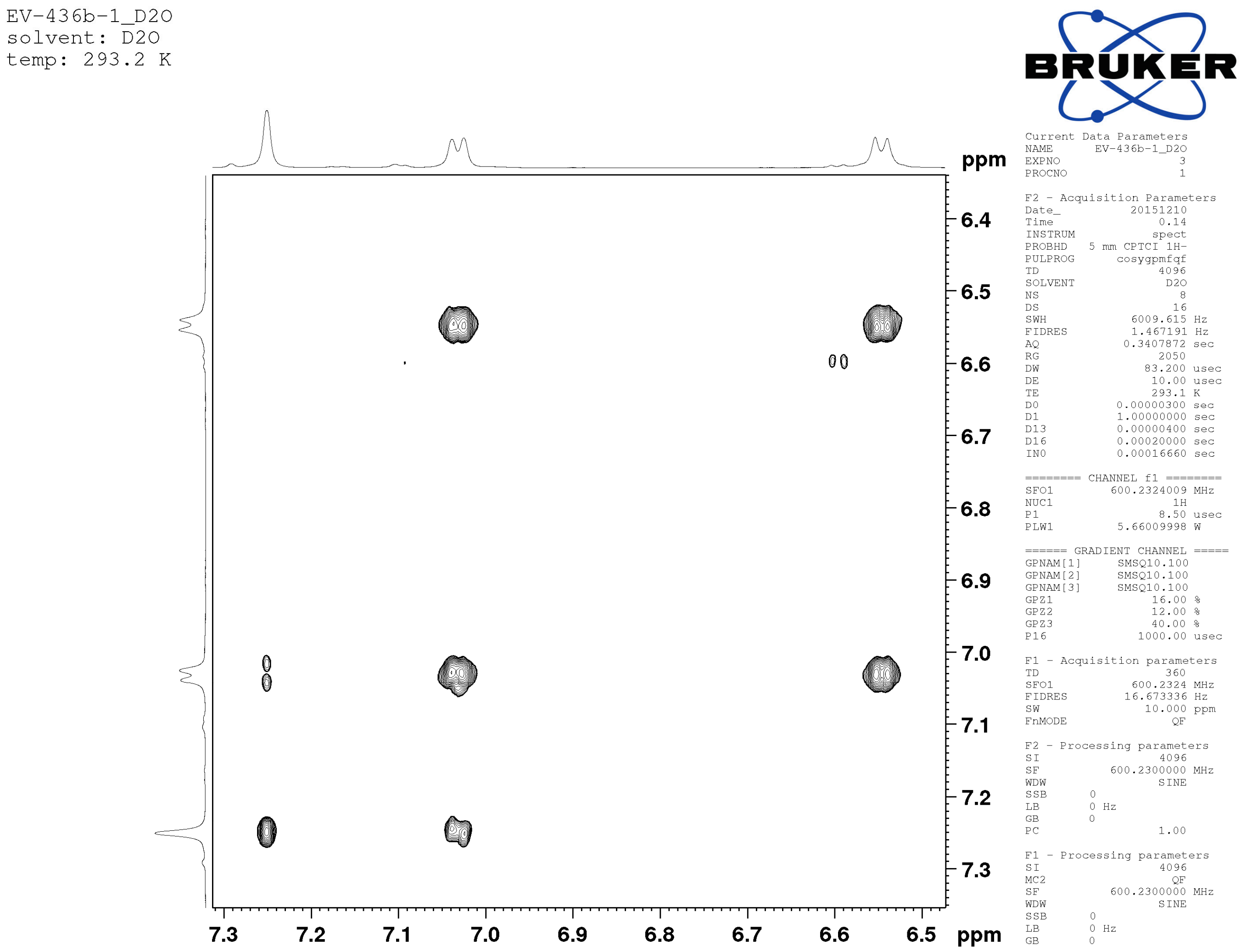

Figure S38. Detail (1/3) of COSY NMR spectrum of compound **9**.

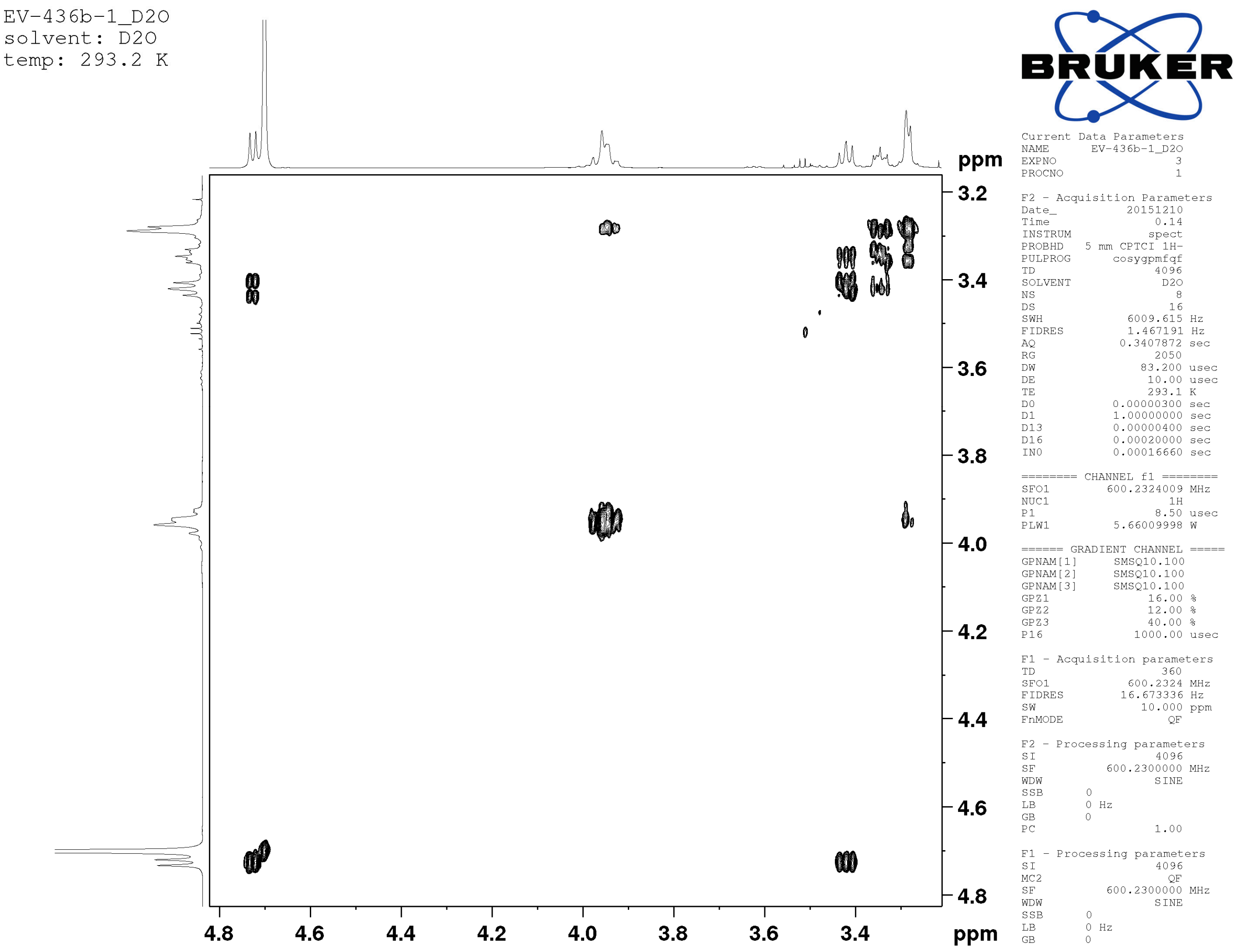

Figure S39. Detail (2/3) of COSY NMR spectrum of compound **9**.

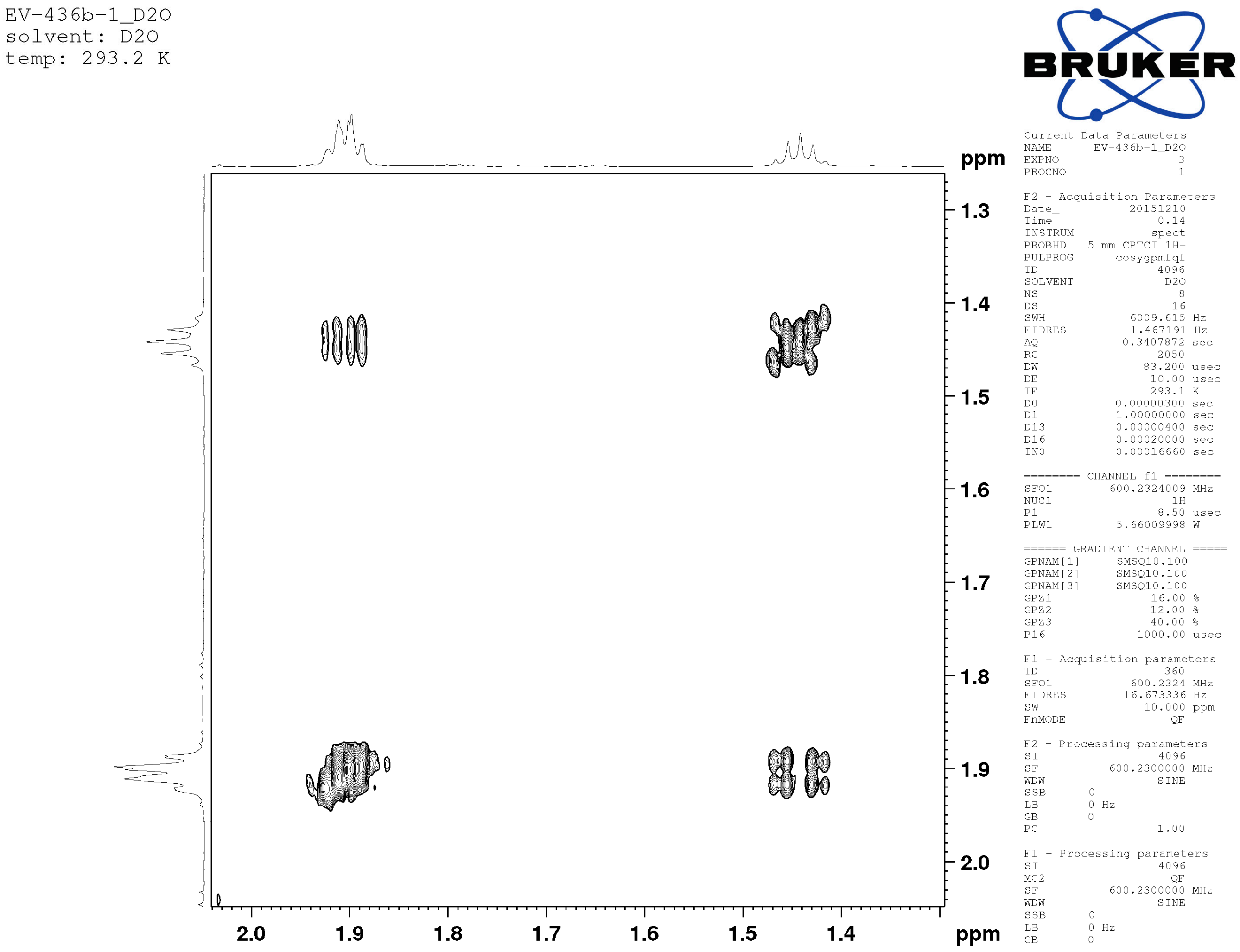

Figure S40. Detail (3/3) of COSY NMR spectrum of compound **9**.

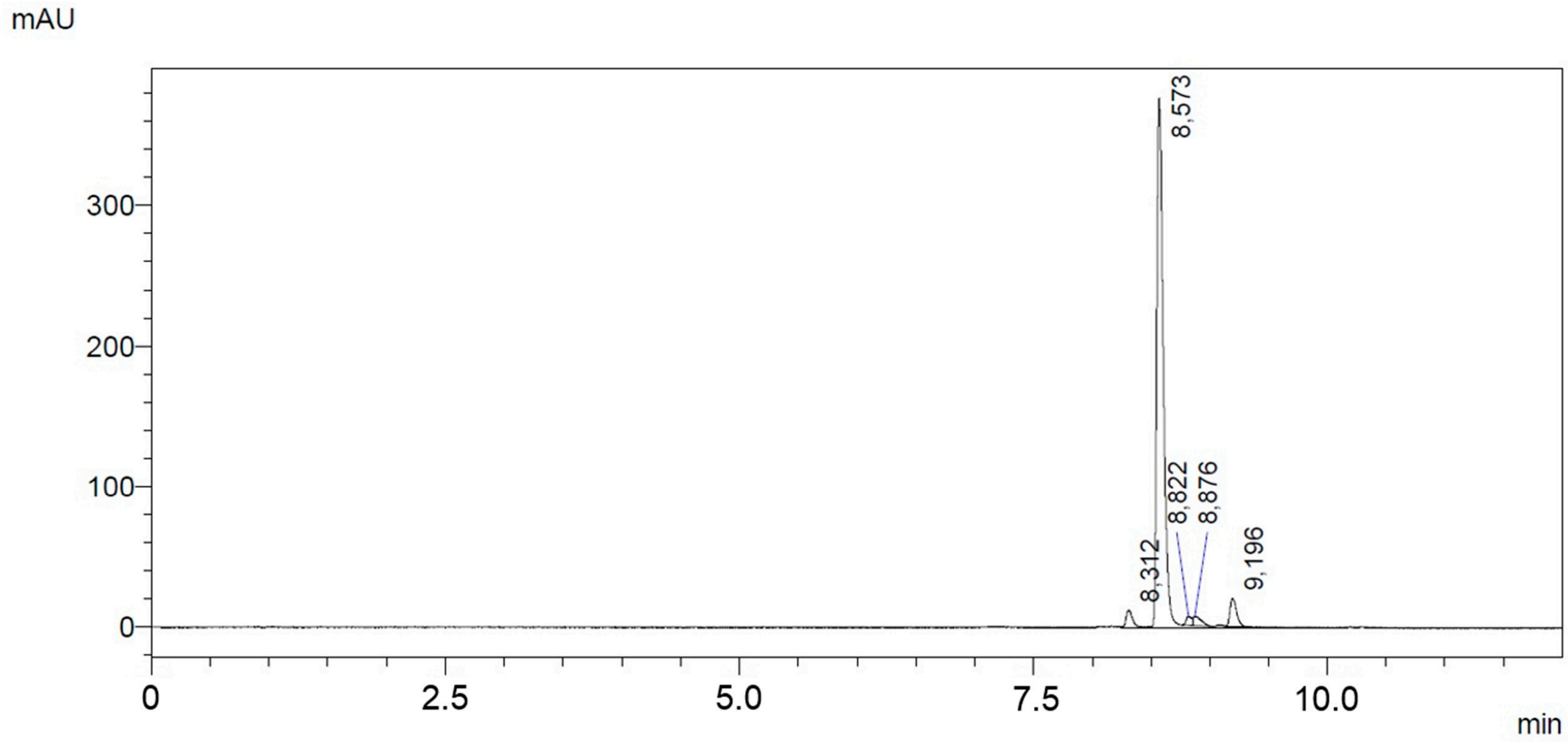

Figure S41. HPLC chromatogram of compound **9**.

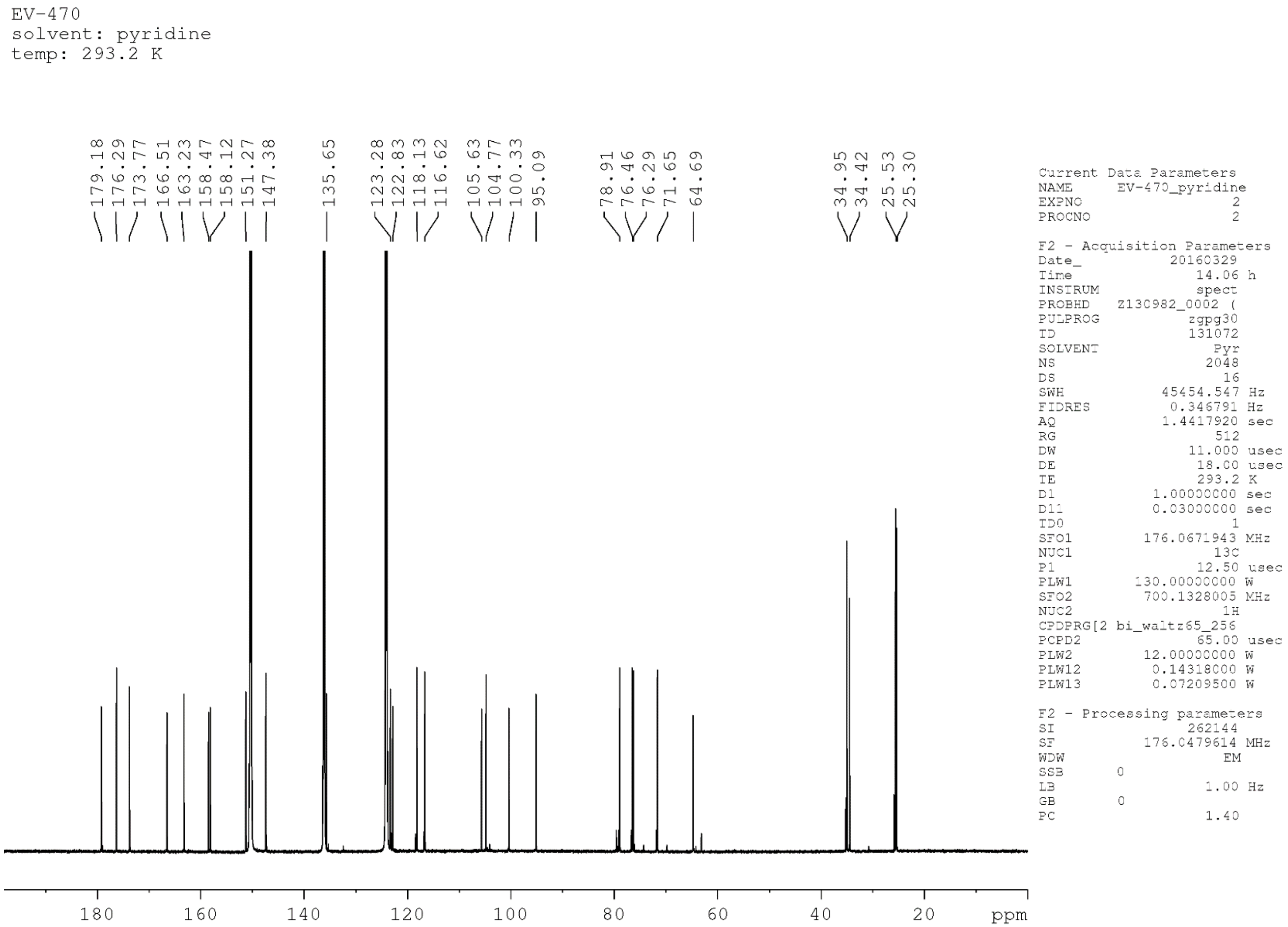

Figure S42. ^{13}C NMR spectrum of compound **10**.

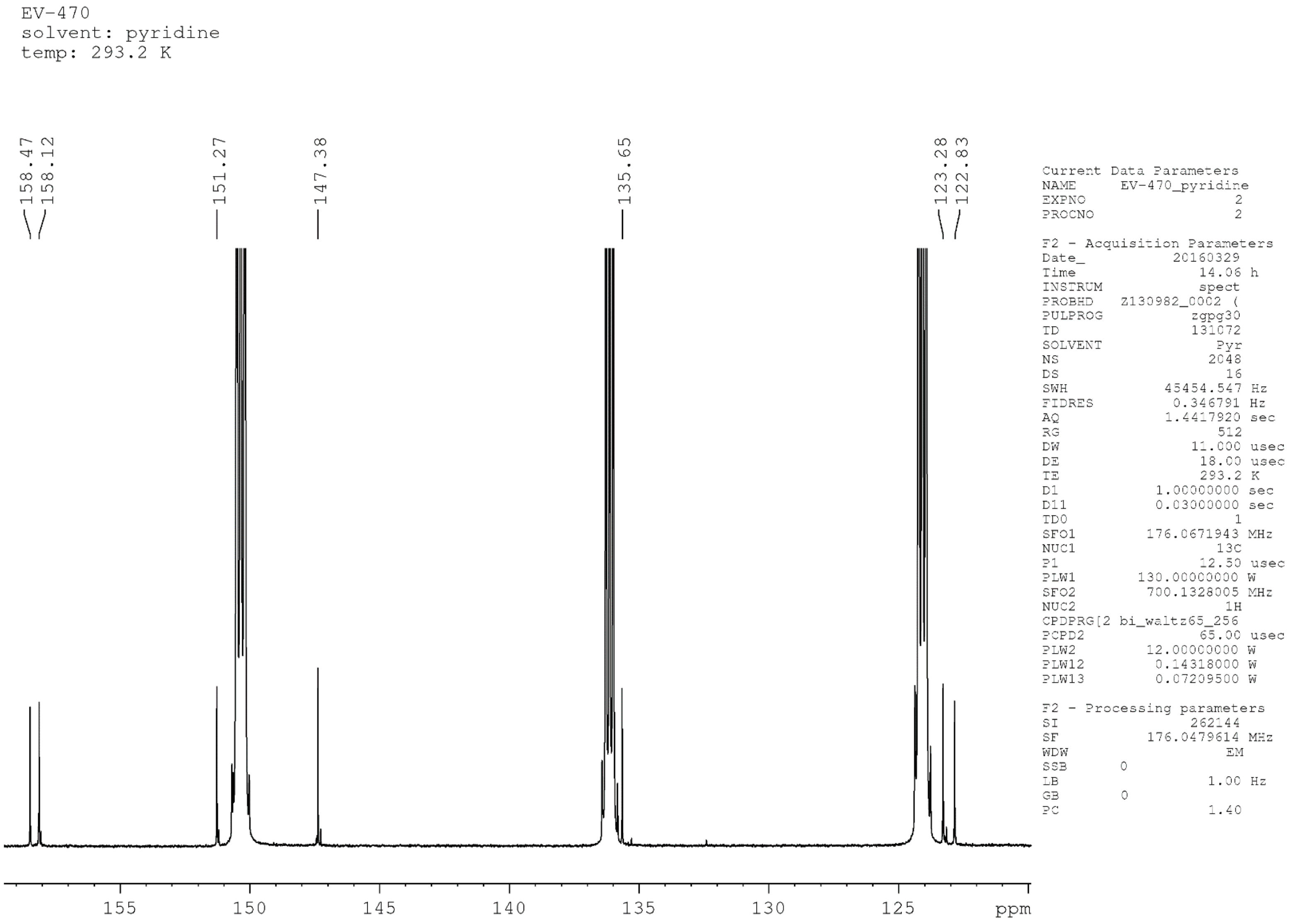

Figure S43. Detail (1/3) of ^{13}C NMR spectrum of compound **10**.

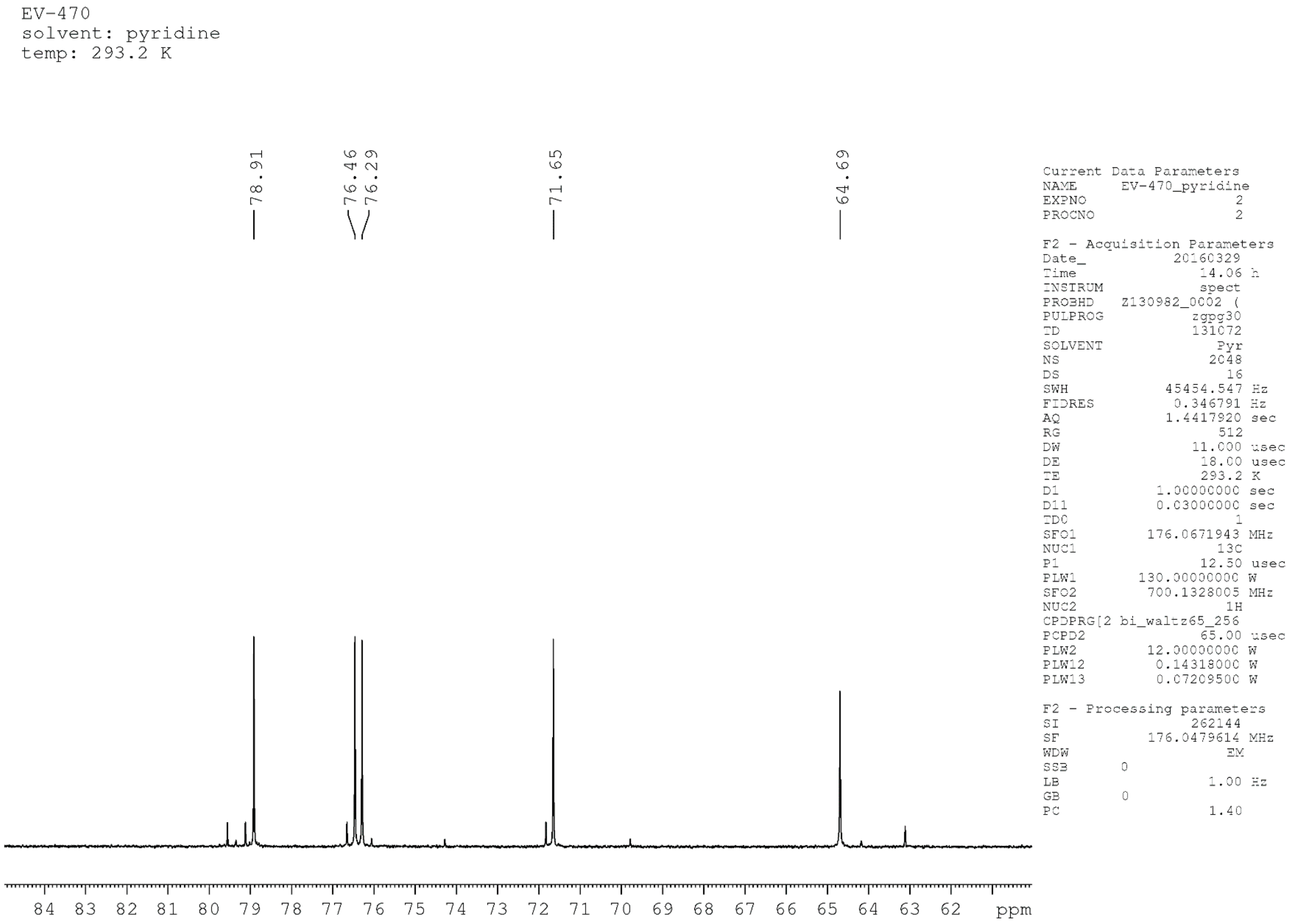

Figure S44. Detail (2/3) of ^{13}C NMR spectrum of compound **10**.

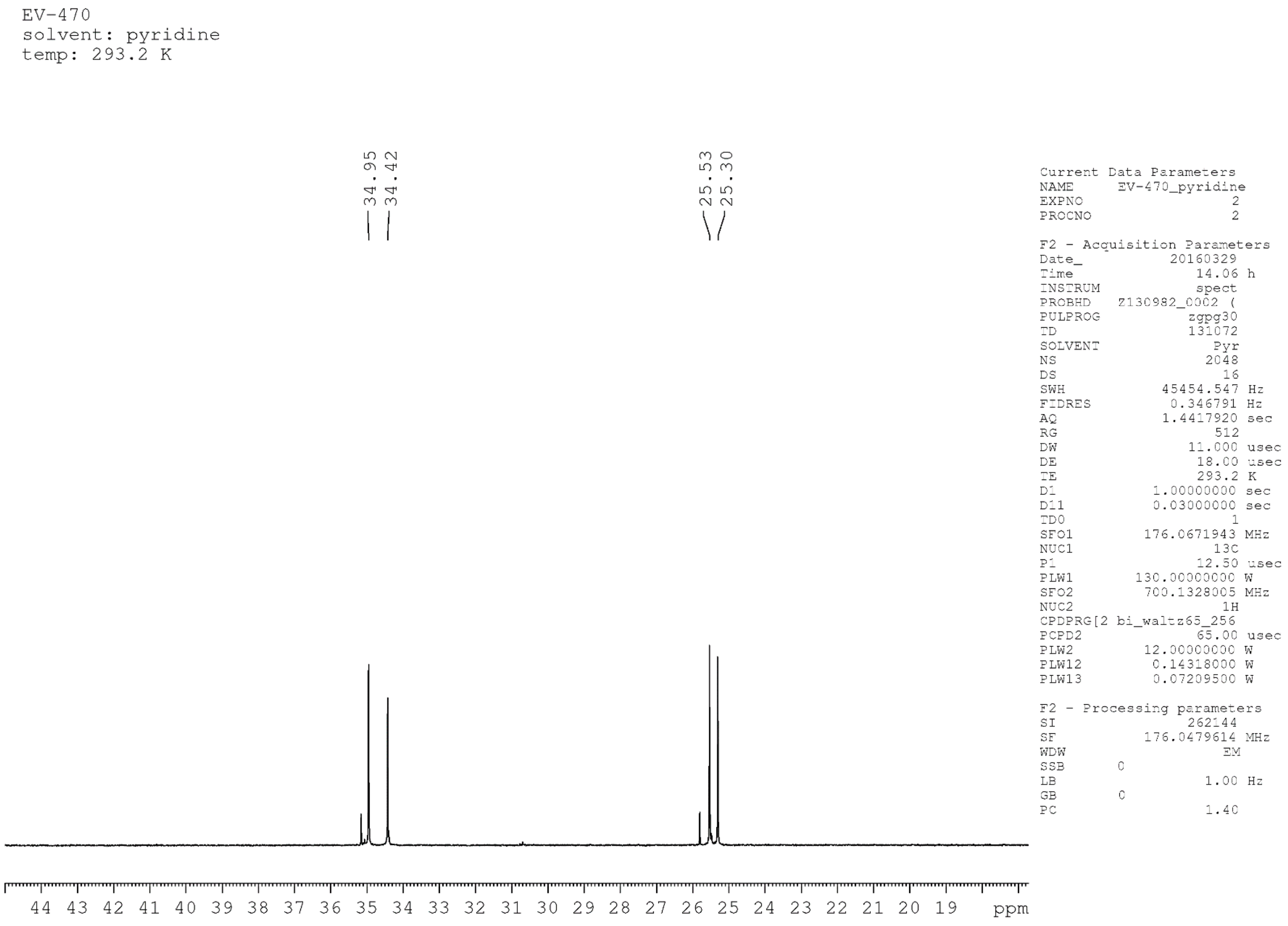

Figure S45. Detail (3/3) of ^{13}C NMR spectrum of compound **10**.

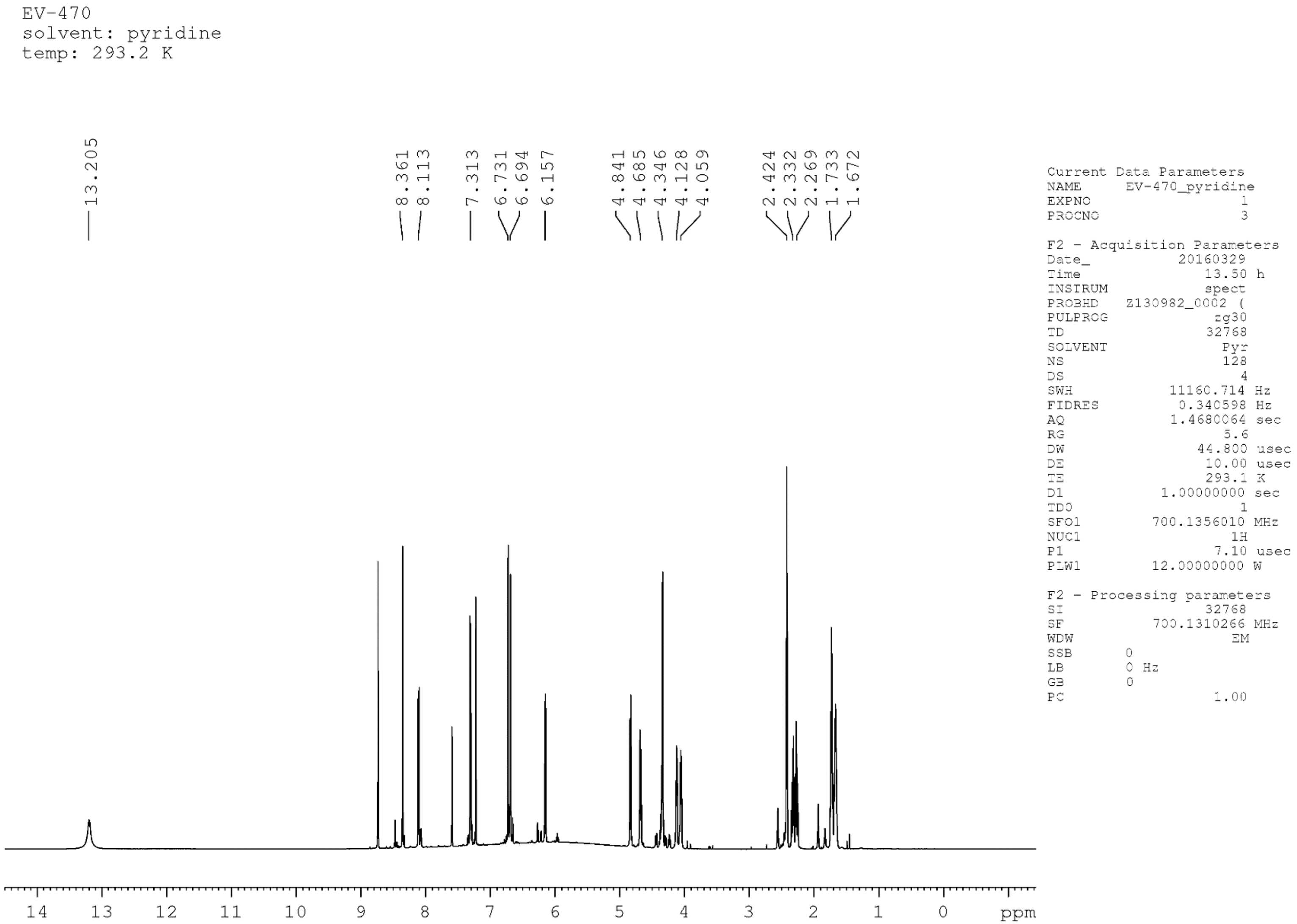

Figure S46. ^{1}H NMR spectrum of compound **10**.

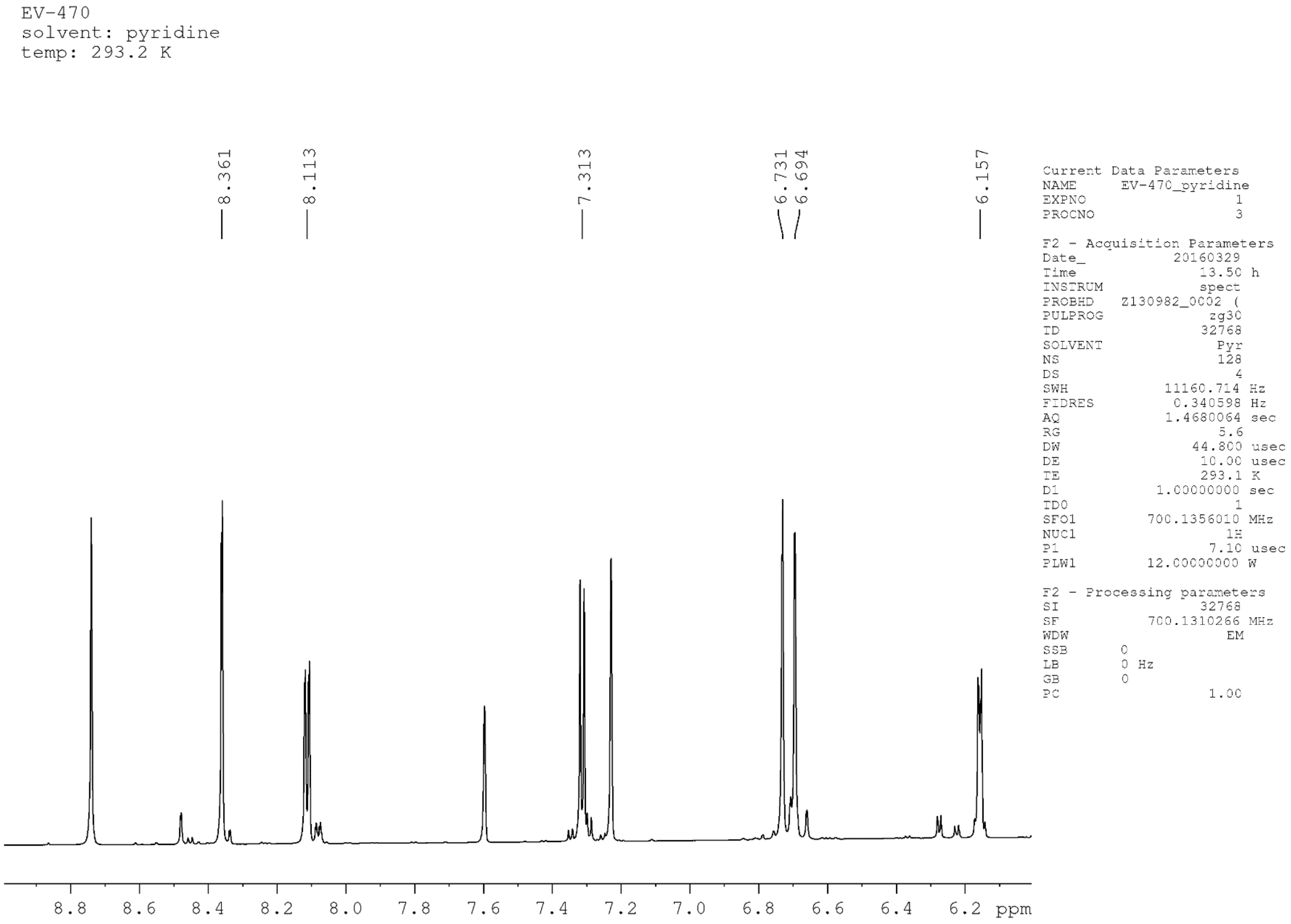

Figure S47. Detail (1/3) of ¹H NMR spectrum of compound **10**.

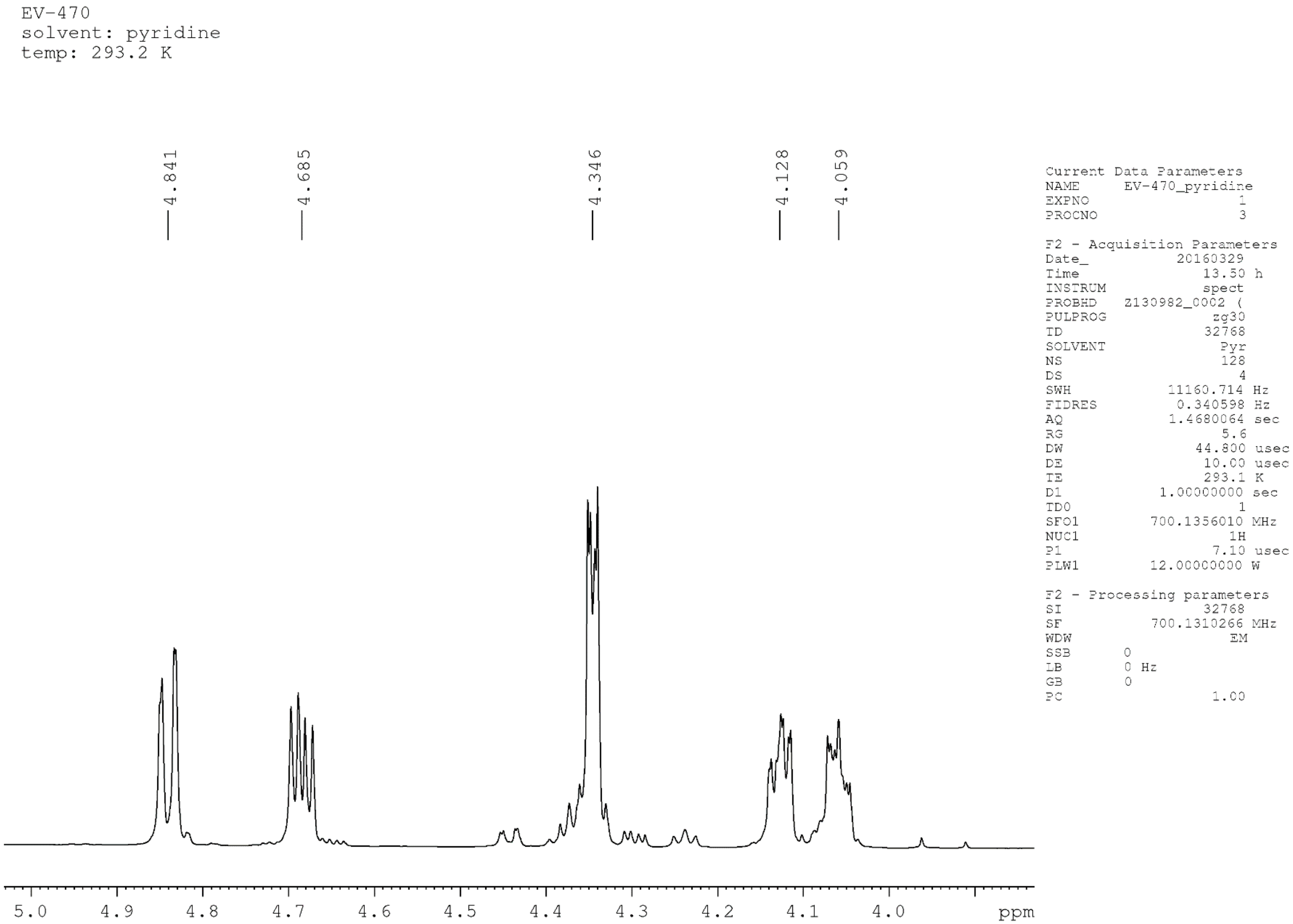

Figure S48. Detail (2/3) of ^{1}H NMR spectrum of compound **10**.

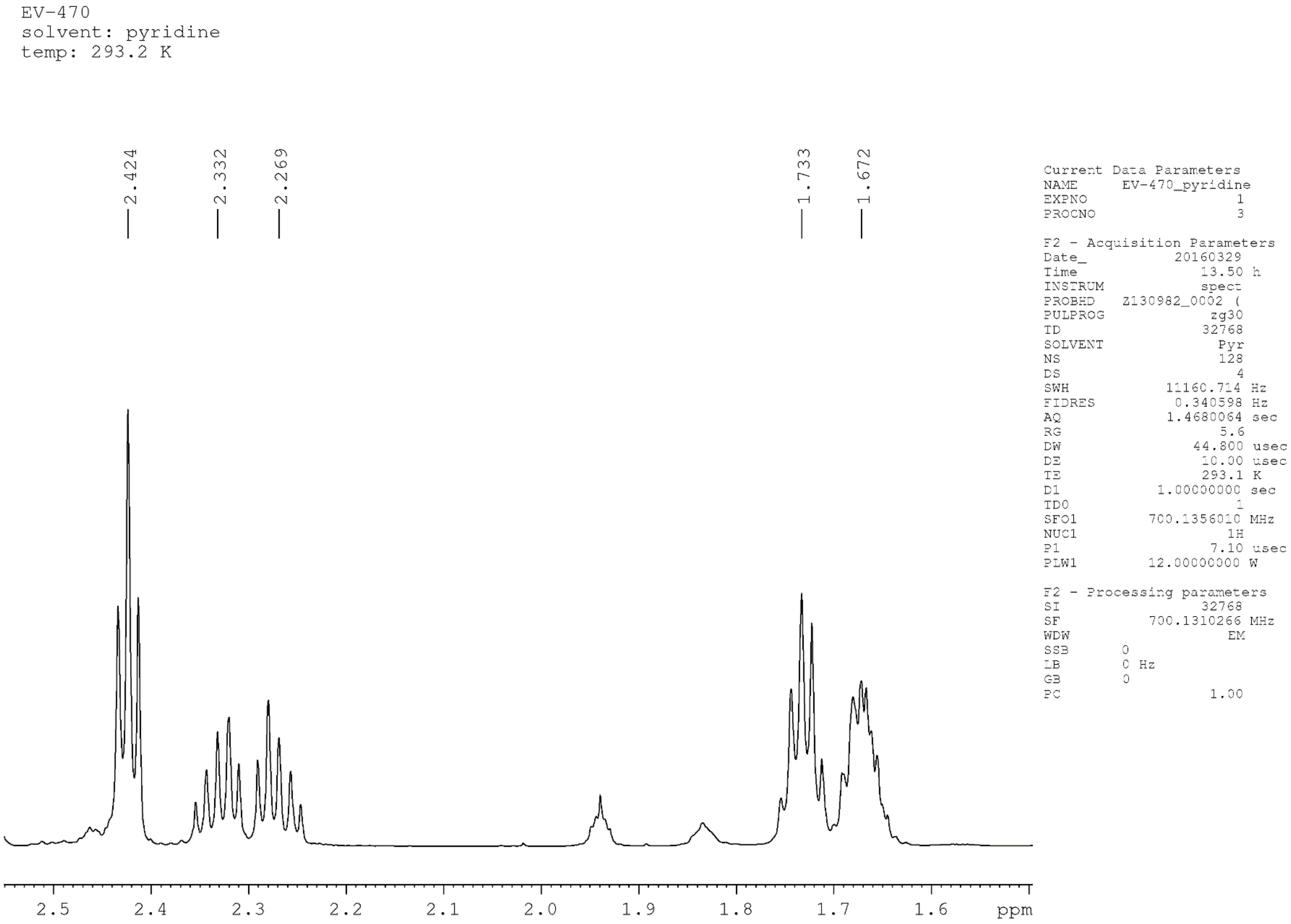

Figure S49. Detail (3/3) of ^{1}H NMR spectrum of compound **10**.

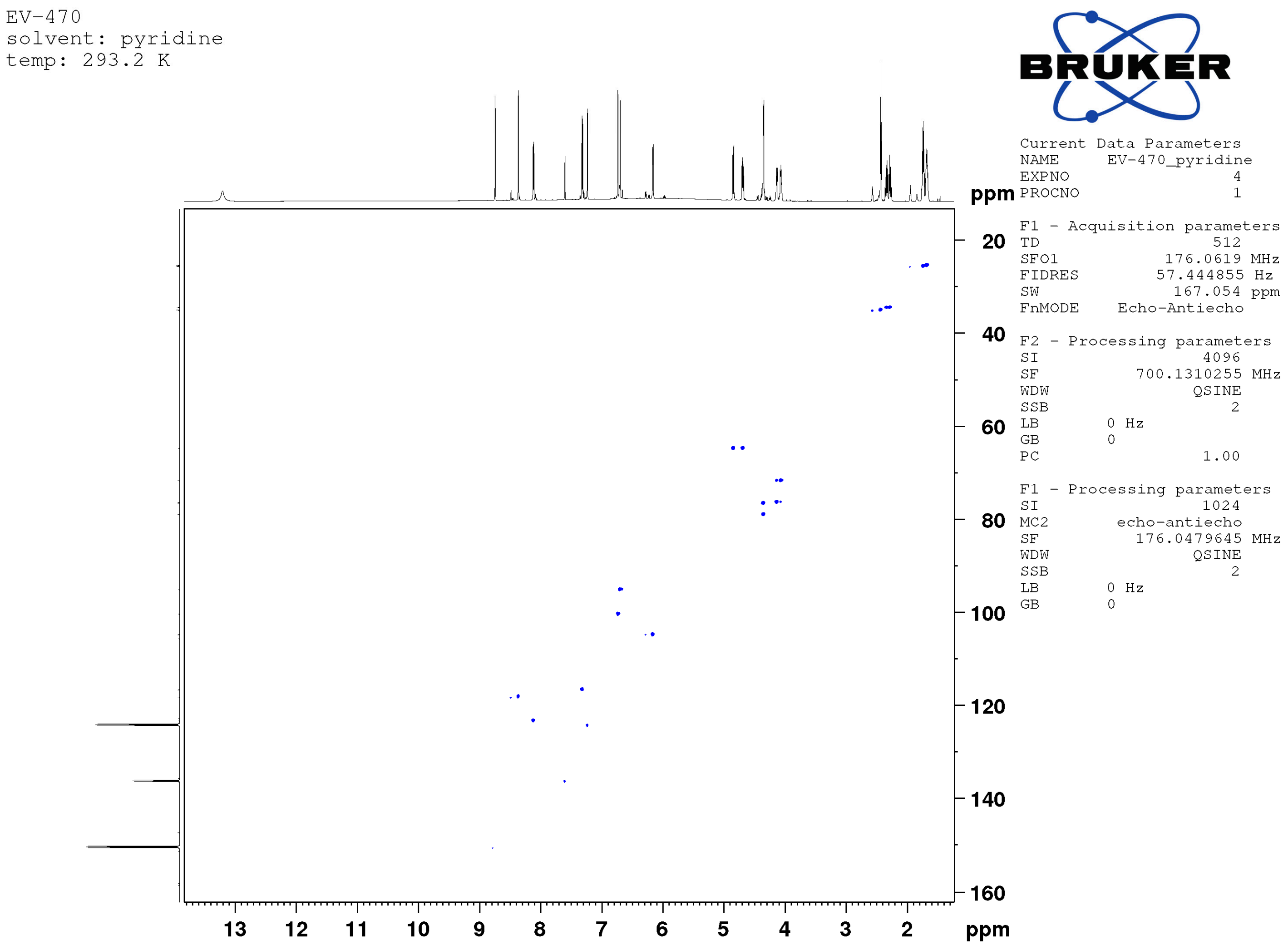

Figure S50. HSQC NMR spectrum of compound **10**.

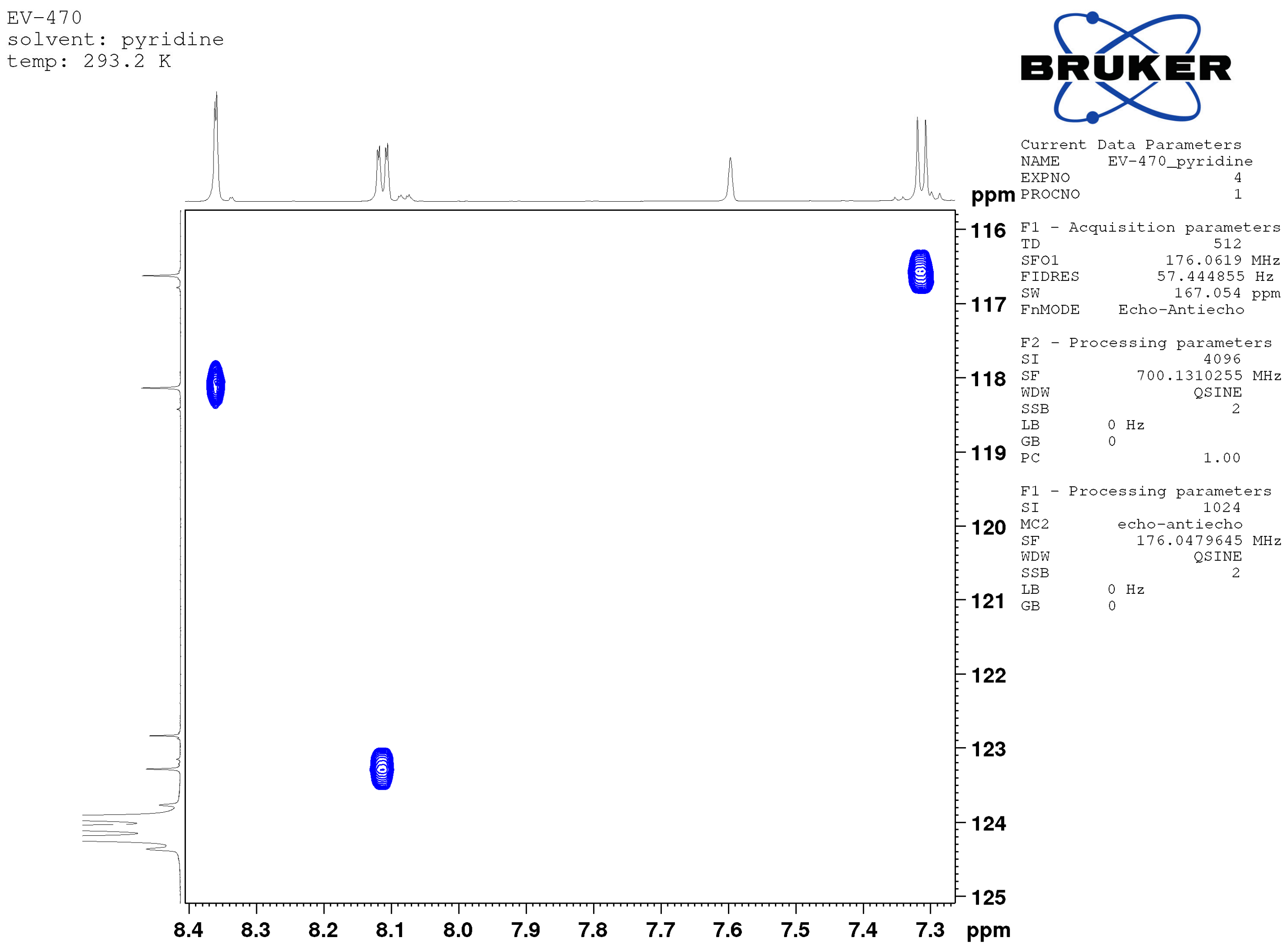

Figure S51. Detail (1/4) of HSQC NMR spectrum of compound **10**.

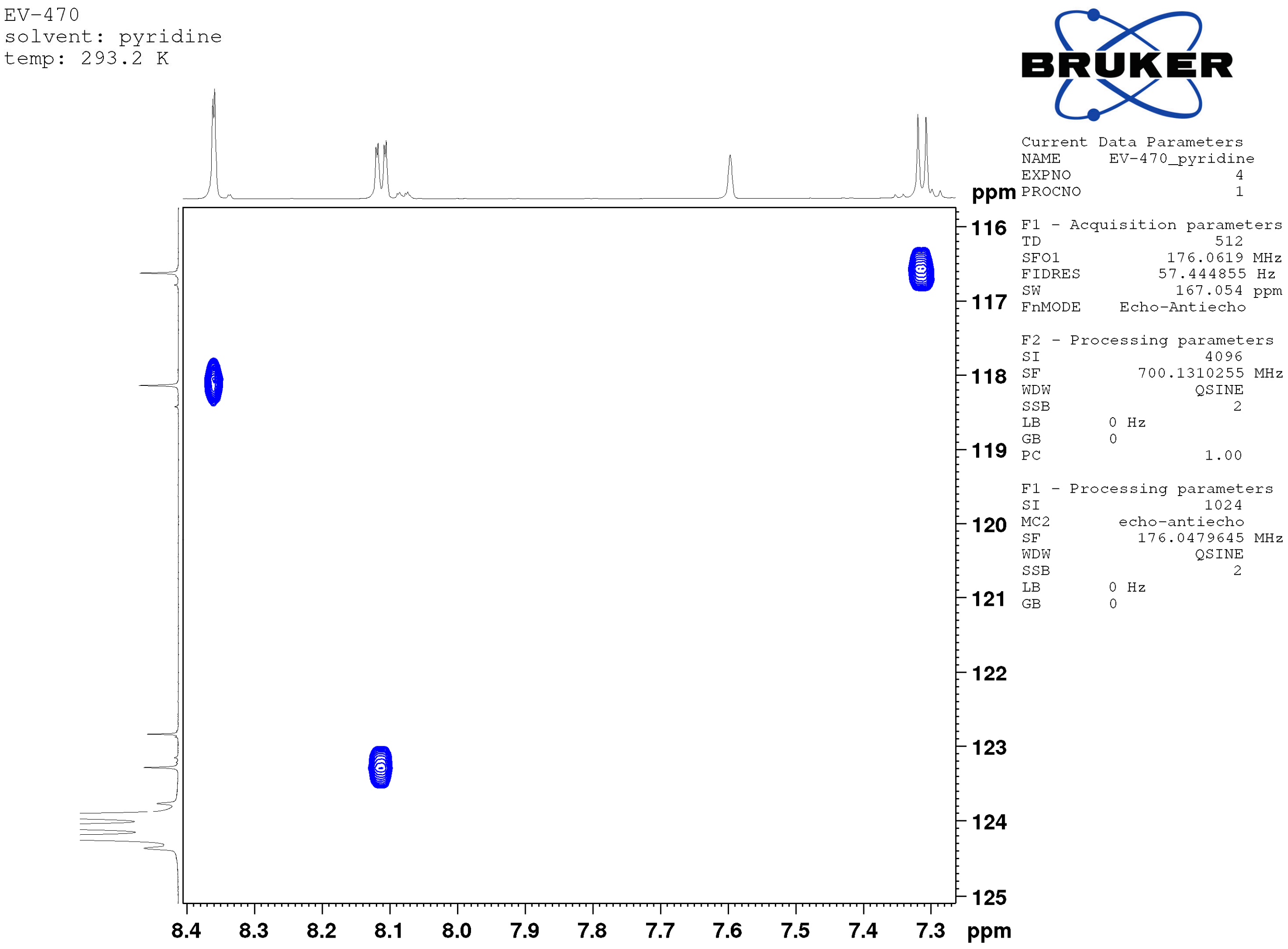

Figure S52. Detail (2/4) of HSQC NMR spectrum of compound **10**.

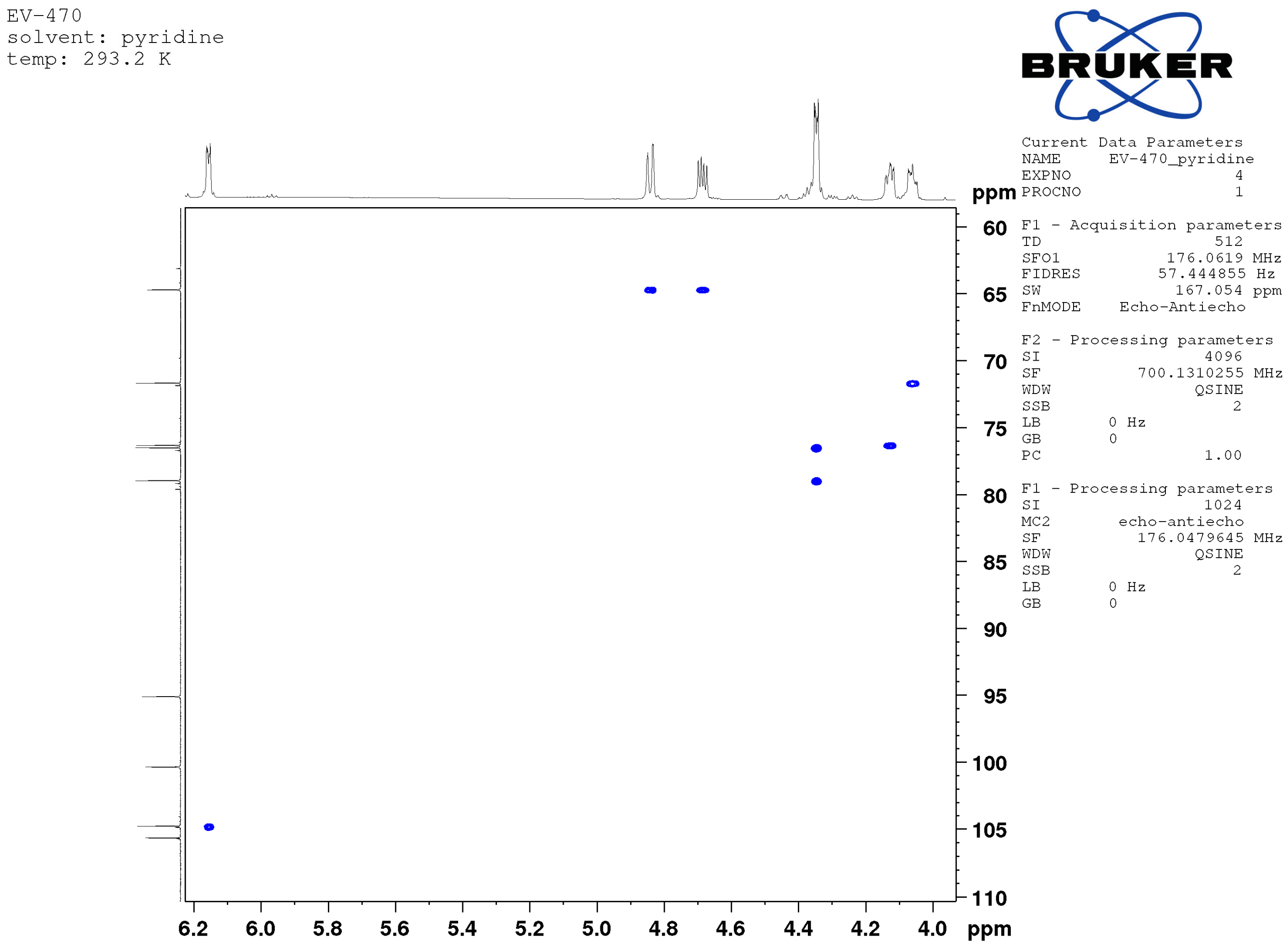

Figure S53. Detail (3/4) of HSQC NMR spectrum of compound **10**.

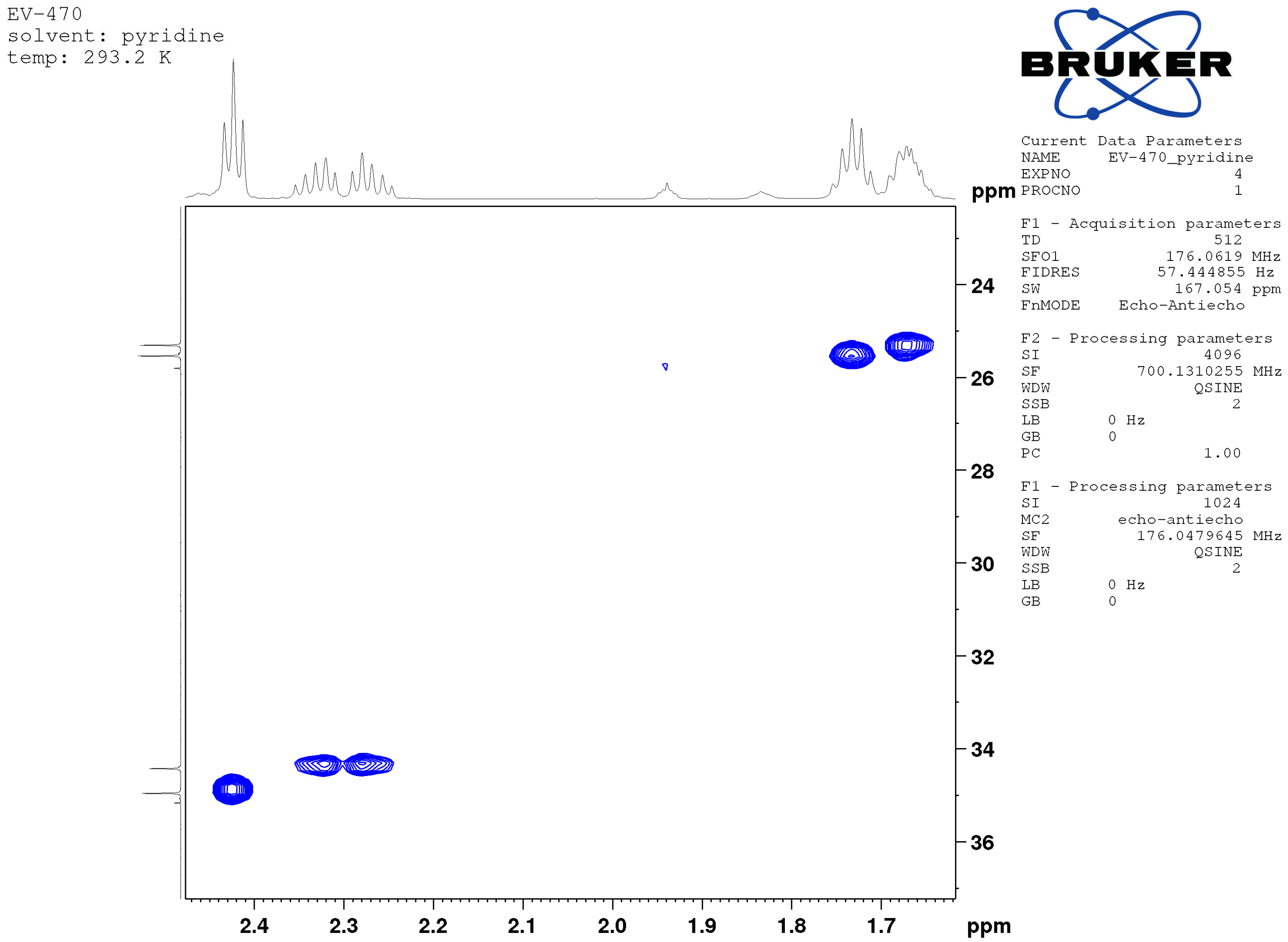

Figure S54. Detail (4/4) of HSQC NMR spectrum of compound **10**.

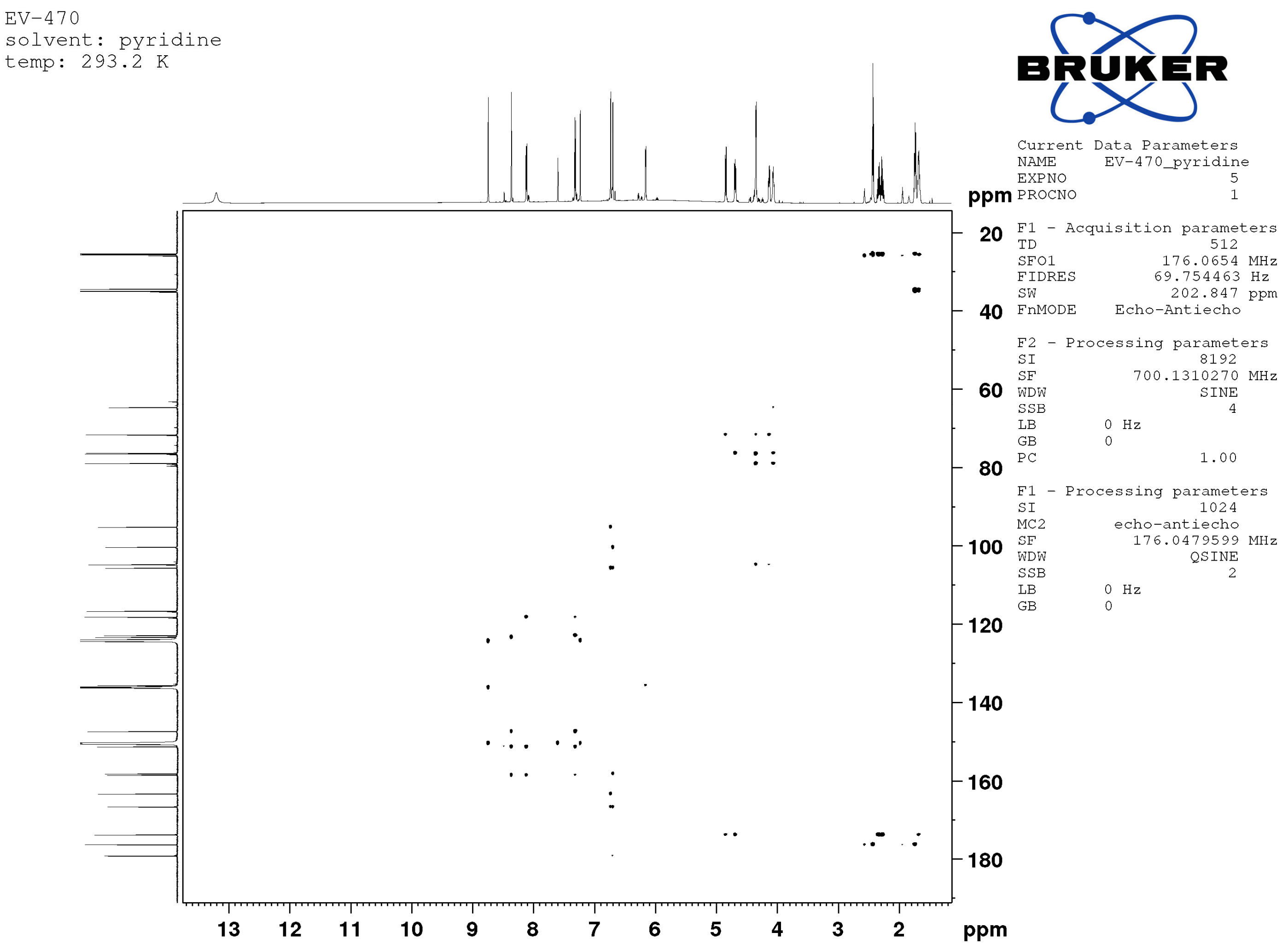

Figure S55. HMBC NMR spectrum of compound **10**.

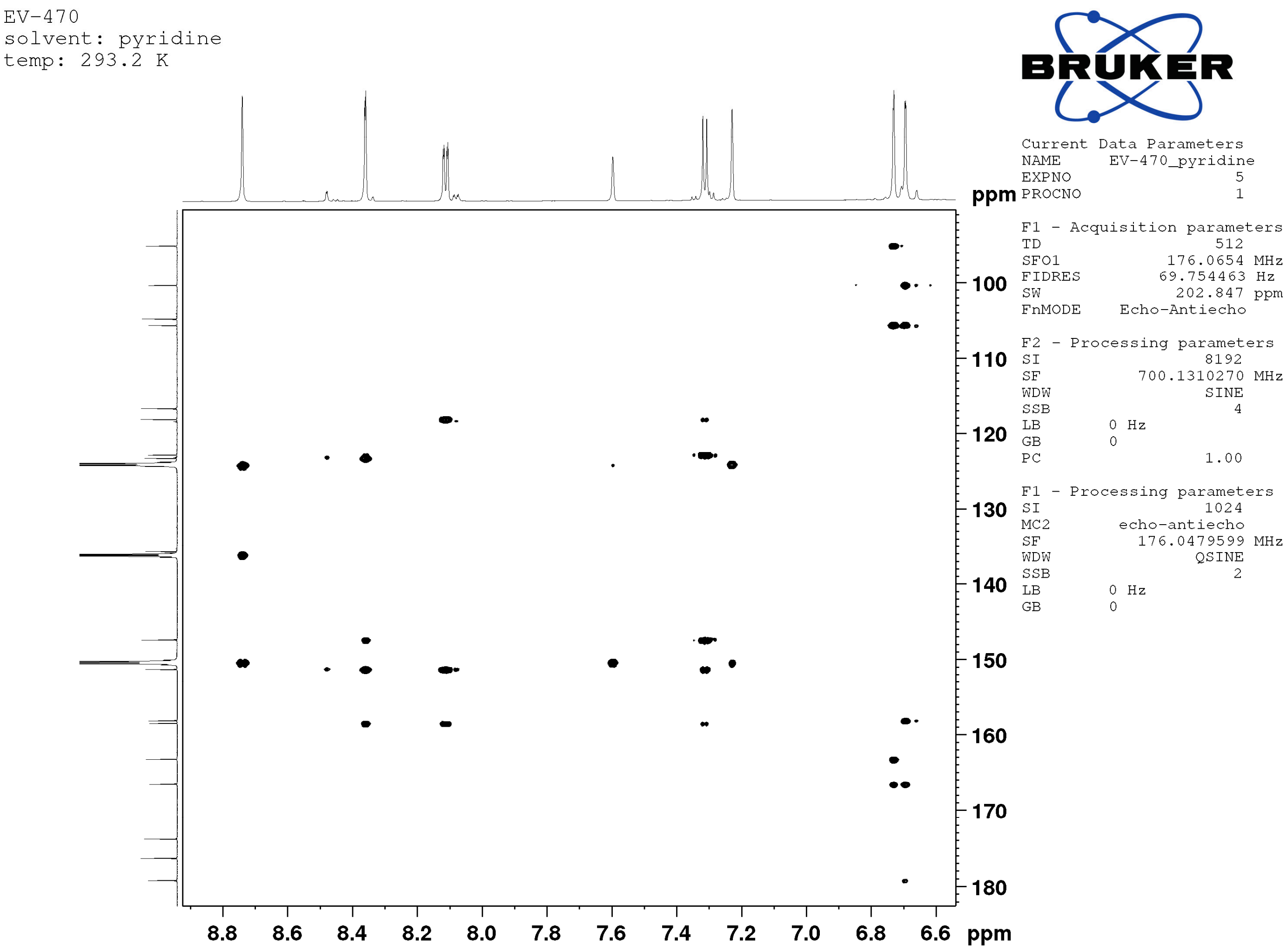

Figure S56. Detail (1/7) of HMBC NMR spectrum of compound **10**.

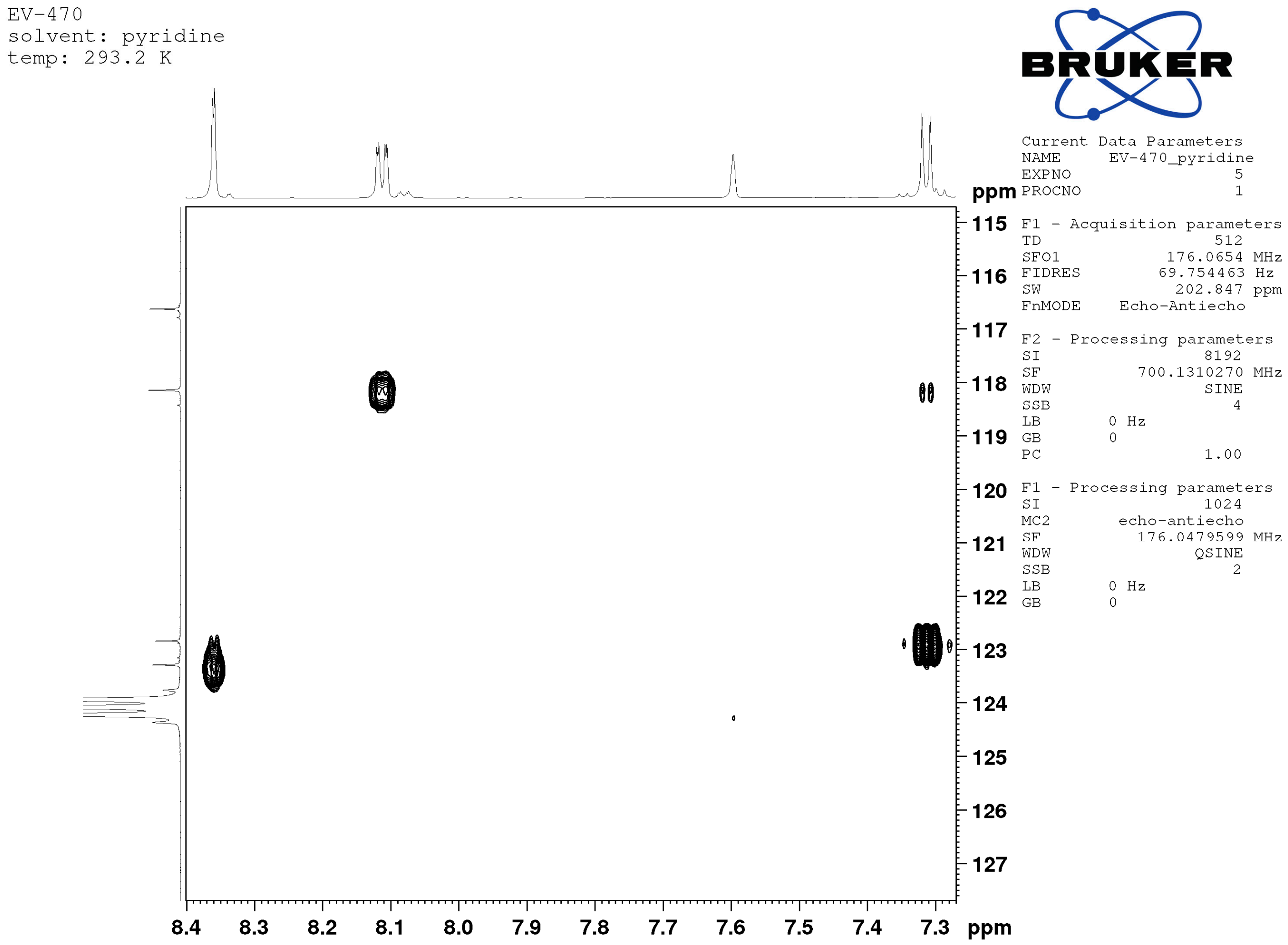

Figure S57. Detail (2/7) of HMBC NMR spectrum of compound **10**.

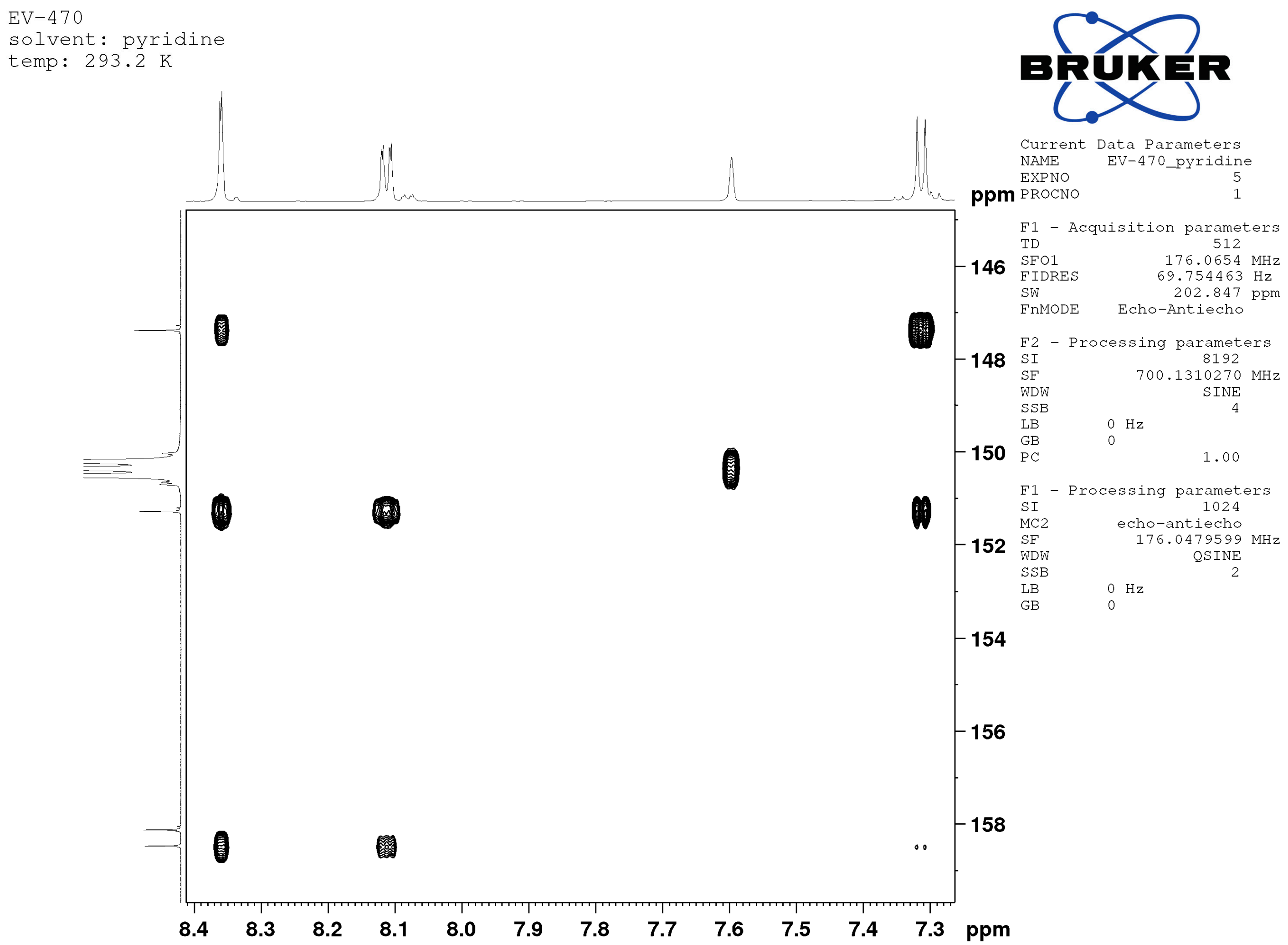

Figure S58. Detail (3/7) of HMBC NMR spectrum of compound **10**.

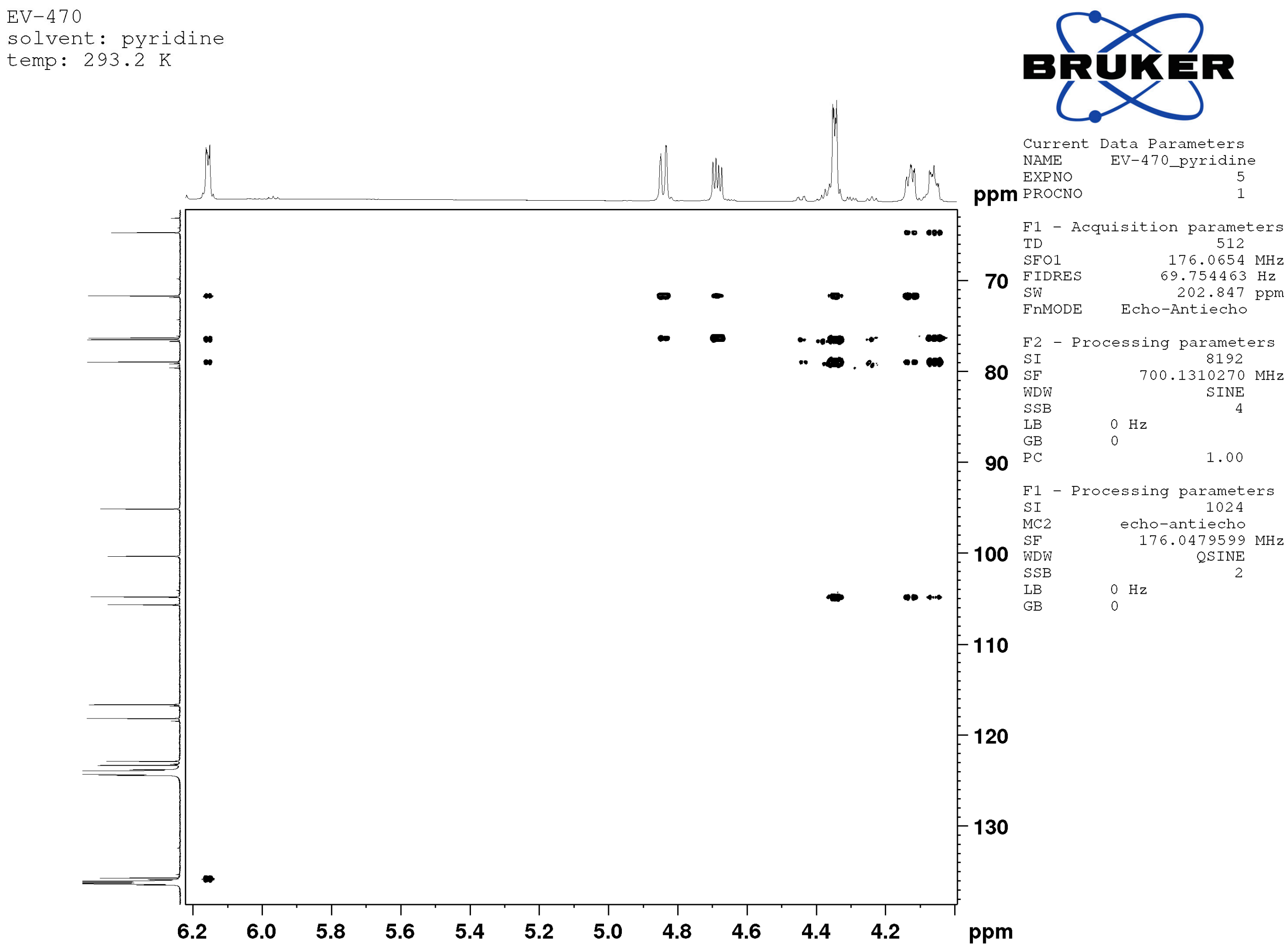

Figure S59. Detail (4/7) of HMBC NMR spectrum of compound **10**.

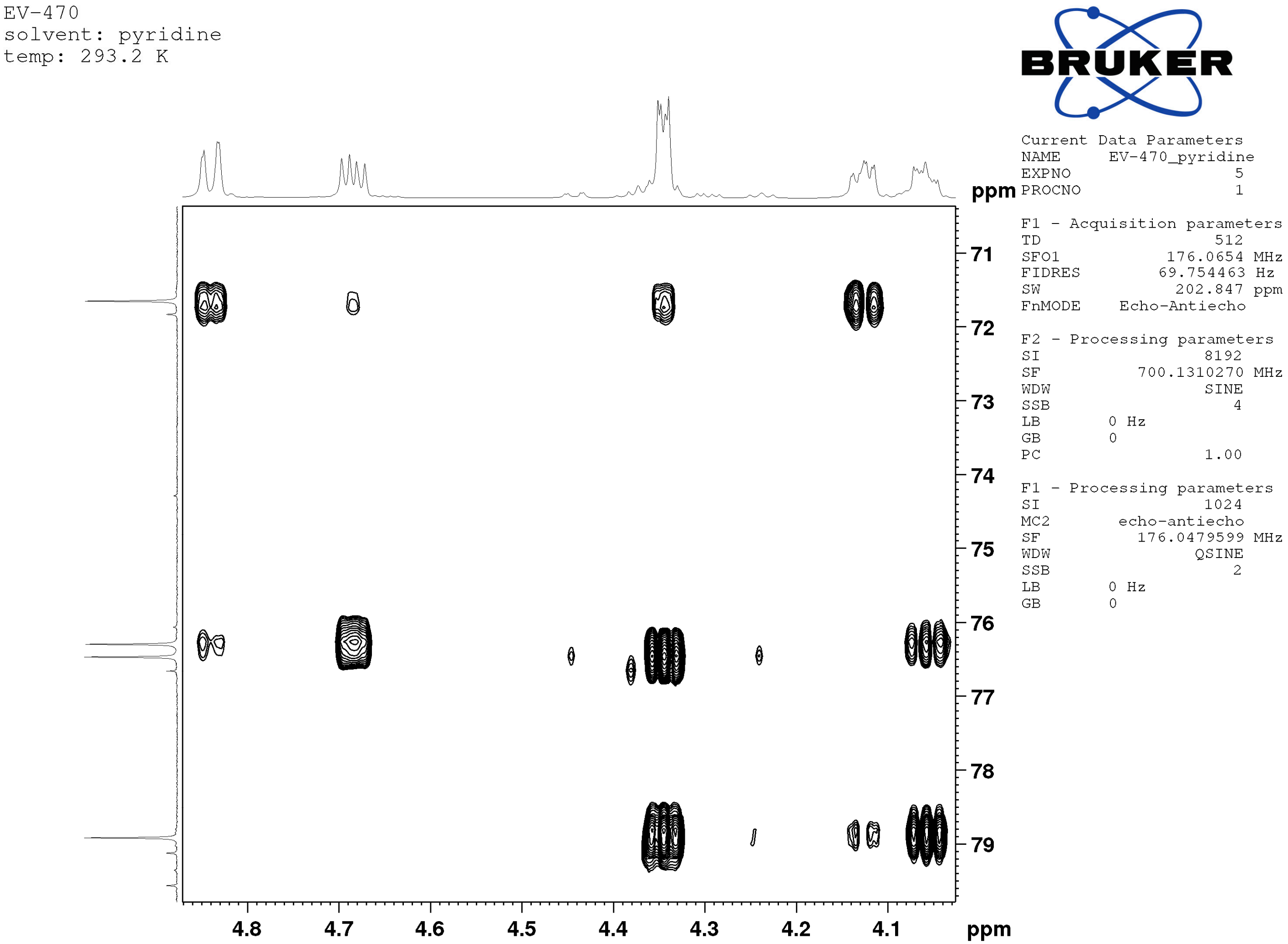

Figure S60. Detail (5/7) of HMBC NMR spectrum of compound **10**.

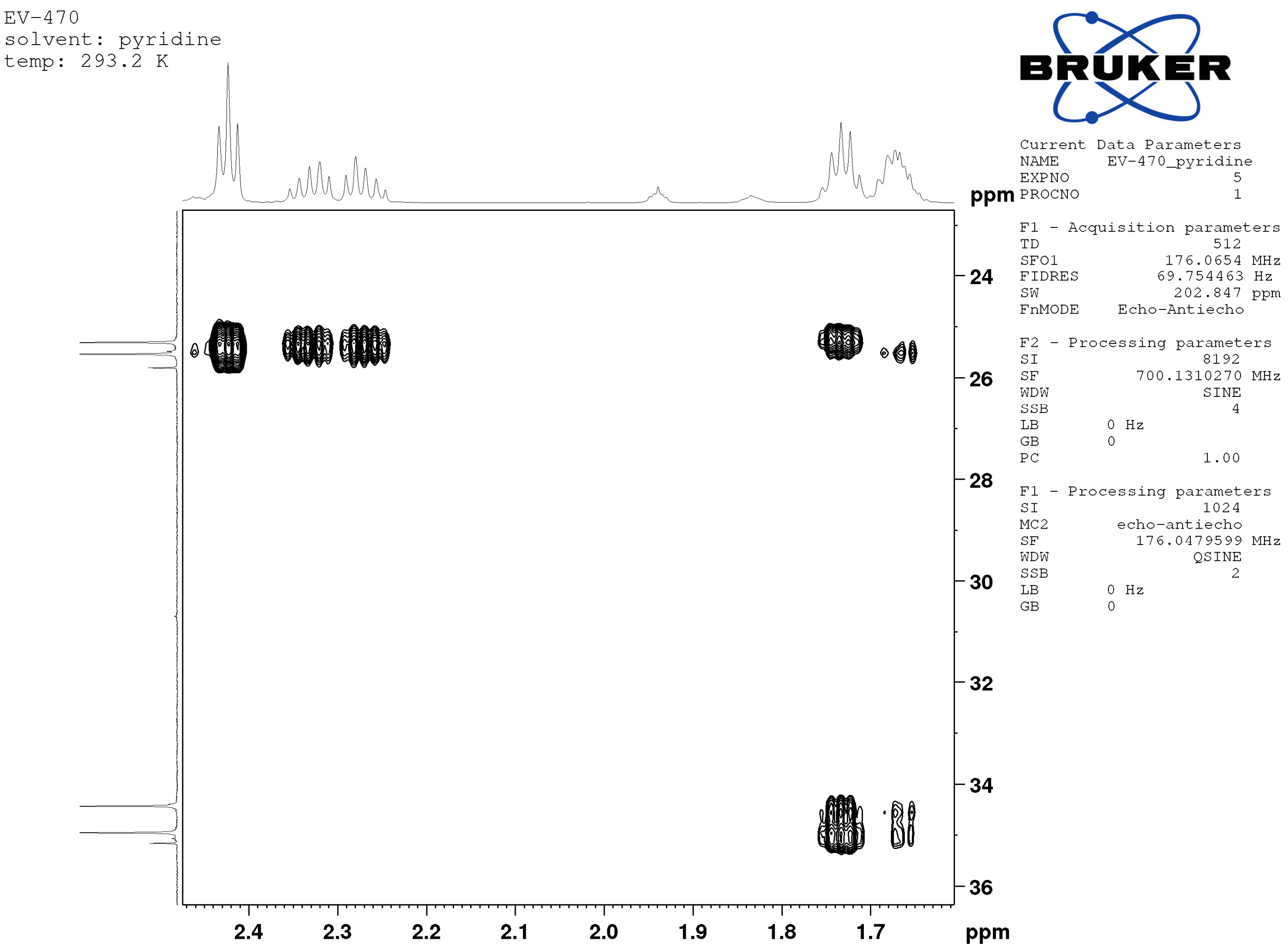

Figure S61. Detail (6/7) of HMBC NMR spectrum of compound **10**.

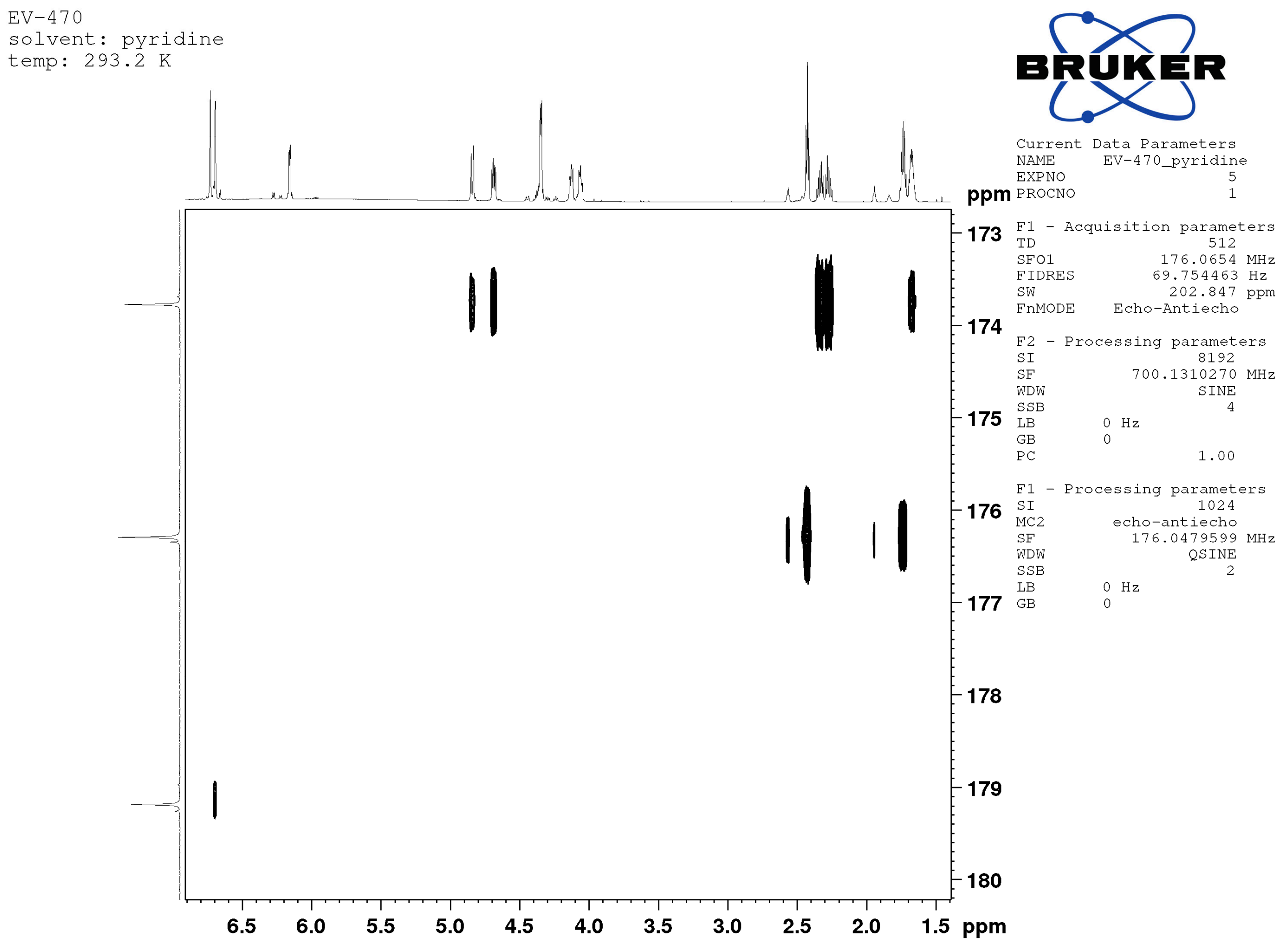

Figure S62. Detail (7/7) of HMBC NMR spectrum of compound **10**.

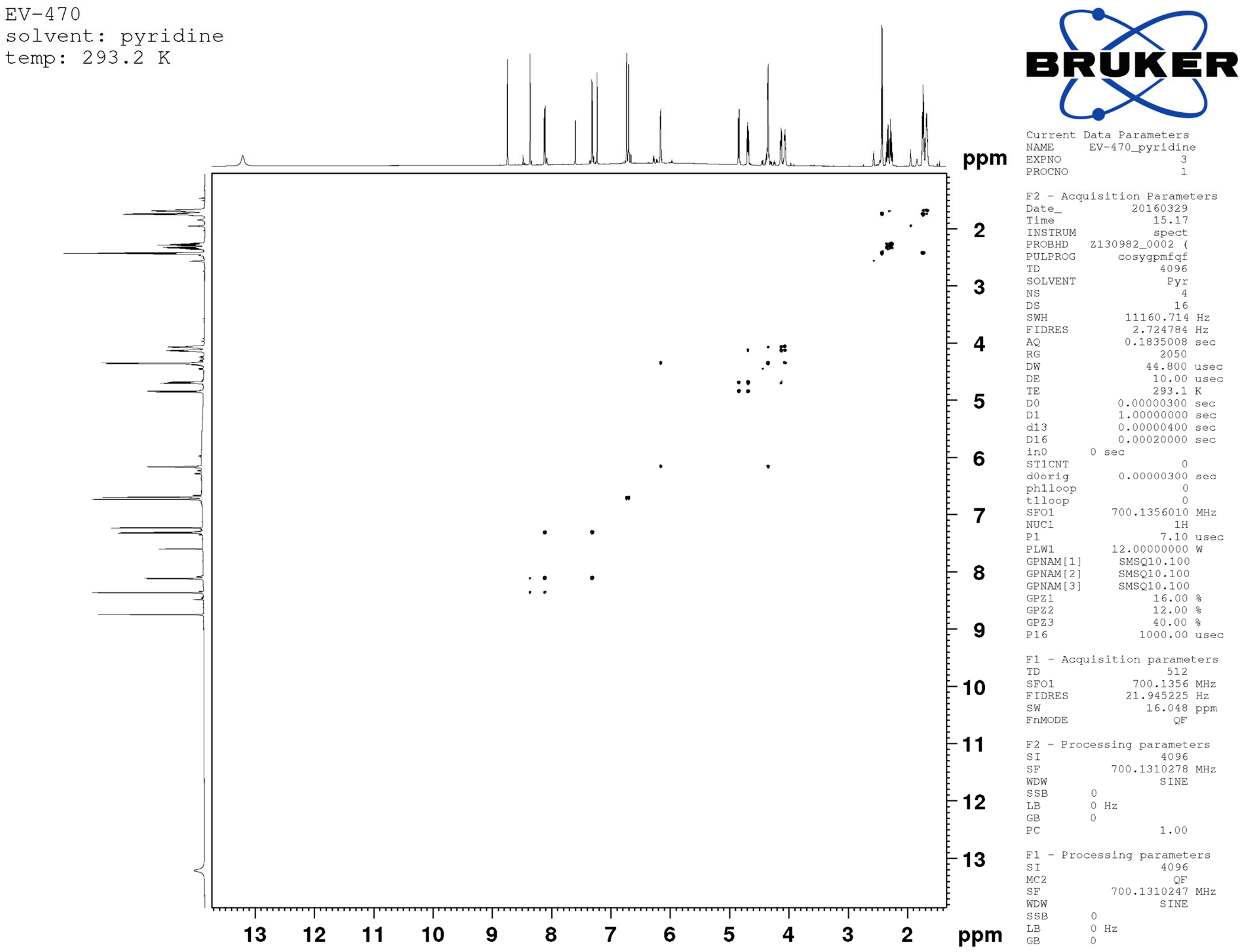

Figure S63. COSY NMR spectrum of compound **10**.

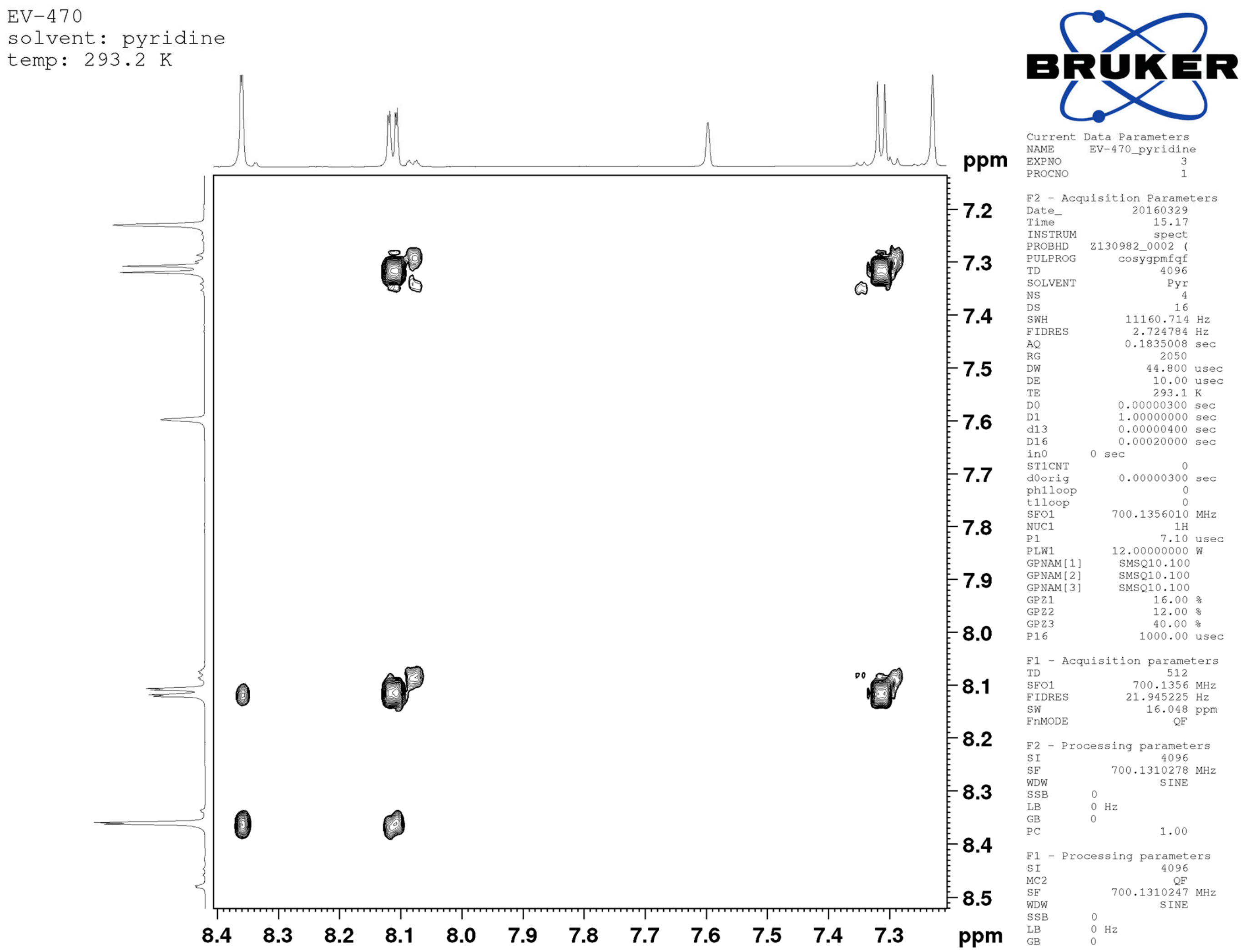

Figure S64. Detail (1/3) of COSY NMR spectrum of compound **10**.

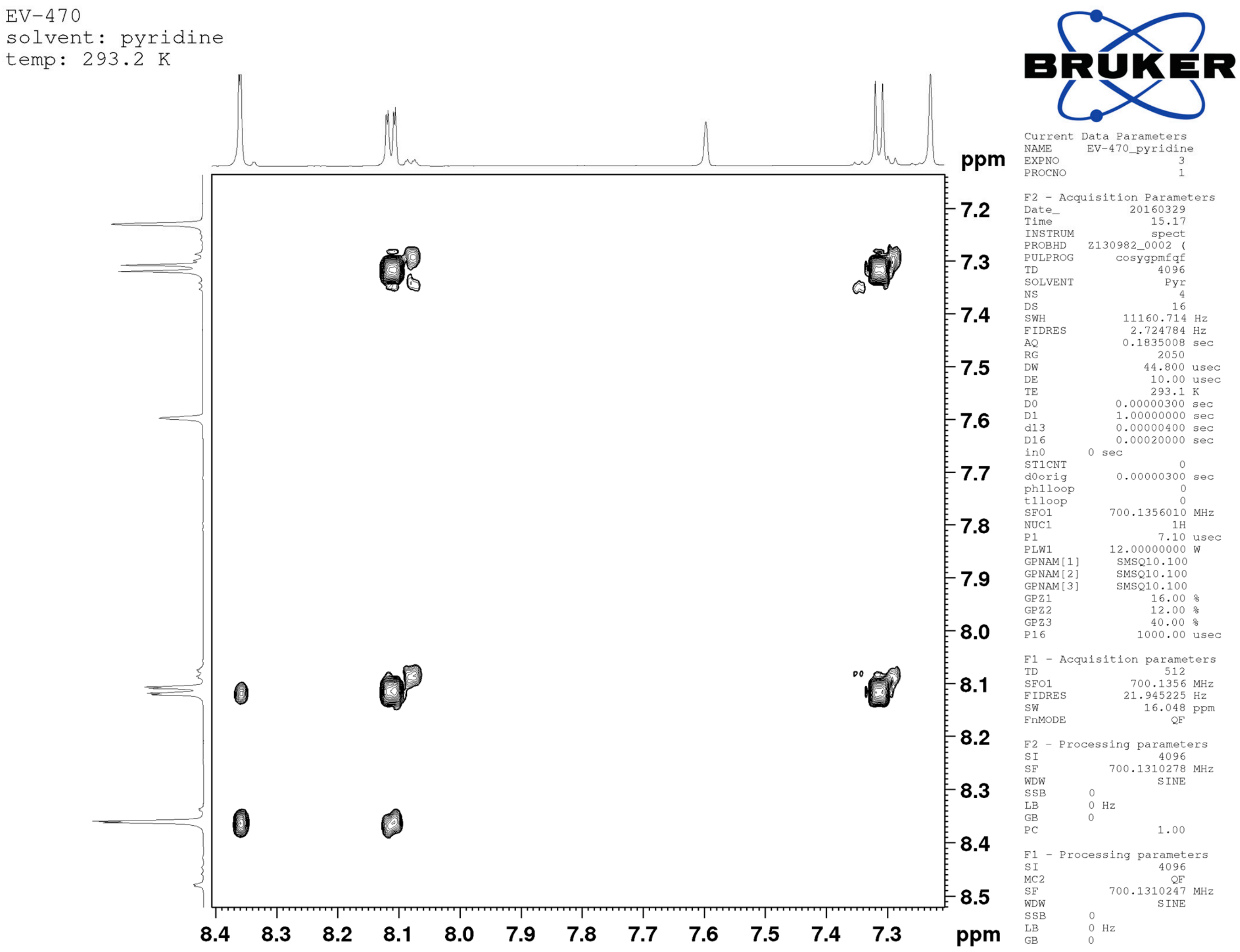

Figure S65. Detail (2/3) of COSY NMR spectrum of compound **10**.

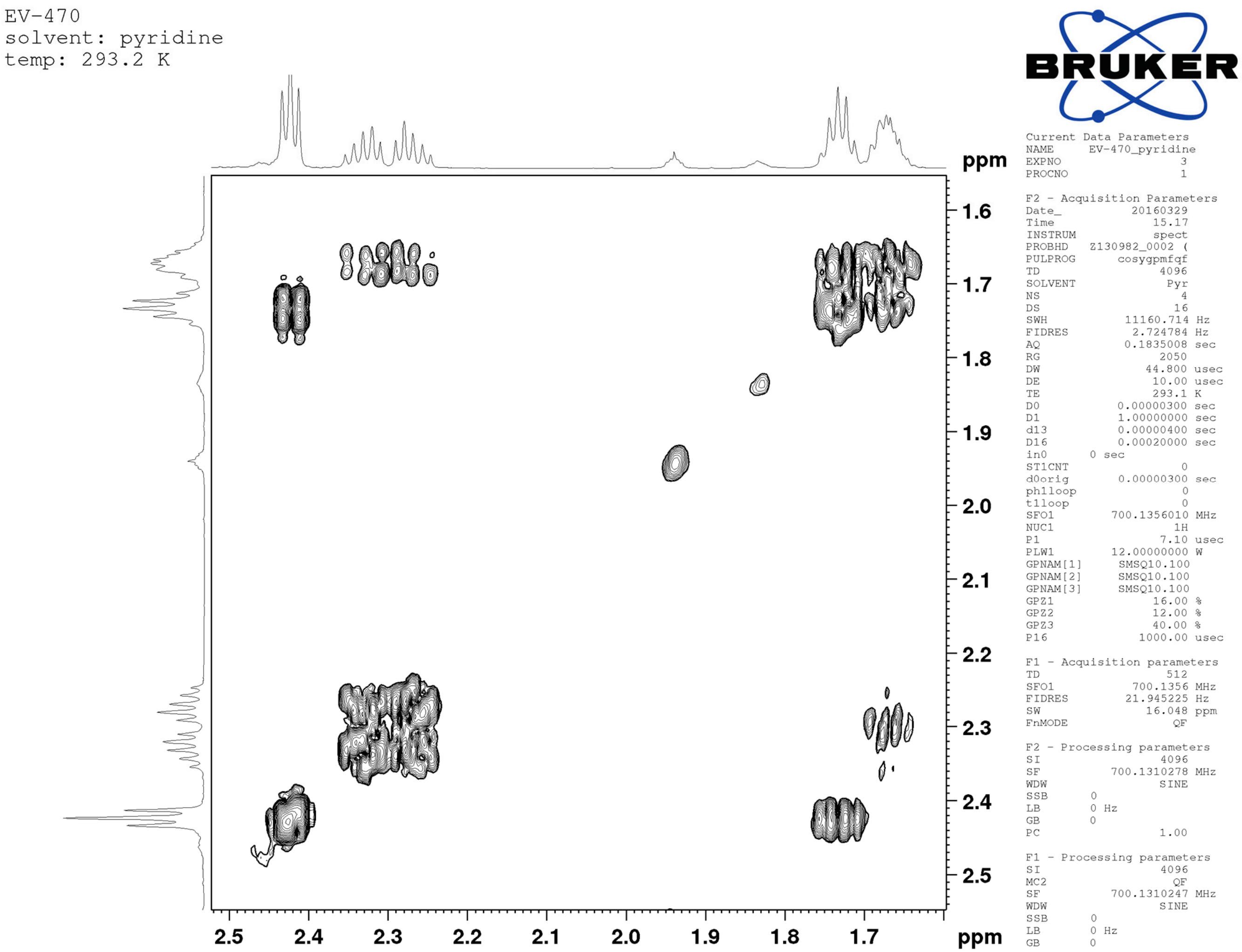

Figure S66. Detail (3/3) of COSY NMR spectrum of compound **10**.

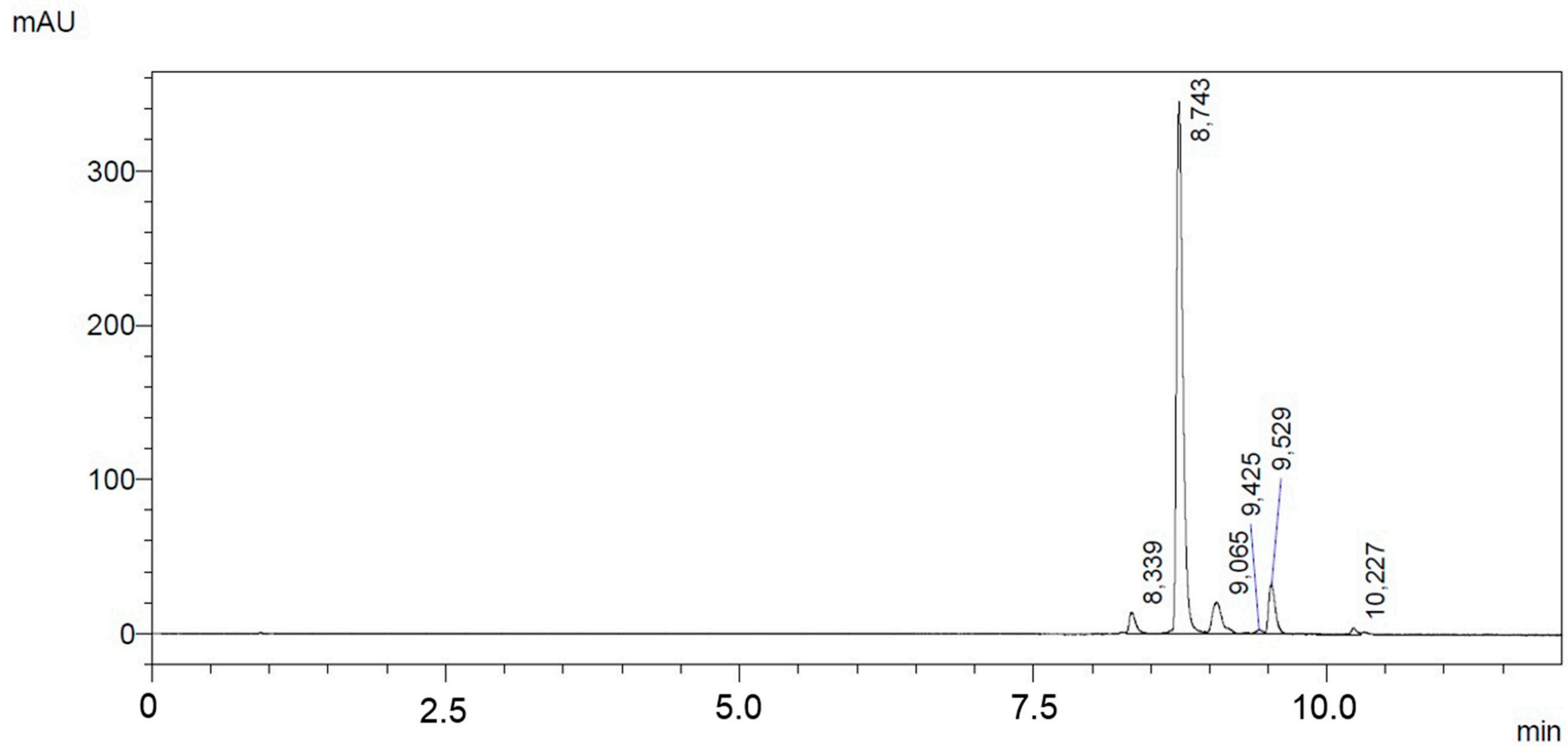

Figure S67. HPLC chromatogram of compound **10**.

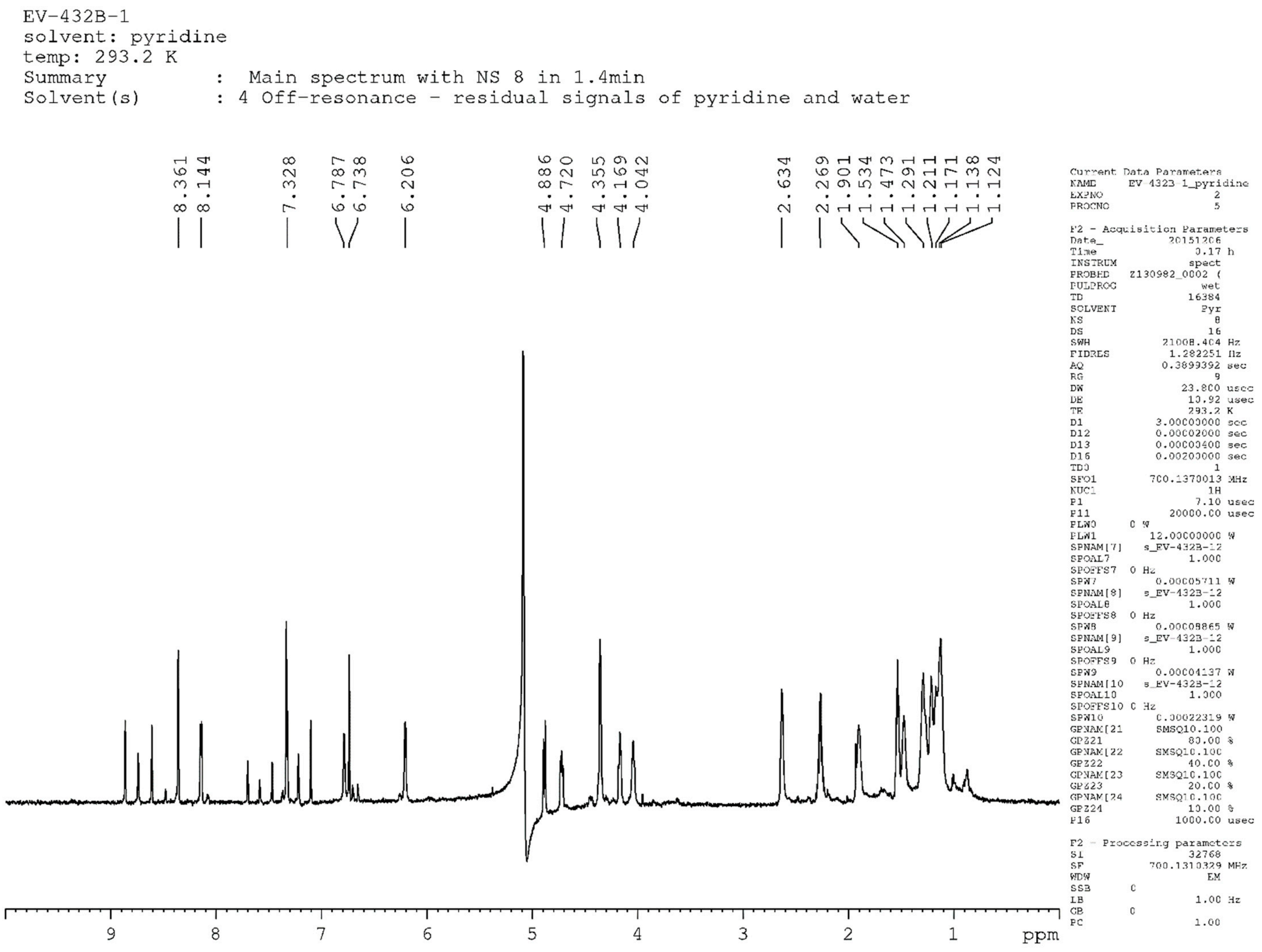

Figure S68. ^{1}H NMR spectrum of compound **11**.

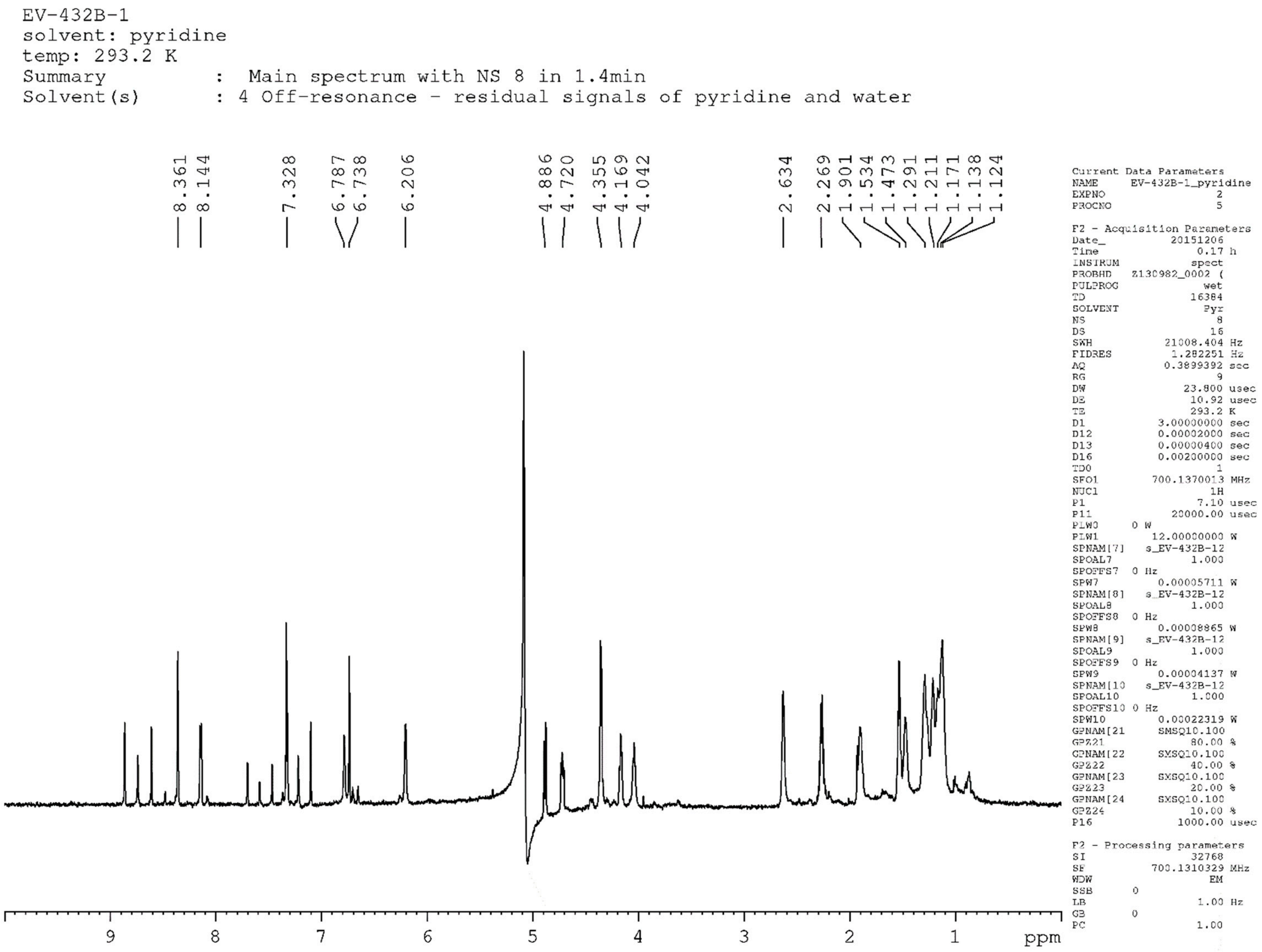

Figure S69. ^{1}H NMR spectrum WET of compound **11**.

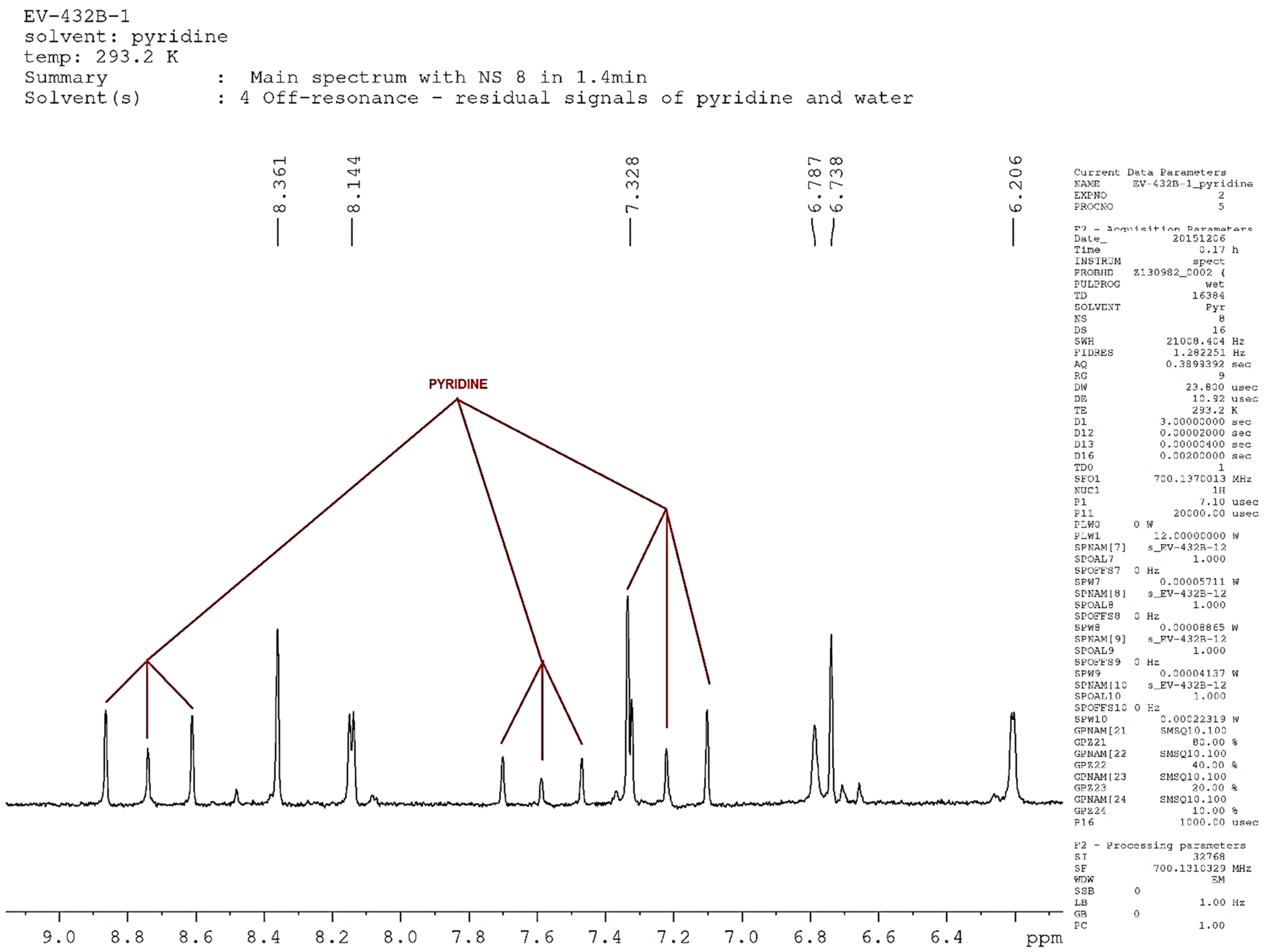

Figure S70. Detail (1/3) of ^{1}H NMR spectrum WET of compound **11**.

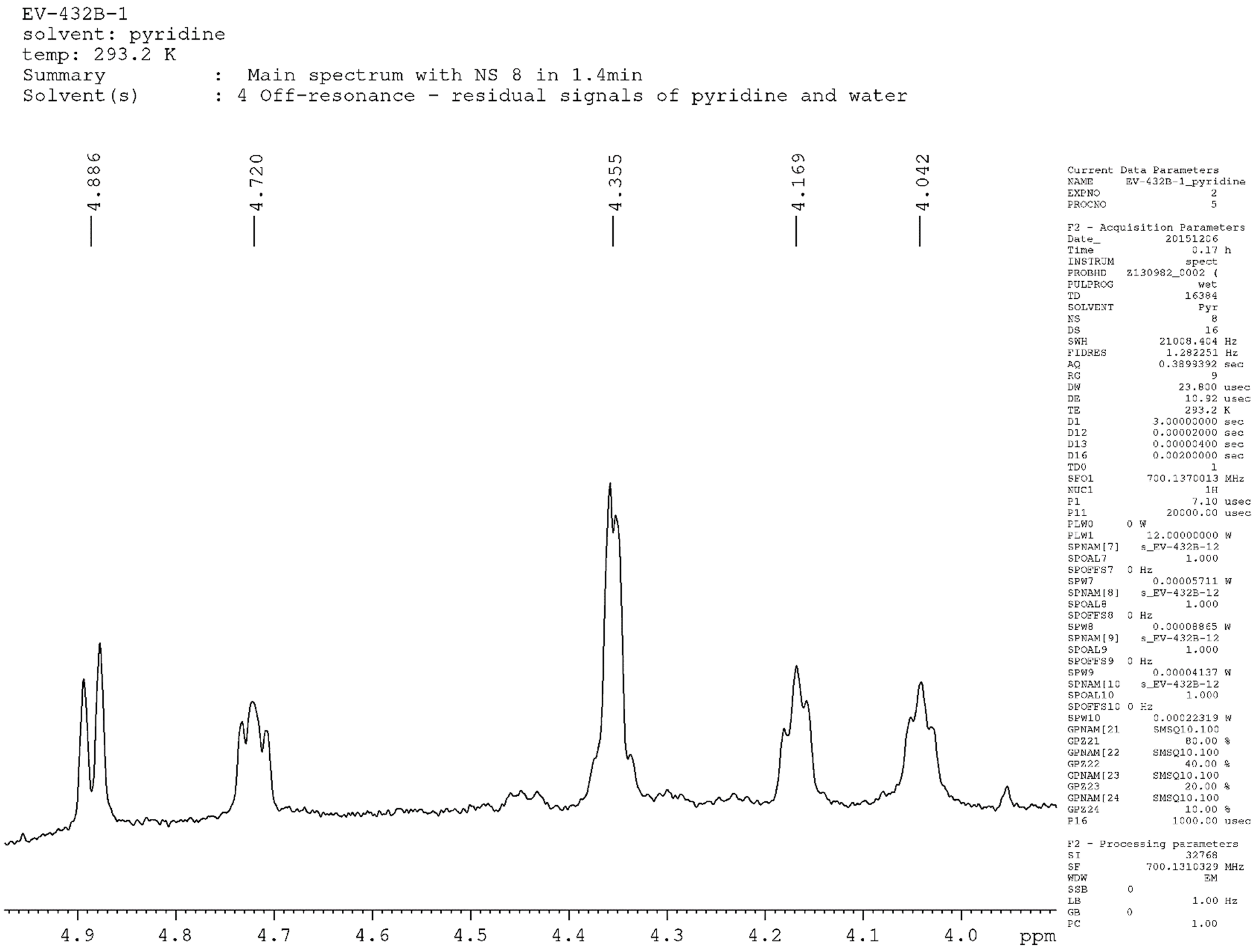

Figure S71. Detail (2/3) of ^{1}H NMR spectrum WET of compound **11**.

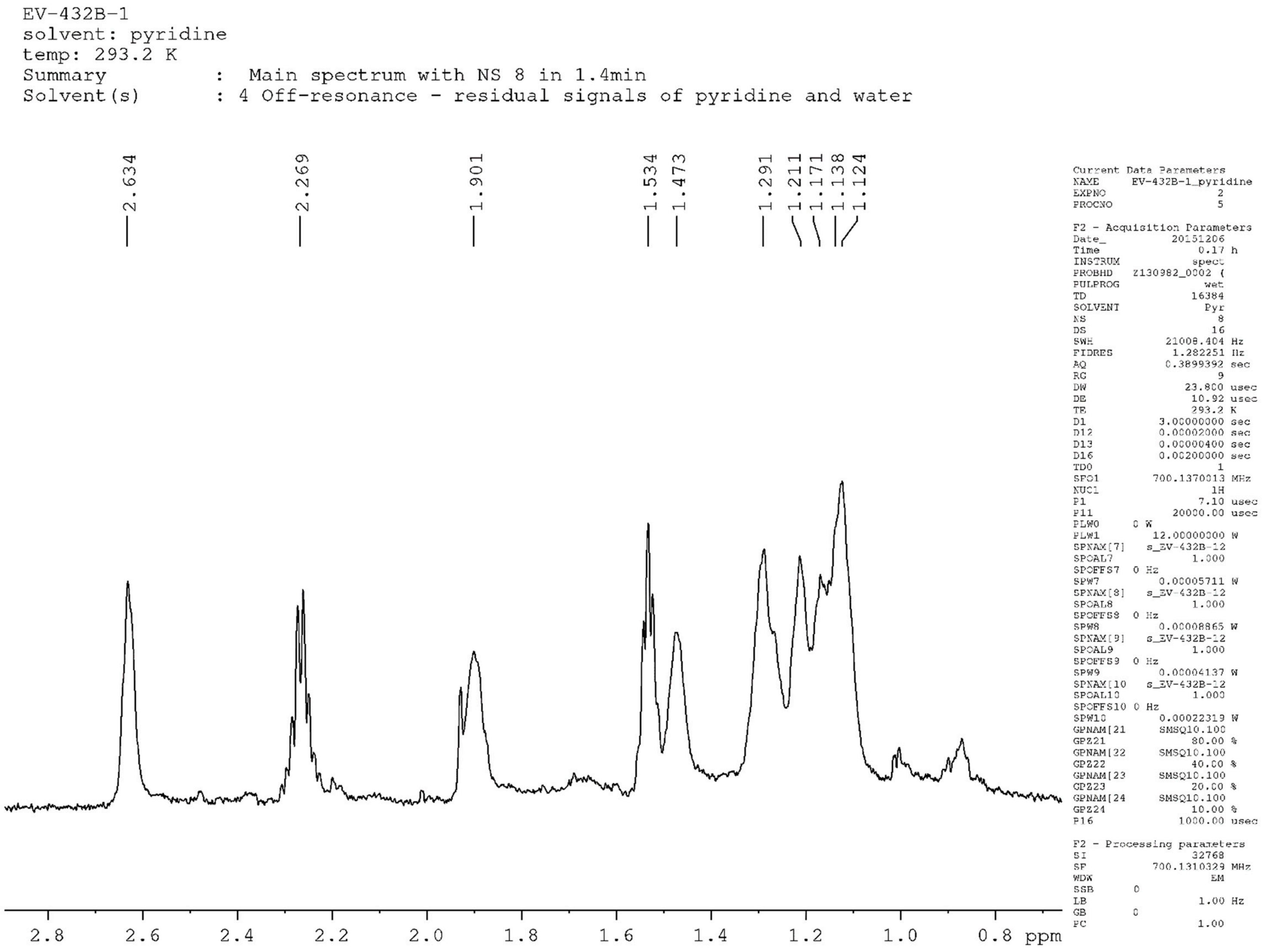

Figure S72. Detail (3/3) of ^{1}H NMR spectrum WET of compound **11**.

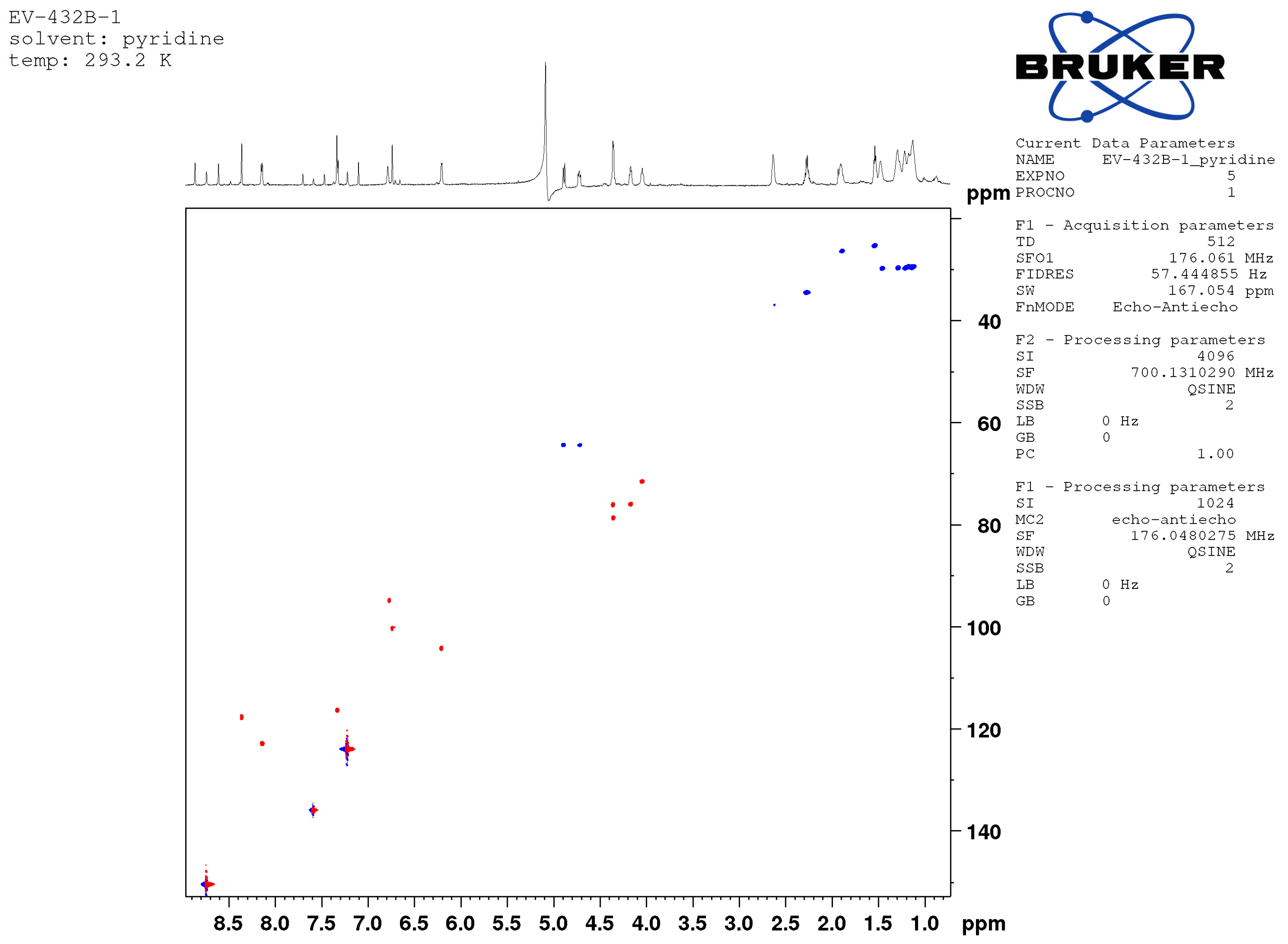

Figure S73. HSQC NMR spectrum of compound **11**.

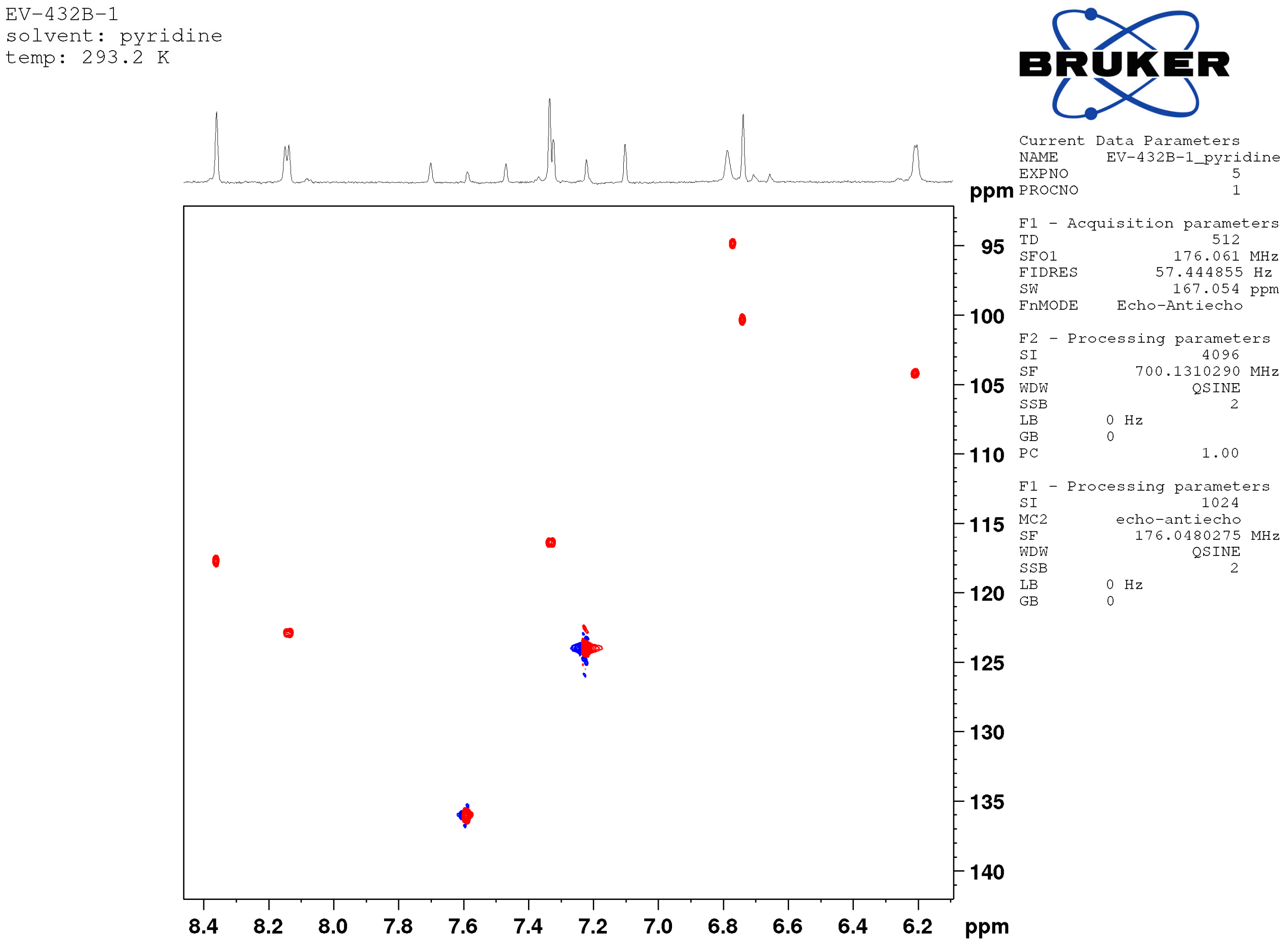

Figure S74. Detail (1/3) of HSQC NMR spectrum of compound **11**.

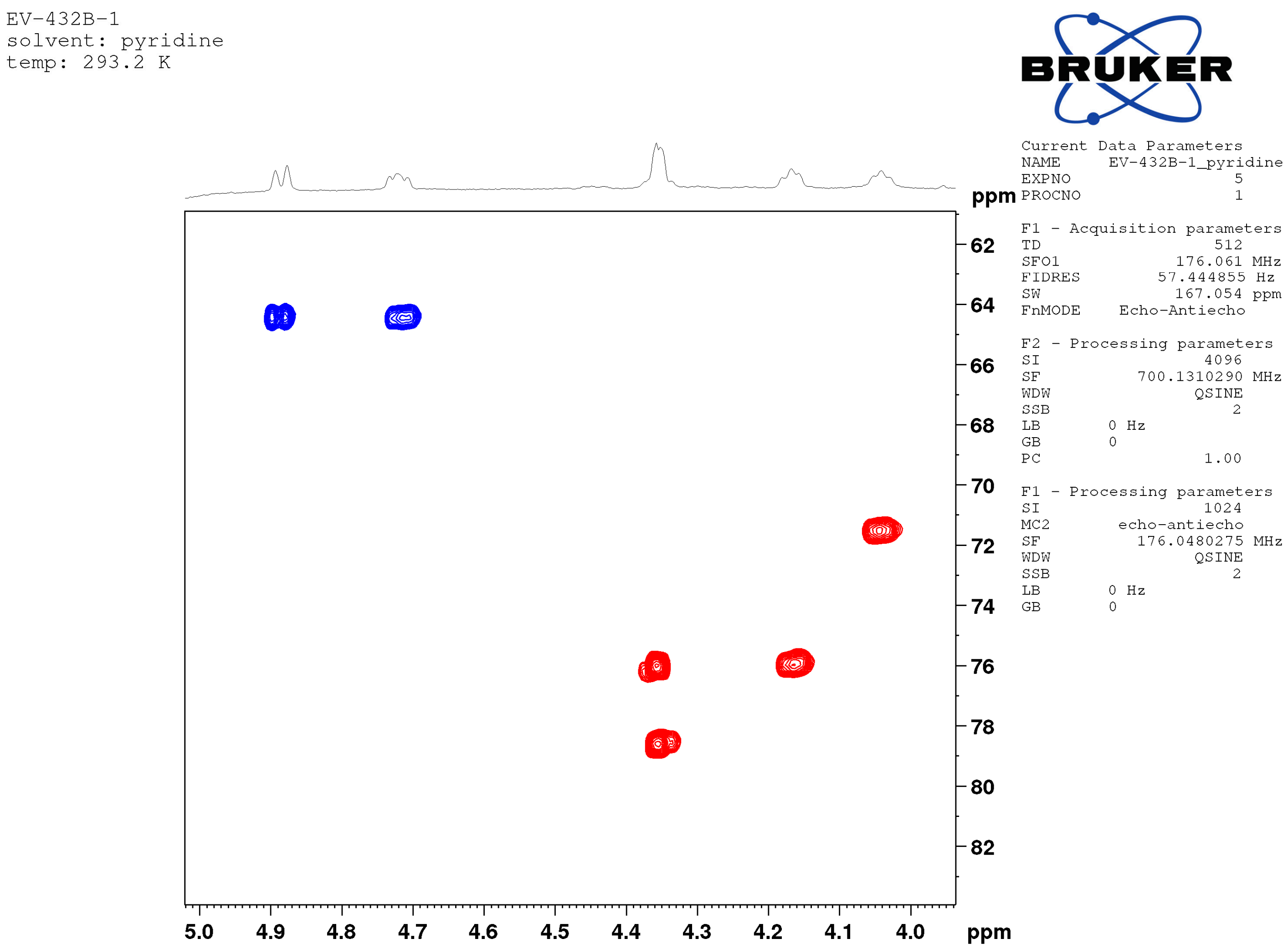

Figure S75. Detail (2/3) of HSQC NMR spectrum of compound **11**.

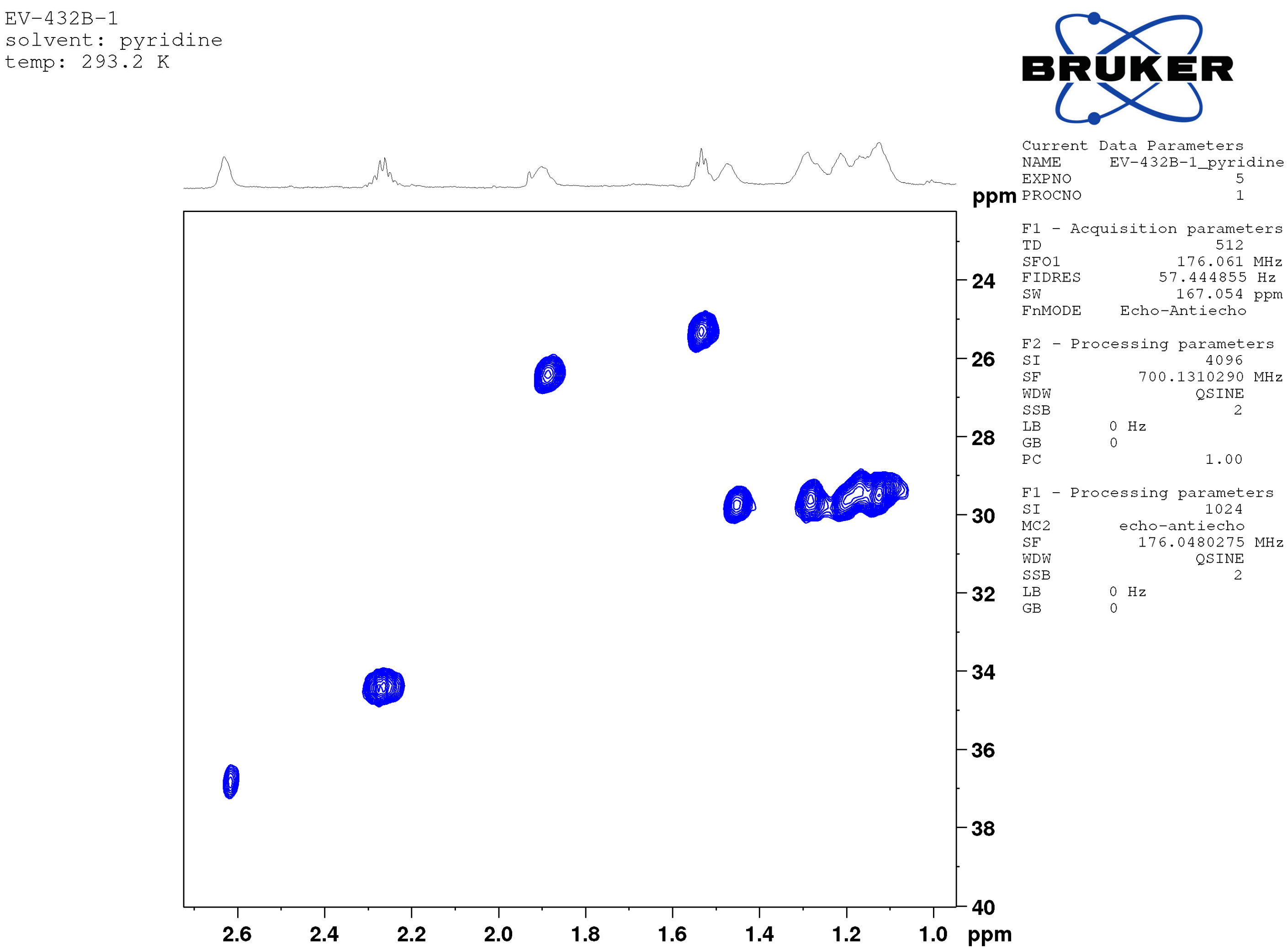

Figure S76. Detail (3/3) of HSQC NMR spectrum of compound **11**.

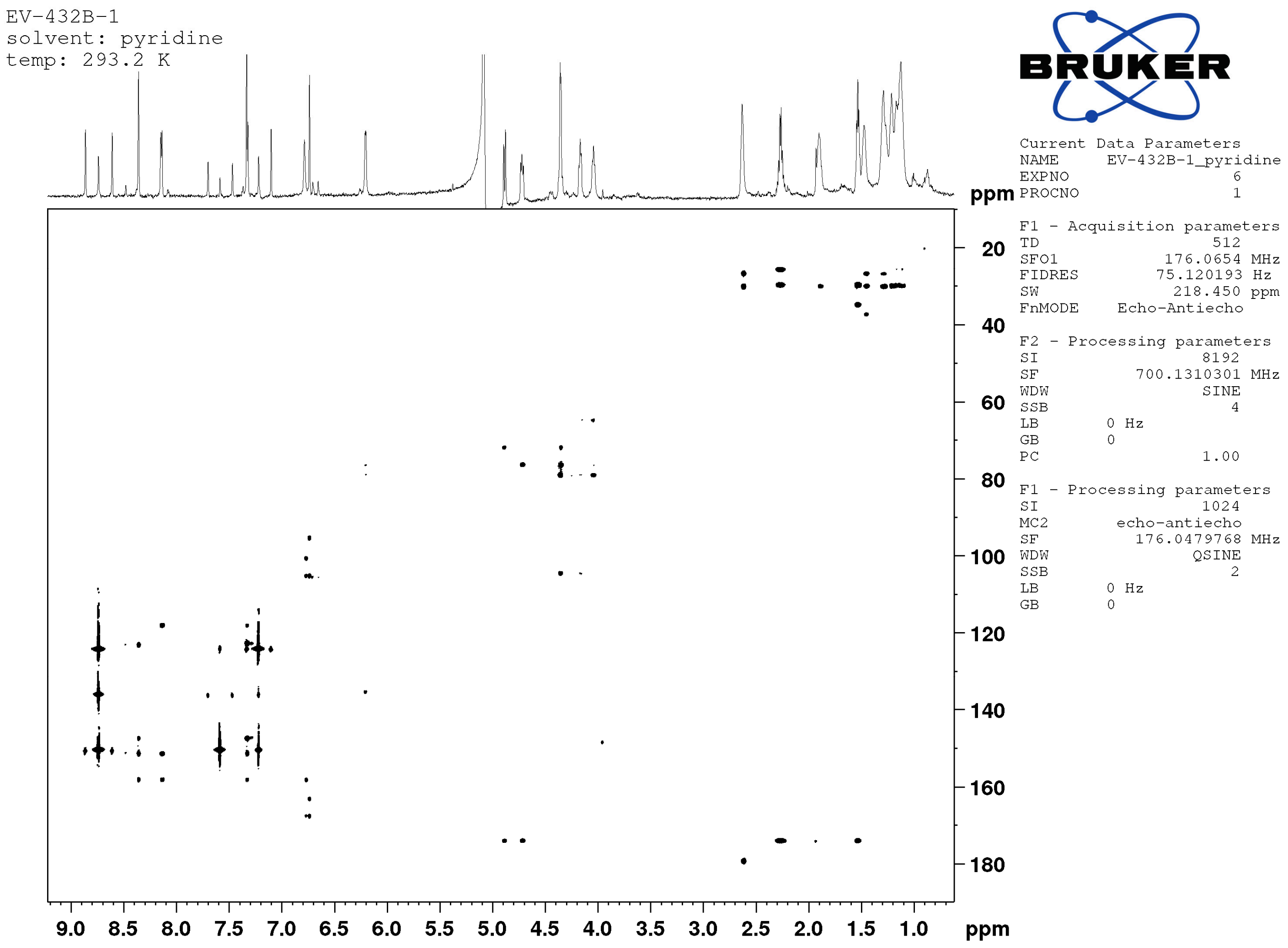

Figure S77. HMBC NMR spectrum of compound **11**.

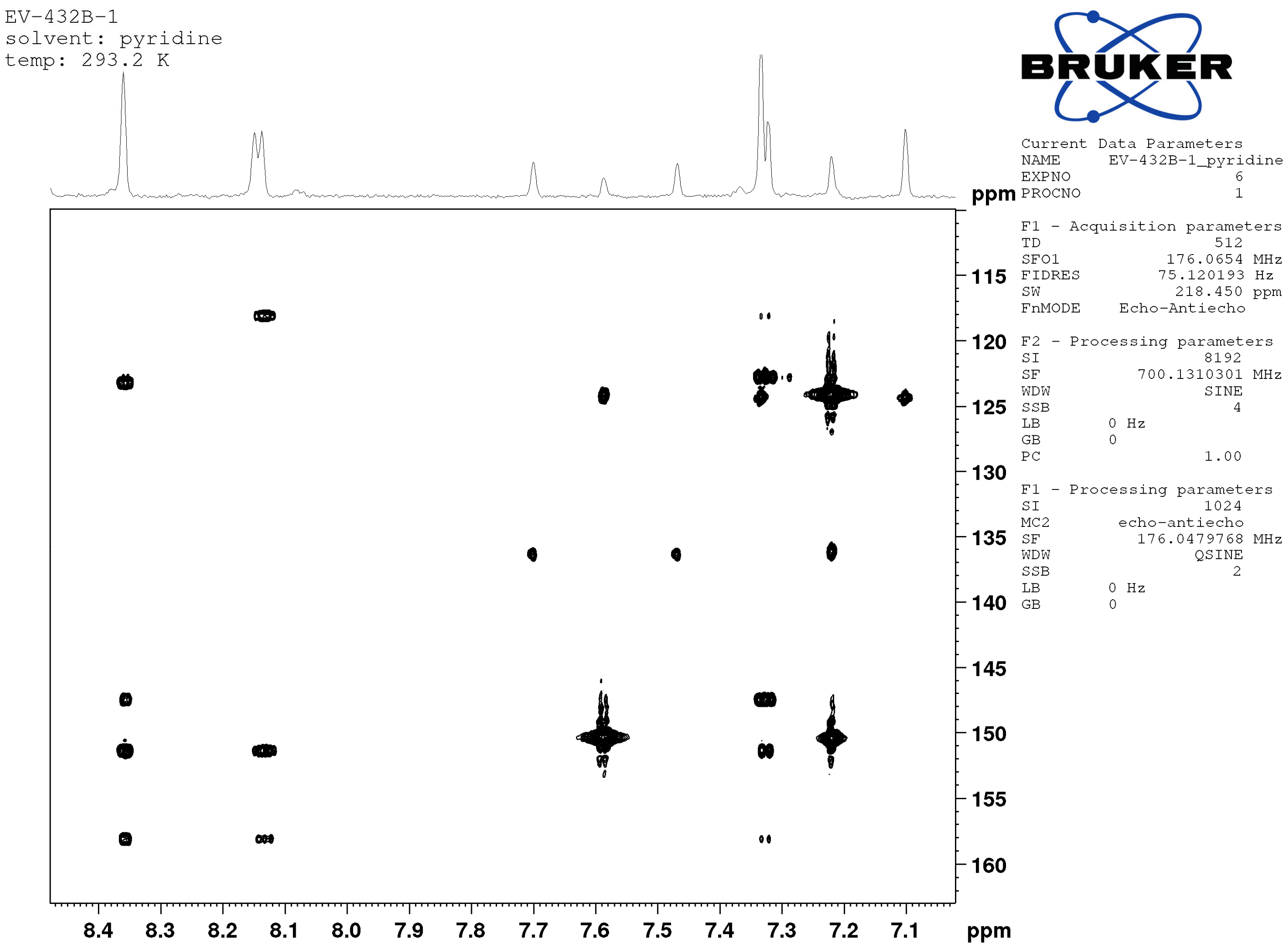

Figure S78. Detail (1/5) of HMBC NMR spectrum of compound **11**.

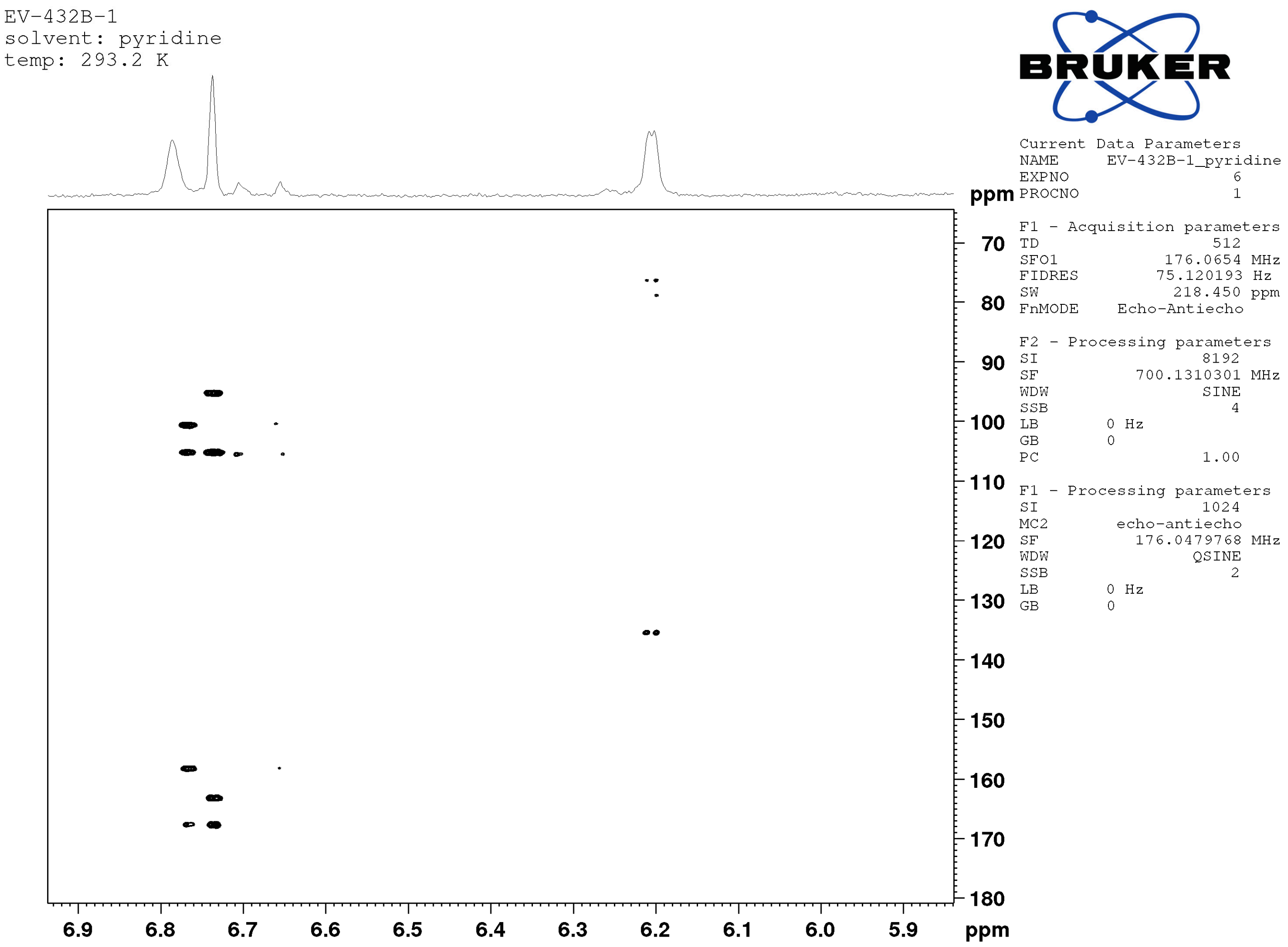

Figure S79. Detail (2/5) of HMBC NMR spectrum of compound **11**.

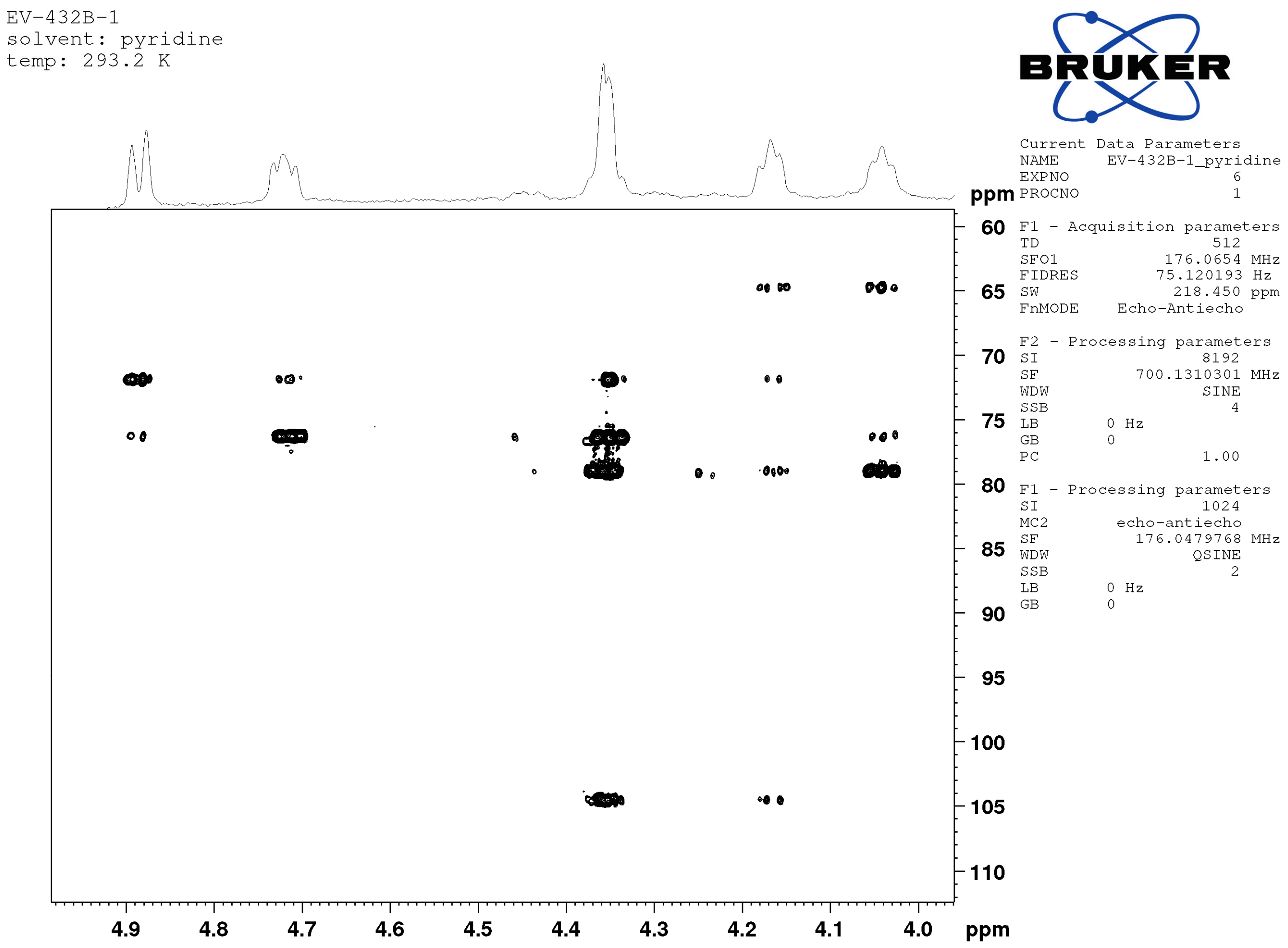

Figure S80. Detail (3/5) of HMBC NMR spectrum of compound **11**.

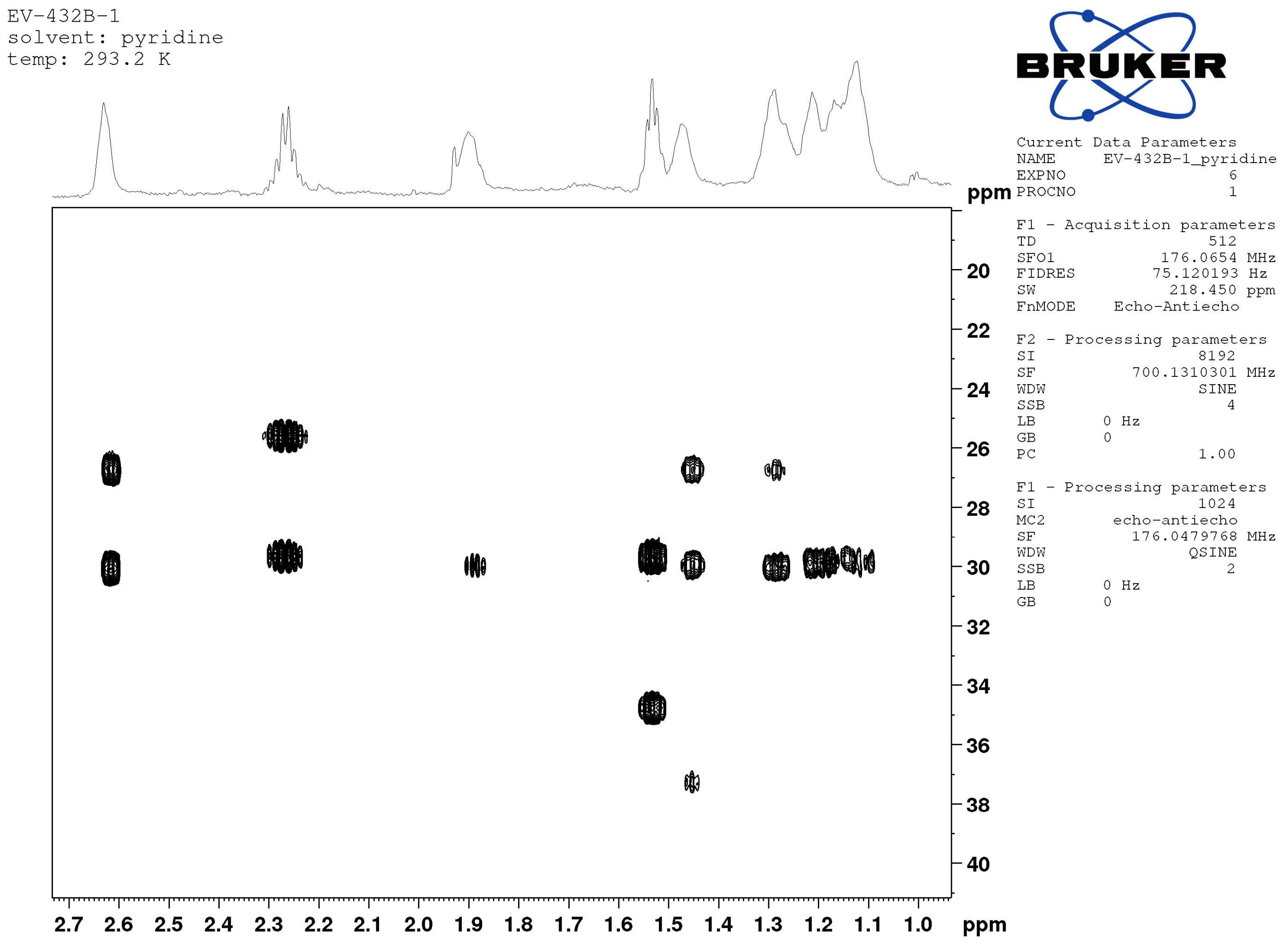

Figure S81. Detail (4/5) of HMBC NMR spectrum of compound **11**.

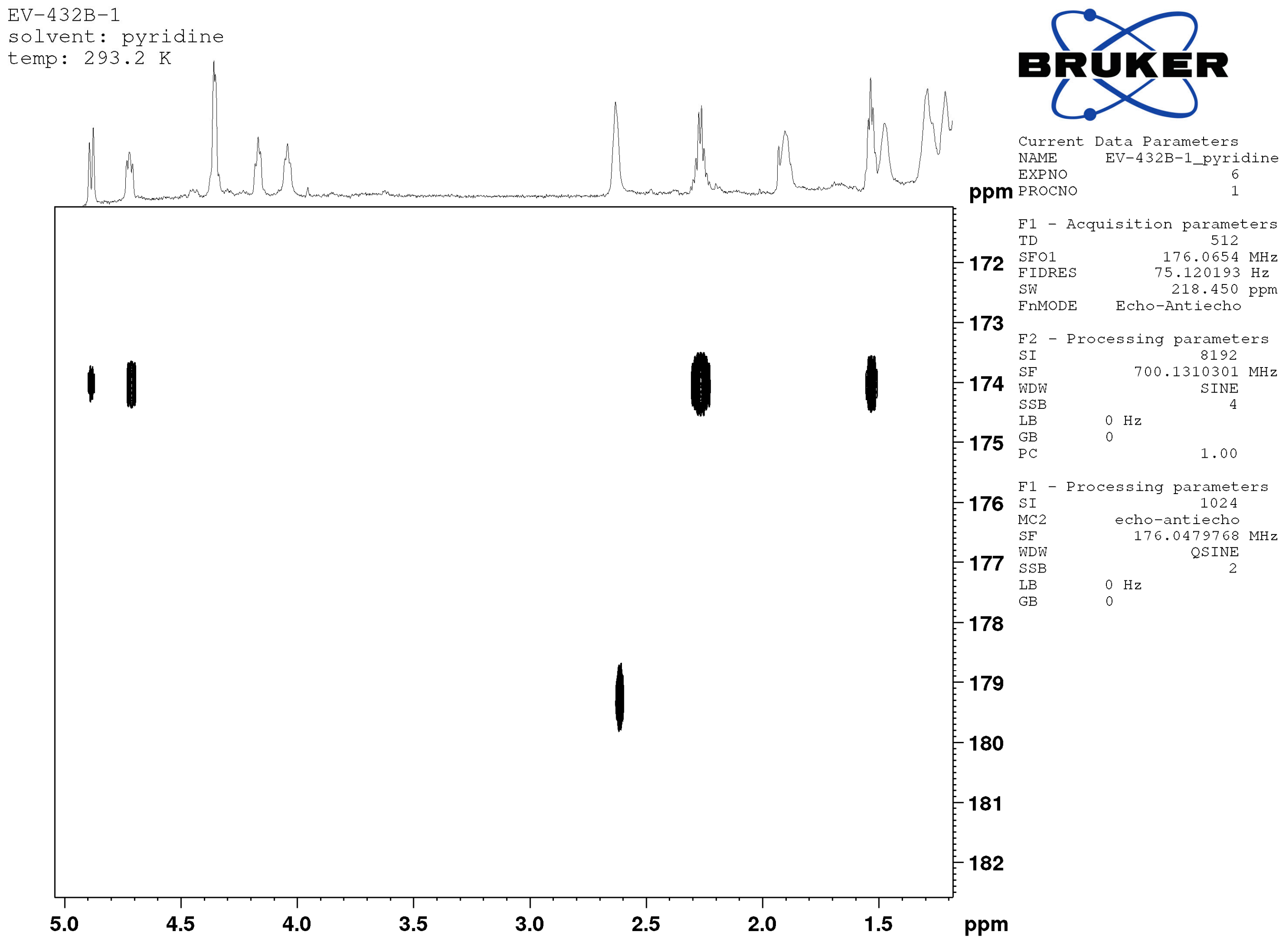

Figure S82. Detail (5/5) of HMBC NMR spectrum of compound **11**.

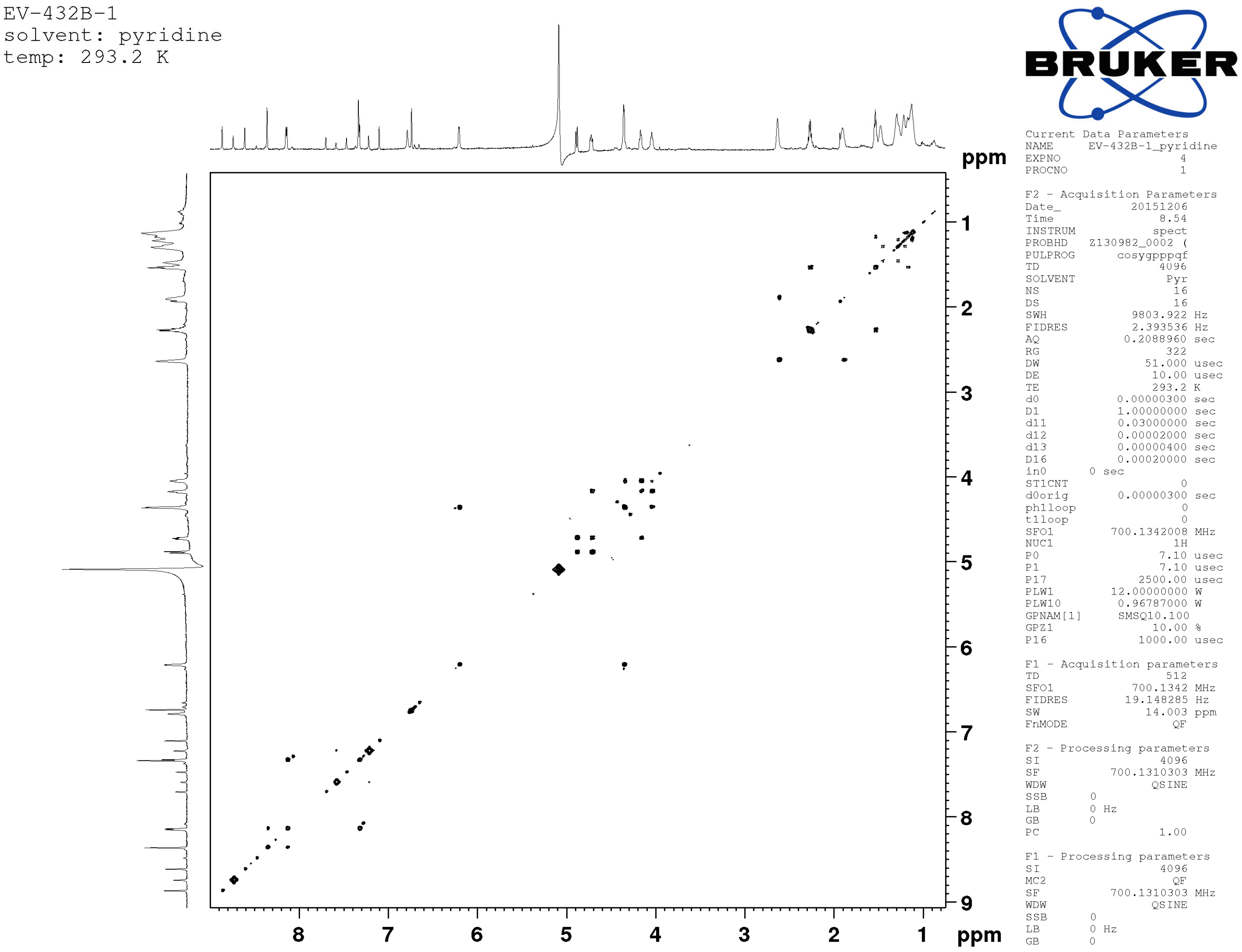

Figure S83. COSY NMR spectrum of compound **11**.

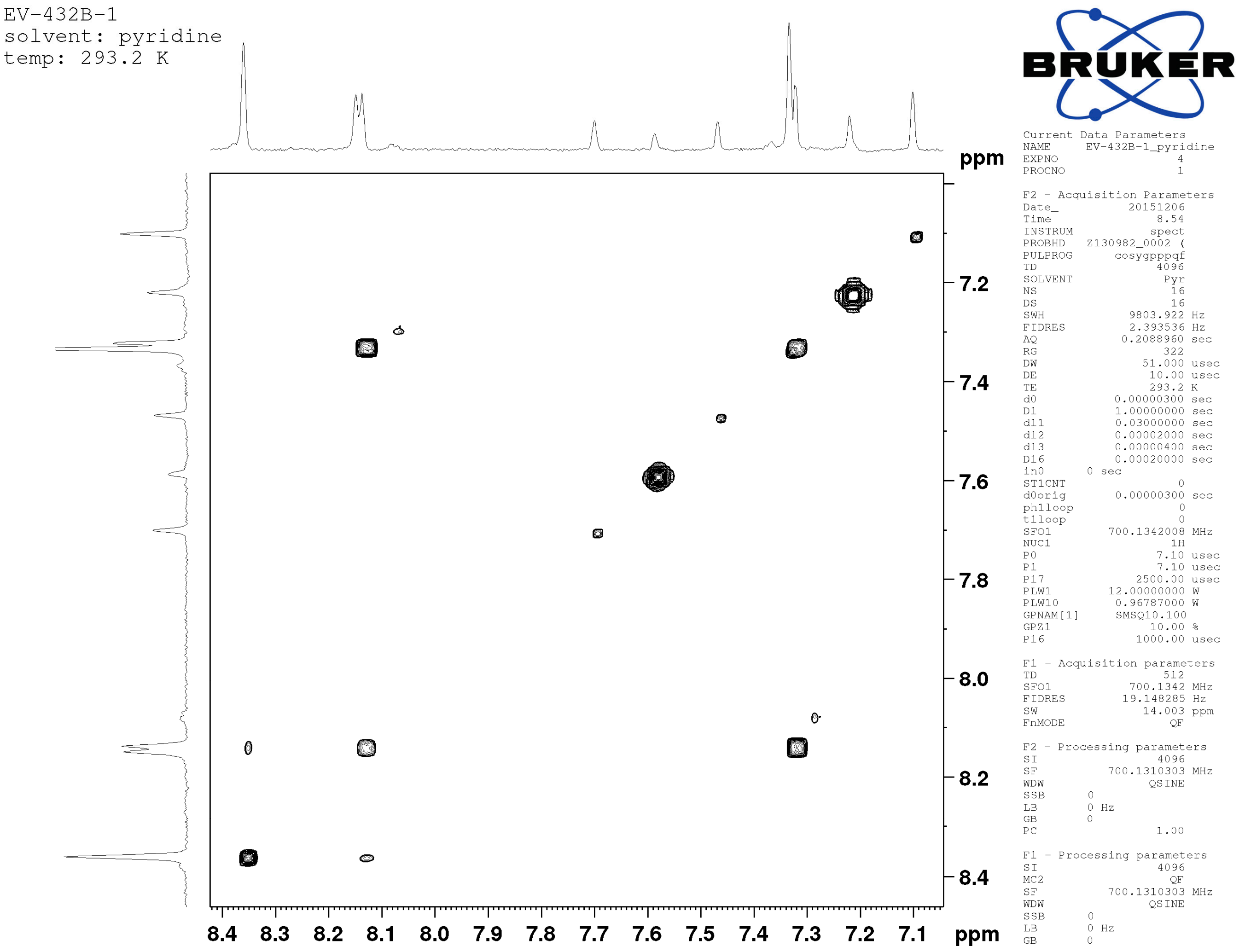

Figure S84. Detail (1/3) of COSY NMR spectrum of compound **11**.

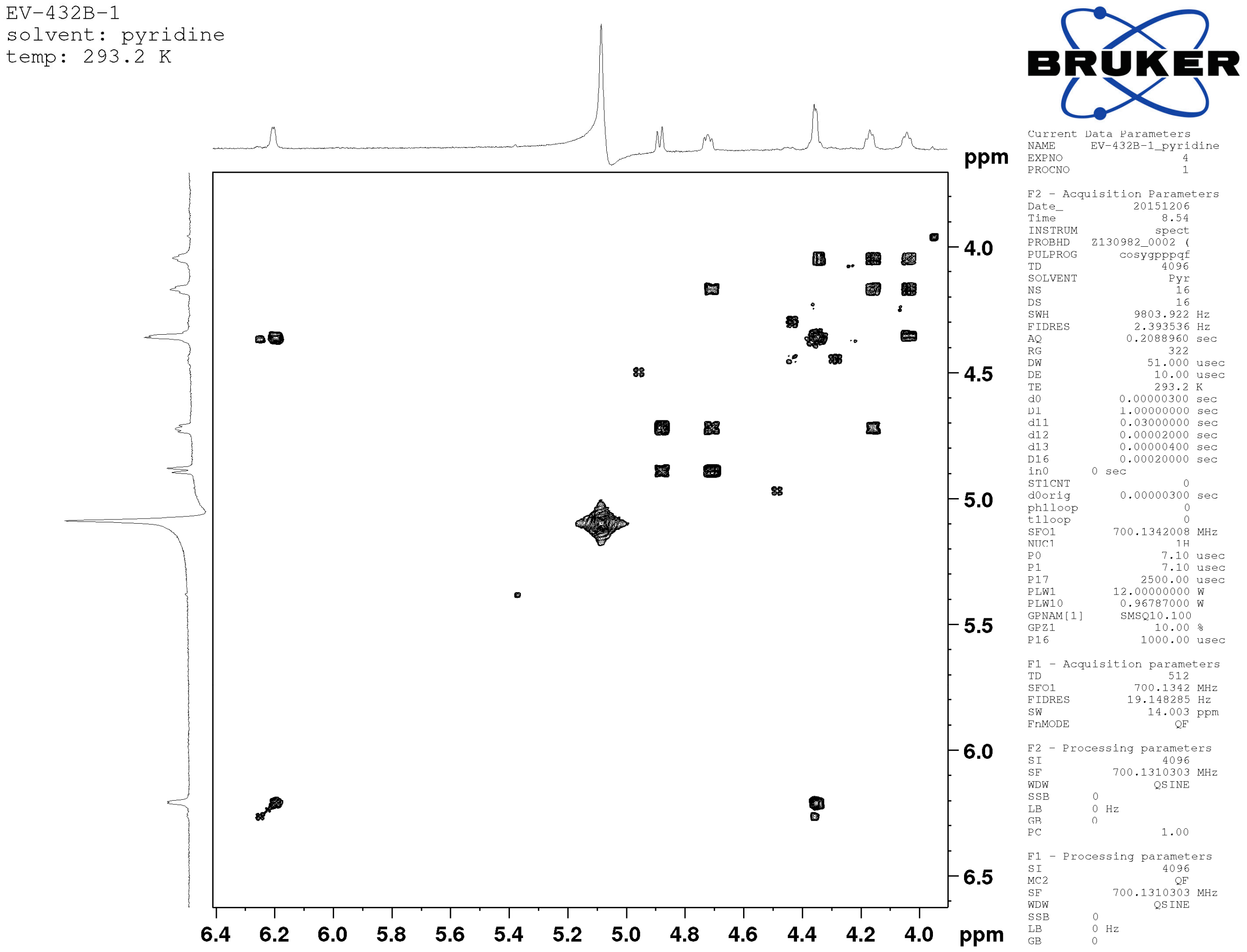

Figure S85. Detail (2/3) of COSY NMR spectrum of compound **11**.

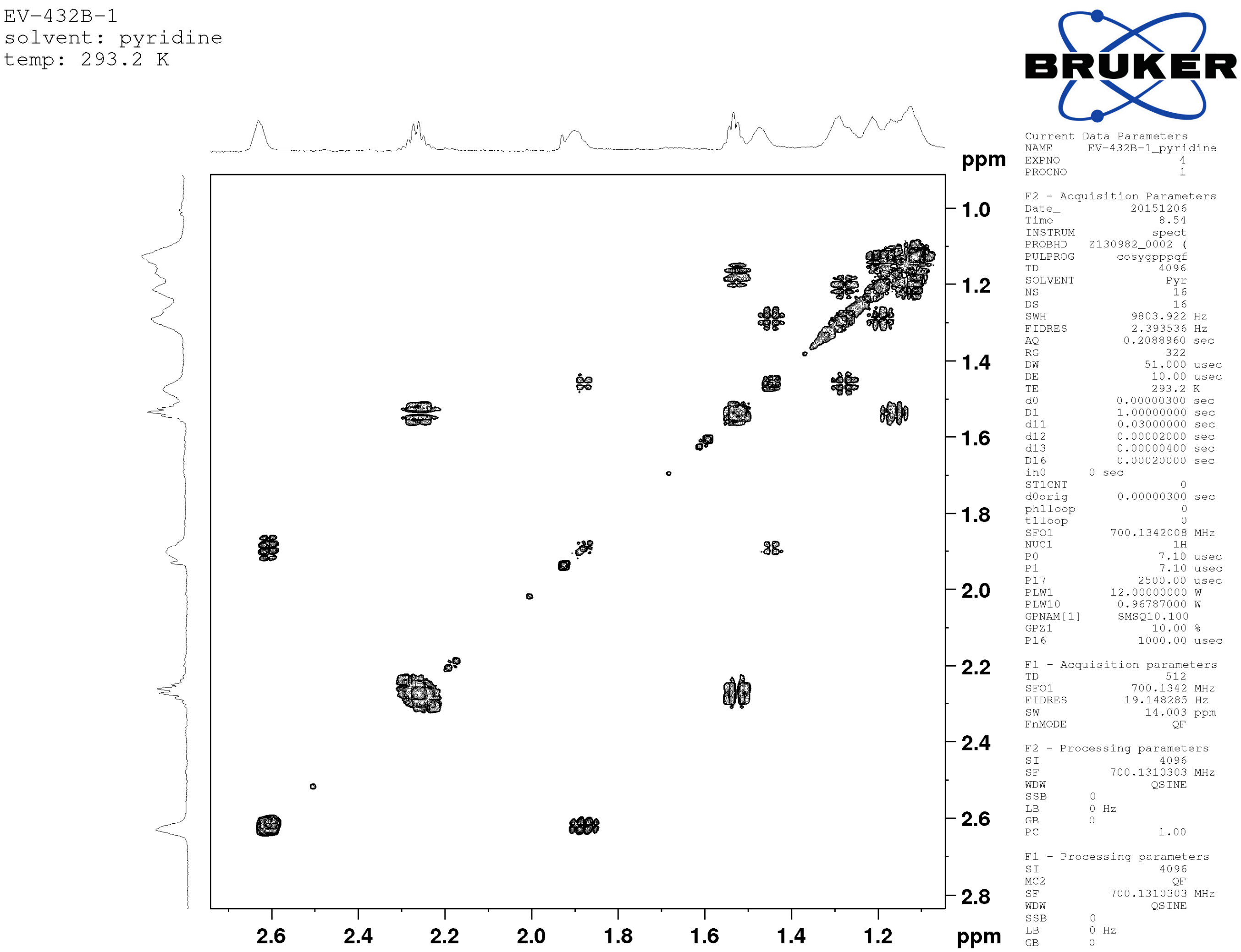

Figure S86. Detail (3/3) of COSY NMR spectrum of compound **11**.

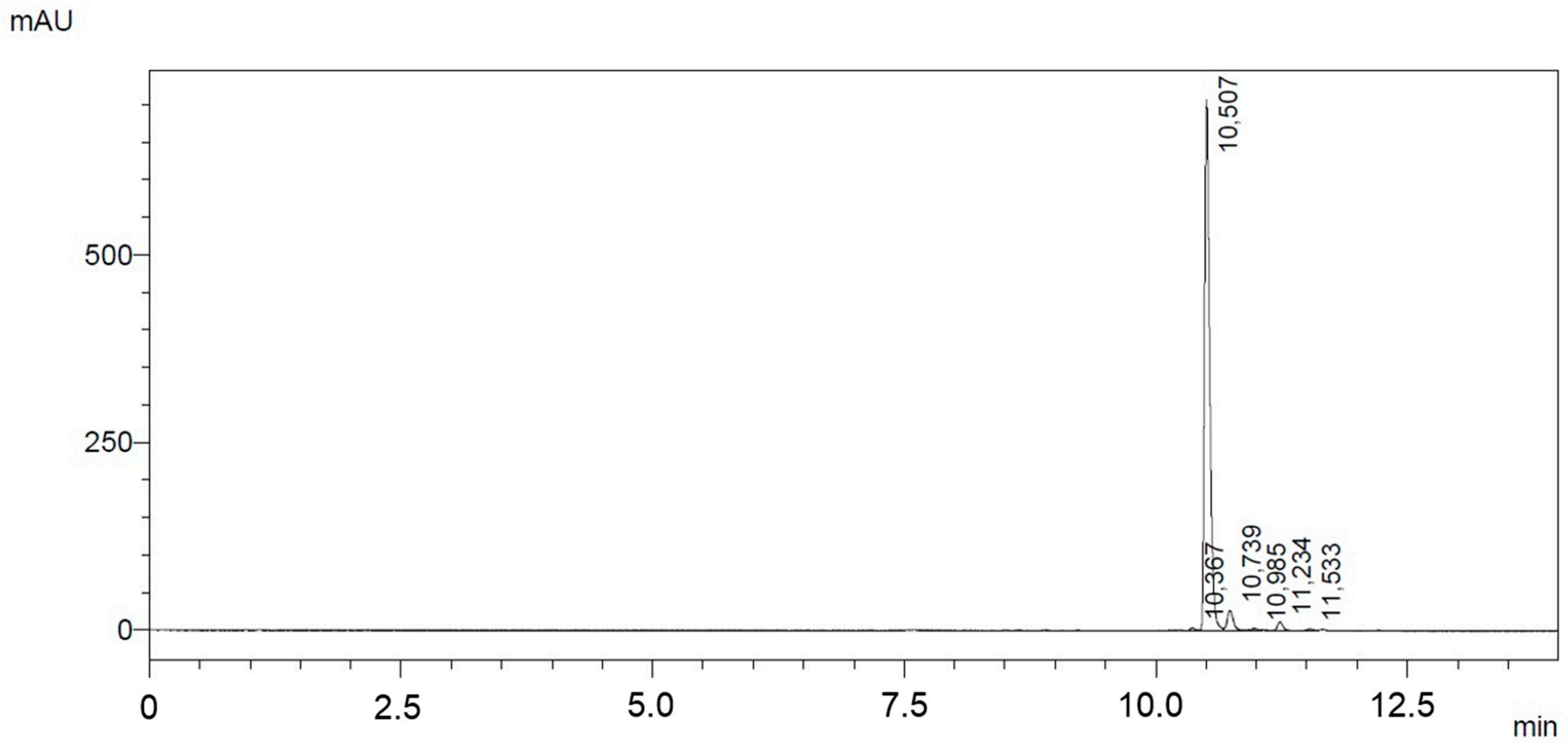

Figure S87. HPLC chromatogram of compound **11**.

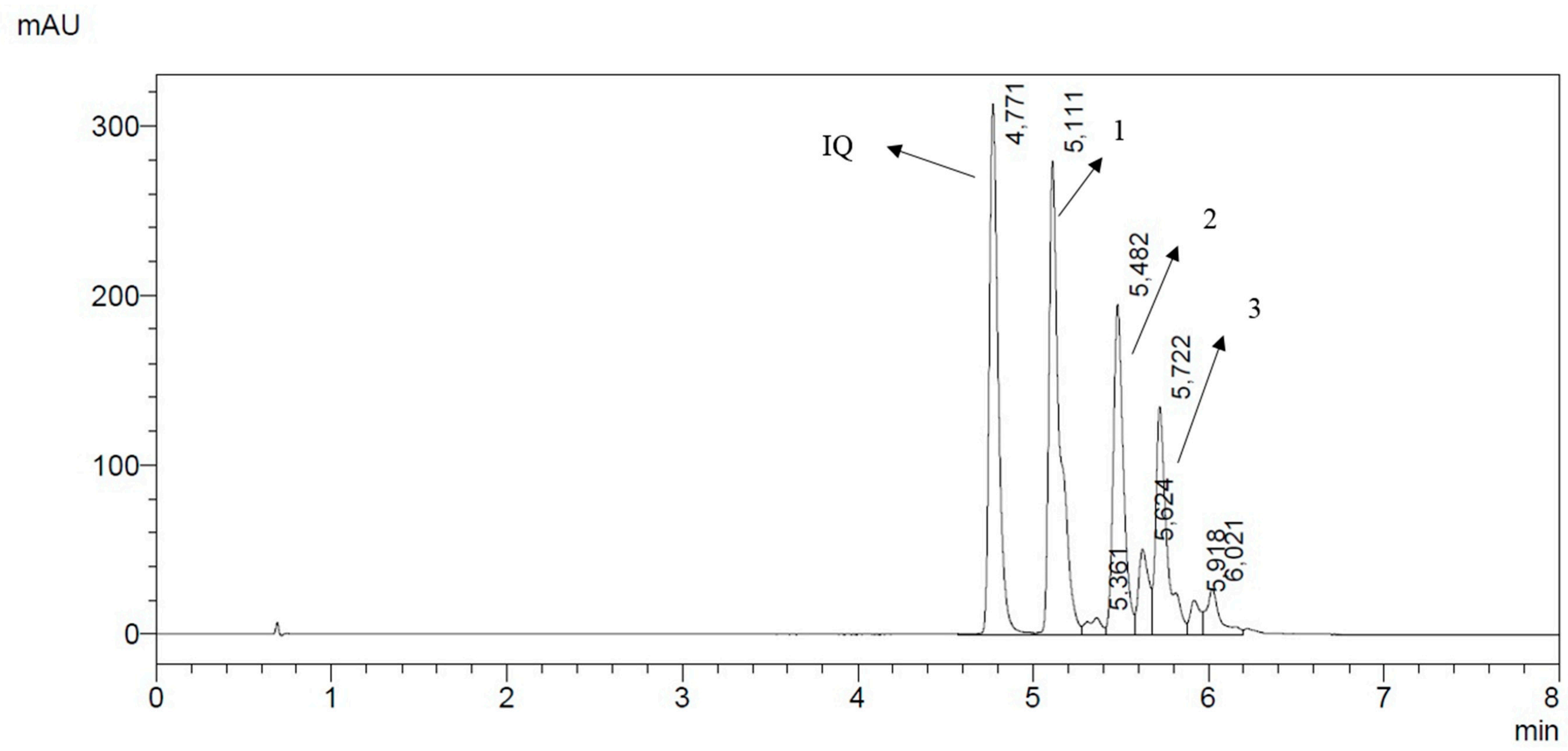

Figure S88. HPLC PDA chromatogram of the reaction mixture of isoquercitrin, succinic anhydride in the presence of Novozym 435®, acetone, 45 °C, 24 h. Peak 1: monosuccinate of isoquercitrin; Peak 2: disuccinate of isoquercitrin; Peak 3: trisuccinate of isoquercitrin.

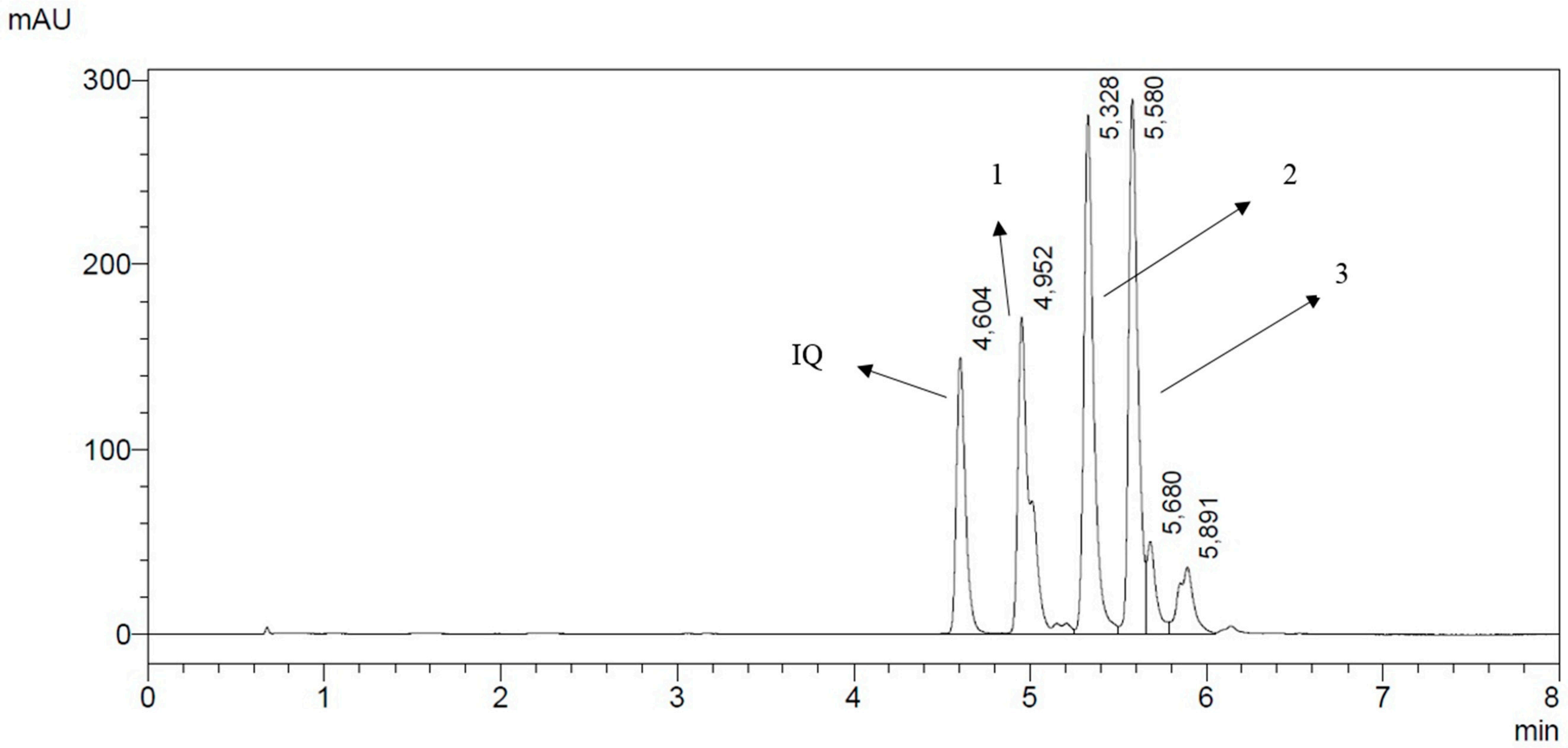

Figure S89. HPLC PDA chromatogram of the reaction mixture of isoquercitrin, succinic anhydride in the absence of Novozym 435®, acetone, 45 °C, 24 h. Peak 1: monosuccinate of isoquercitrin; Peak 2: disuccinate of isoquercitrin; Peak 3: trisuccinate of isoquercitrin.

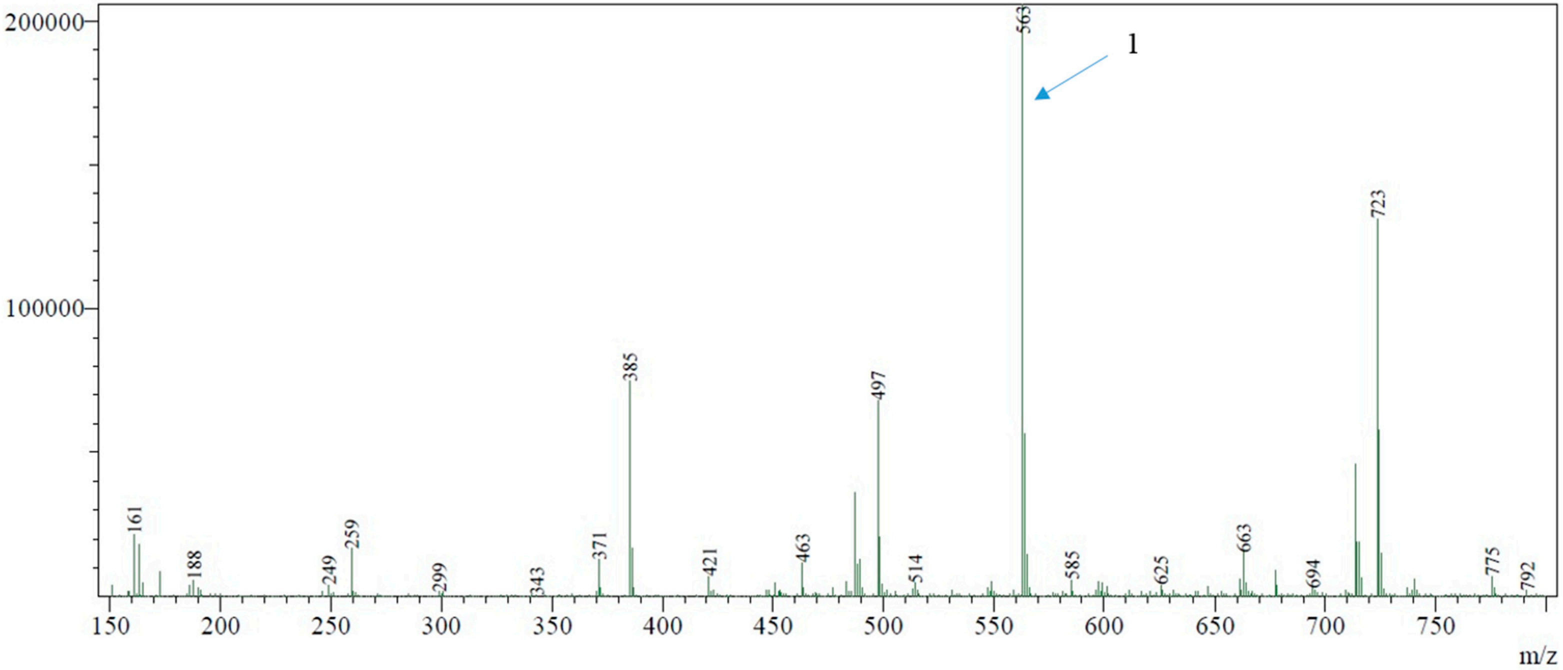

Figure S90. HPLC-MS (−) chromatogram of the monosuccinate of isoquercitrin (peak 1).

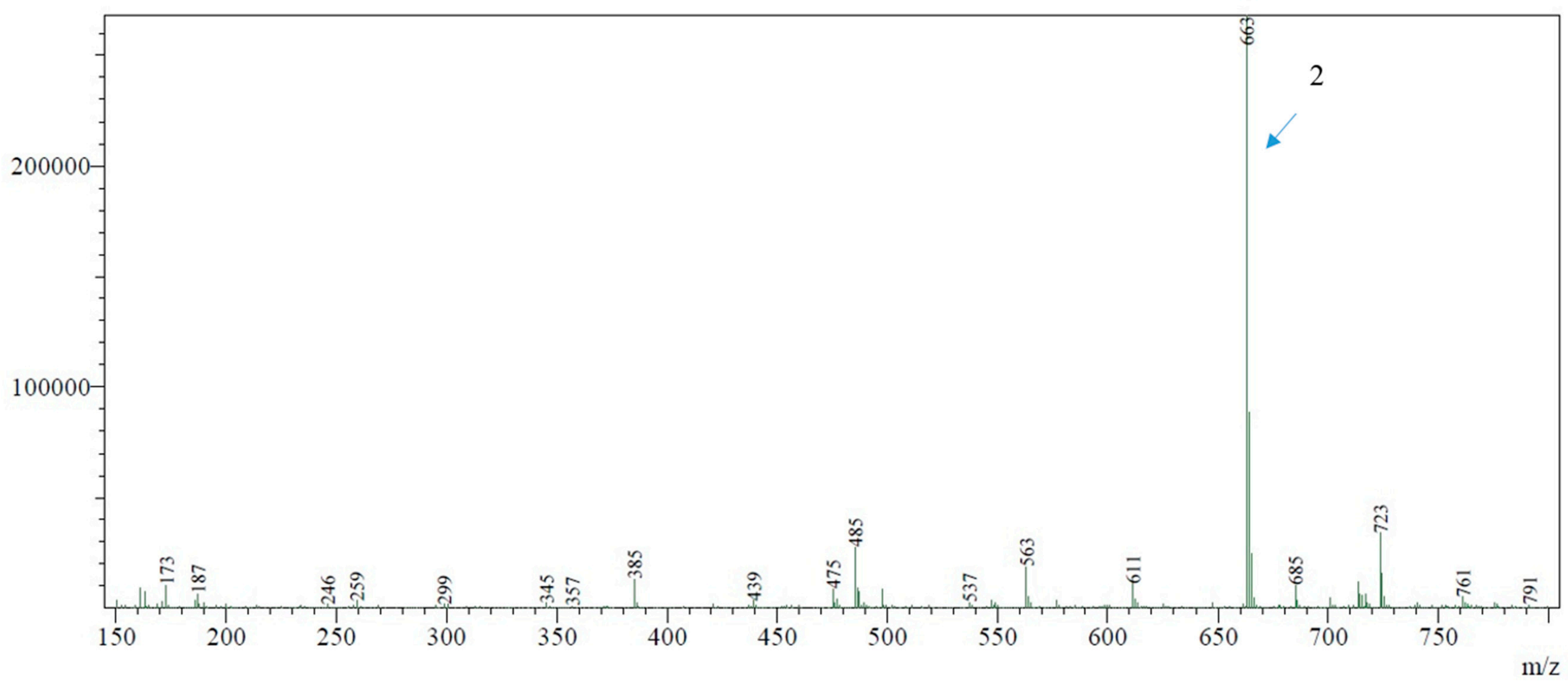

Figure

Figure S91. HPLC-MS (−) chromatogram of the disuccinate of isoquercitrin (peak 2).

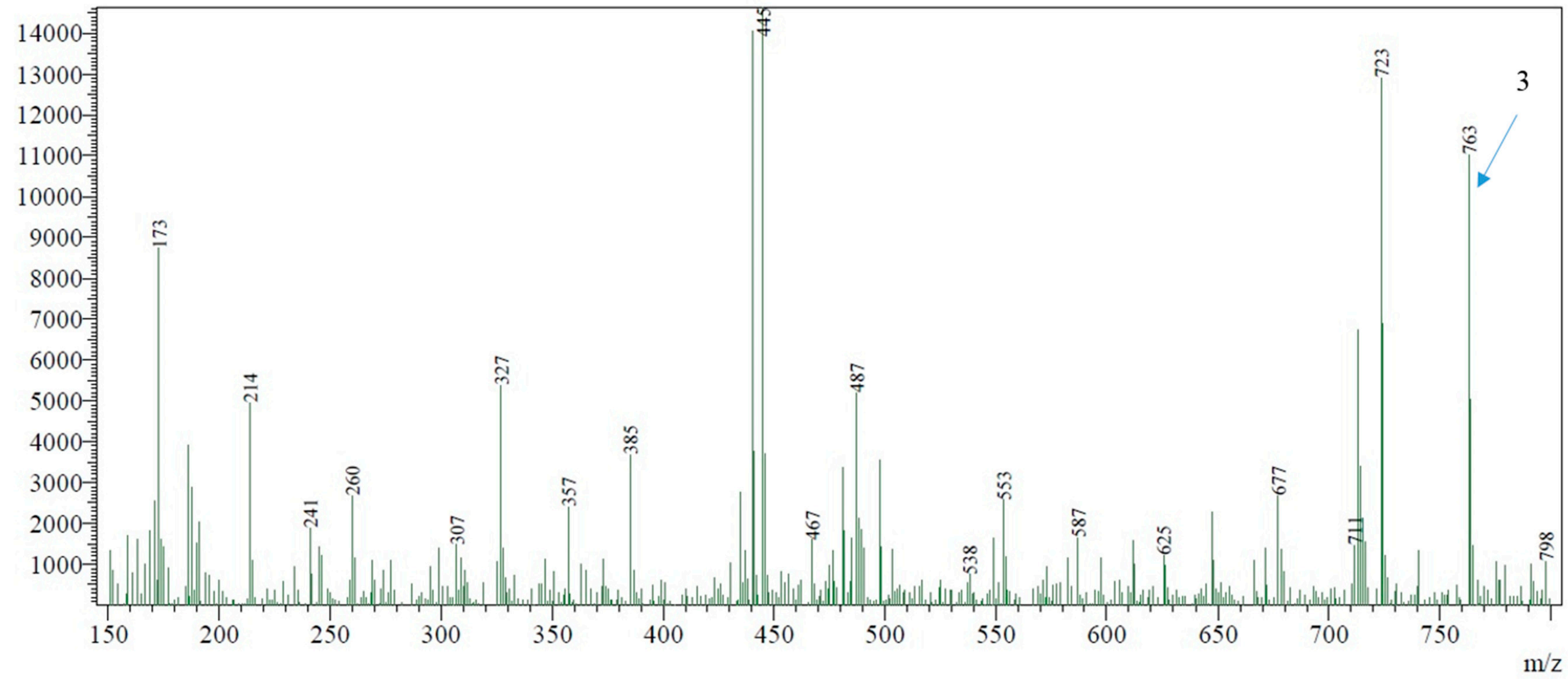

Figure S92. HPLC-MS (−) chromatogram of the trisuccinate of isoquercitrin (peak 3).
